# Effects of different exercise modalities and intensities on body composition in overweight and obese children and adolescents: a systematic review and network meta-analysis

**DOI:** 10.3389/fphys.2023.1193223

**Published:** 2023-07-11

**Authors:** Zan Huang, Jiayu Li, Yanjie Liu, Yulan Zhou

**Affiliations:** College of Physical Education and Health Sciences, Zhejiang Normal University, Jinhua, China

**Keywords:** exercise, body composition, obesity, network meta-analysis, children, adolescents

## Abstract

**Introduction:** Childhood and adolescent overweight and obesity are global public health issues. Previous studies on exercise and overweight and obese children have produced inconsistent findings and lacked comparisons between different exercise modalities and intensities. Therefore, a network meta-analysis is necessary to provide evidence-based intervention programs. This study aims to identify the effects of different exercise modalities and intensities on changes in body composition in overweight and obese children and adolescents.

**Methods:** A search for randomized controlled trials was conducted on Web of Science, PubMed, Scopus, and Embase involving exercise interventions aimed at improving body composition (body fat percentage, BMI, fat mass, fat-free mass, body weight) in overweight and obese children and adolescents. A random effects network meta-analysis was performed using STATA 14.0 software within a frequentist framework. The literature quality was assessed using the Cochrane Risk of Bias Tool 2.0.

**Results:** Thirty-two papers involving 1,452 participants were included. There were six types of intervention involved in the study, including moderate intensity aerobic exercise, high-intensity aerobic exercise, moderate intensity resistance exercise, high-intensity resistance exercise, moderate intensity combined exercise, and high-intensity combined exercise. The network meta-analysis results revealed that high-intensity combined exercise was the best exercise mode for improving BMI [mean difference in kg/m^2^ = −1.65, 95% CI (−3.27, −0.02)] and reducing fat mass [mean difference in kg = −2.87, 95% CI (−4.84, −0.91)]. Moderate intensity combined exercise was the best mode for weight loss [mean difference in kg = −4.58, 95% CI (−5.94, −3.22)] and improvement in body fat percentage [mean difference in% = −2.52, 95% CI (−3.83, −1.20)]. High-intensity resistance exercise had the optimal effect in increasing fat-free mass [mean difference in kg = 1.10, 95% CI (0.22, 1.99)].

**Conclusion:** In conclusion, the study found that combined exercise, whether moderate or high intensity, was more effective than any other exercise modality in improving body fat percentage and BMI, reducing fat mass and weight. Resistance exercise was the most effective in increasing fat-free mass.

## 1 Introduction

Over the last five decades, overweight and obesity have evolved from a minor public health issue affecting developed countries to a major public health concern with a global reach. According to the Global Burden of Disease Study 2013, the prevalence of overweight and obesity in children and adolescents was over 23% in developed countries and over 13% in developing countries. In 2019, the World Obesity Federation estimated that by 2025, 206 million children and adolescents aged 5–19 will be obese, with that number increasing to 254 million in 2030 (Lobstein and Brinsden, 2019). The total direct and lifetime costs resulting from obesity and overweight are estimated at 144,200 Euros, posing a significant economic challenge to children, adolescents, their parents, society, and the state (Hamilton et al., 2018).

Overweight and obese children and adolescents face both immediate and long-term health risks. They are more likely to have cardiovascular and metabolic disease risk factors, such as high triglycerides, hypertension, and high cholesterol, with 70% having at least one risk factor (Freedman et al., 2007). Obesity is often accompanied by various metabolic syndrome manifestations such as hyperinsulinemia, insulin resistance, and abnormal glucose tolerance (Sinha et al., 2002). It is a strong predictor of the development of type 2 diabetes and can cause effects that extend into adulthood, especially in children and adolescents with severe obesity (Daniels et al., 2005; Kelly et al., 2013). Additionally, being overweight and obese has been the most common cause of social exclusion and bullying, leading to poor mental health, impaired social development and education, and high rates of self-harming behavior and suicide among overweight and obese children and adolescents (Pont et al., 2017; Puhl and Lessard, 2020; Jebeile et al., 2022).

Given the enormous medical and health costs associated with overweight and obesity in children and adolescents, identifying effective palliative and intervention measures is a crucial priority for public health agencies. Currently, exercise and dietary interventions, in addition to surgical and pharmacological interventions, are proving to be effective ways to prevent and treat overweight and obesity (Peirson, Fitzpatrick-Lewis, Morrison, Ciliska, et al., 2015; Peirson, Fitzpatrick-Lewis, Morrison, Warren, et al., 2015). Treating obesity and promoting health through exercise is one of the most significant health and fitness trends worldwide (Stewart and Sutton, 2012). Body composition, which includes fat mass, fat-free mass, body fat percentage, and body mass index, is a crucial factor in obesity. Adverse changes in body composition and weight gain are key features of obesity. Multiple and inconsistent findings exist on how exercise can affect the body composition of overweight and obese children and adolescents. Previous studies have used traditional pairwise comparative meta-analyses to determine the effects of different exercise modalities (such as aerobic, resistance, and combined exercise) on body composition (Schranz et al., 2013; Kelley et al., 2015; Méndez-Hernández et al., 2022). However, direct comparisons between these modalities still need to be made. Additionally, differences in the effects of exercise intensities on body composition in overweight and obese children and adolescents have been observed (Gutin et al., 2002; Lazzer et al., 2011). Therefore, the optimal exercise pattern (format and intensity) for improving body composition in this population remains unclear.

To address this research gap, we propose to use Network Meta-Analysis (NMA), which is an extension of the traditional head-to-head meta-analysis. NMA allows for the simultaneous comparison of the efficacy of three or more interventions (Watt and Del Giovane, 2022) and can analyze both direct and indirect comparisons (Li et al., 2011). When direct comparisons are lacking, NMA can compare the effects of two treatment measures indirectly through a common control. When direct comparisons exist, NMA combines the results of direct and indirect comparisons, thereby increasing the accuracy of the results (Jansen et al., 2012).

Therefore, in this study, we aim to use NMA to explore the effects of different exercise patterns on body composition in overweight and obese children and adolescents. Our goal is to provide an evidence-based basis for selecting effective intervention programs for this population.

## 2 Materials and methods

### 2.1 Protocol and registration

This systematic review was conducted in accordance with the Preferred Reporting Items for Systematic Reviews and Meta-Analyses (PRISMA) statement and was registered in the PROSPERO database under the registration number CRD42023403600. The results are reported in compliance with the extended items of the network meta-analysis as recommended by PRISMA (Hutton et al., 2015). Additionally, specific aspects of the network meta-analysis were reported in accordance with the guidelines set forth by Chaimani et al. (2017) (as shown in Supplementary Table S1).

### 2.2 Search strategy

We conducted a comprehensive search of the Web of Science, PubMed, Embase, and Scopus databases for literature on exercise interventions aimed at improving body composition in overweight and obese children and adolescents, published by January 2023. Search terms included: exercise OR physical activity OR aerobic exercise OR muscle stretching exercise OR physical exercise OR sport* OR aerobic OR resistance OR movement OR workout OR strength training OR combined training OR endurance training OR concurrent training OR circuit training OR HIIT training OR interval training AND body weight OR body mass index OR BMI OR body fat percentage OR body fat OR fat mass OR body composition OR weight loss OR body mass OR obesity OR overweight OR obese OR waist circumference AND child* OR children OR adolescent* OR juvenile* OR teen* OR teenager* OR youth OR student*. In addition, we conducted a manual search of all article references to supplement the literature missed by the computer search. Detailed search strategies for each database are available in the Supplementary Table S2.

### 2.3 Eligibility criteria

Inclusion criteria for this systematic review were established based on the PICOS principles (population, intervention, comparator, outcomes, and study type).

Population (P): Participants aged 5–18 years, without any musculoskeletal disorders or clinical contraindications to exercise, who were overweight or obese were eligible for inclusion.

Intervention (I): Any exercise intervention trials were included.

Comparators (C): Participants in the control group were required to maintain their current level of physical activity or perform a different intensity and/or form of exercise.

Outcomes indicators (O): Outcome measures related to body composition indicators, such as fat mass, body fat percentage, body mass index, fat-free mass, and weight, were considered.

Study type (S): Only randomized controlled trials (RCTs) published in English were eligible for inclusion.

Exclusion criteria.

1. Studies involving mixed samples of individuals with overweight/obesity and other non-communicable diseases.
2. Studies that included people aged >18 and <5 years.
3. Studies in which the effects of exercise interventions could not be isolated because they were part of a multi-component intervention (e.g., diet and exercise interventions).
4. Studies that did not provide details of the intensity of the exercise intervention.
5. Studies that did not evaluate the outcomes of interest of the article.
6. Studies that did not provide mean and standard deviation or data that could be translated into mean and standard deviation form.
7. Non-peer-reviewed English, abstracts, reviews, conference reports.
8. Studies for which the full text was not available.

### 2.4 Study selection

Endnote X9 literature management software was used to manage the literature search records. After removing duplicates, the remaining studies were screened independently by two authors (ZH and YL) using predefined inclusion and exclusion criteria, with any disagreements resolved by consensus. During the initial screening, the authors analyzed the titles and abstracts of all literature to identify potentially relevant literature. Subsequently, the full texts of potentially relevant studies were obtained and carefully read to identify the studies that met the inclusion and exclusion criteria. Finally, two authors discussed the final list of included studies, and a third author (YZ) was consulted to determine which studies could ultimately be included in this systematic review.

### 2.5 Data extraction

Two investigators extracted relevant data from the eligible literature. Extracted data included first author, year of publication, country, intervention duration, sample size, mean age, exercise intervention details (frequency, intensity, time, type), and outcome indicators. The classification of exercise intensity categories was based on the ACSM Guidelines for Exercise Testing and Exercise Prescription 9th edition for aerobic and resistance exercise intensity estimates (Medicine, 2013). High-intensity aerobic exercise was defined as >65% VO2max or >65% HRR or >75% HRmax or PRE of more than 5, and moderate intensity as 45%–65% VO2max or 50%–65% HRR or >65–75% HRmax or PRE of 3–4. High-intensity resistance training was defined as >75% of a one maximum load (1RM) or less than 10 repetition maximum, and moderate intensity as 50%–75% of a 1RM or 10–15 repetition maximum. The intensity of each exercise is described in detail in Table 1.

**TABLE 1:** Definition of each intervention.

Type of exercise	Definition
**Moderate-intensity aerobic exercise**	Intensity: 45%–65% VO_2max_ or 50%–65% HRR or >65–75% HR_max_ or PRE of more than 5 Type: Forms of aerobic exercise (e.g., walking, running, bicycling, rowing, swimming, aerobics, elliptical exercise, and stepping)
**High-intensity aerobic exercise**	Intensity: >65% VO_2max_ or>65% HRR or >75% HR_max_ or PRE of more than 3–4 Type: Forms of aerobic exercise (e.g., walking, running, bicycling, rowing, swimming, aerobics, elliptical exercise, and stepping)
**Moderate-intensity resistance exercise**	Intensity: 50%–75% of 1RM or 10–15 repetition maximum before muscle fatigue sets in Type: Forms of resistance training (e.g., free strength training, weight machines, and resistance band training)
**High-intensity resistance exercise**	Intensity: >75% of 1RM or less than 10 repetition maximum before muscle fatigue sets in Type: Forms of resistance training (e.g., free strength training, weight machines, and resistance band training)
**Moderate-intensity combined exercise**	Moderate-intensity aerobic combined with moderate-intensity resistance exercise
**High-intensity combined exercise**	High-intensity aerobic combined with high-intensity resistance exercise

HR_max_, maximum heart rate; HRR, heart rate reserve; RM, repetition maximum; VO_2max_, maximal oxygen uptake. PRE, perceived effort.

### 2.6 Risk of bias assessment

Two authors (ZH and JL) independently assessed the risk of bias. The Cochrane tool for assessing the risk of bias in randomized trials Version 2 (RoB2) was used to perform the risk of bias assessment (Sterne et al., 2019). RoB2 comprises five evaluation domains: randomization process, deviation from intended interventions, missing outcome data, outcome measurement, and selection of reported result. Each domain has several signal questions, and the risk of bias is classified as “low risk,” “some concerns,” and “high risk” based on the assessor’s responses to the signal questions. The overall risk was determined based on the risk of bias rating for each of the five domains. If the risk of bias in all domains is evaluated as “low risk,” then the overall risk of bias is “low risk.” If the risk of bias in a domain is “some concerns,” and there is no domain with “high risk,” then the overall risk of bias is “some concerns.” If a domain with a risk of bias is rated as “high risk,” then the overall risk of bias is “high risk.”

### 2.7 Evidence assessment

The CINeMA (Confidence In Network Meta-Analysis) model was used to comprehensively assess the quality of evidence for the results of the entire network analysis (Papakonstantinou et al., 2020). The following six domains were considered: within-study bias (risk of bias), between-study bias (publication bias or reporting bias), indirectness, imprecision, heterogeneity, and incoherence. Each domain was graded as “no concern,” “some concern,” or “major concern” according to its severity, and the final NMA evidence quality rating was consistent with the GRADE system and classified as high, moderate, low, or very low (Nikolakopoulou et al., 2020).

### 2.8 Statistical analyses

The raw data were pre-processed using Microsoft Office Excel. The required data were converted to the mean and standard deviation of the before and after differences according to the formula provided in the Cochrane Handbook for Systematic Review (Higgins et al., 2019). The formulas are as follows:

$${Mean}_{difference}={Mean}_{final}-{Mean}_{baseline}\;SD=\sqrt{SD_{baseline}^{2}+SD_{final}^{2}-2\times Corr\times SD_{baseline}\times SD_{final}}$$

$$Corr=\left({SD}_{baseline}^{2}+2{SD}_{final}^{2}-{SD}_{change}^{2}\right)/2\times {SD}_{baseline}\times {SD}_{final}$$

The mean difference and its 95% confidence interval (CI) were used for merged statistics. A pairwise meta-analysis of all outcome indicators was performed using a random-effects model to explore the relative effects of different exercise interventions. A *Q* test and *I* ^*2*^ statistic were used to quantify heterogeneity between included studies, with a significance level of *p* < 0.1. *I* ^*2*^ values of 25%, 50%, and 75% represent low, moderate, and high heterogeneity, respectively (Higgins and Thompson, 2002).

Network meta-analysis was performed with STATA 14.0 in a frequentist framework. Global inconsistency between interventions was assessed using the Wald test, and local inconsistency between direct and indirect comparisons between each intervention was assessed using the node-splitting method. The consistency model was used if both global and local consistency assumptions were satisfied. Network evidence was plotted for interventions with different intensity exercise modalities. When there was a closed-loop structure, the Loop Inconsistency Test was performed to check the inconsistency of the closed-loop structure, and the inconsistency factors, 95% CI, and *p* values were calculated. Intervention efficiency was ranked using the surface under the cumulative ranking curve (SUCRA), with SUCRA values ranging from 0% to 100%. The closer the SUCRA value is to 100%, the better the intervention will be.

## 3 Results

### 3.1 Study selection

**FIGURE 1:**
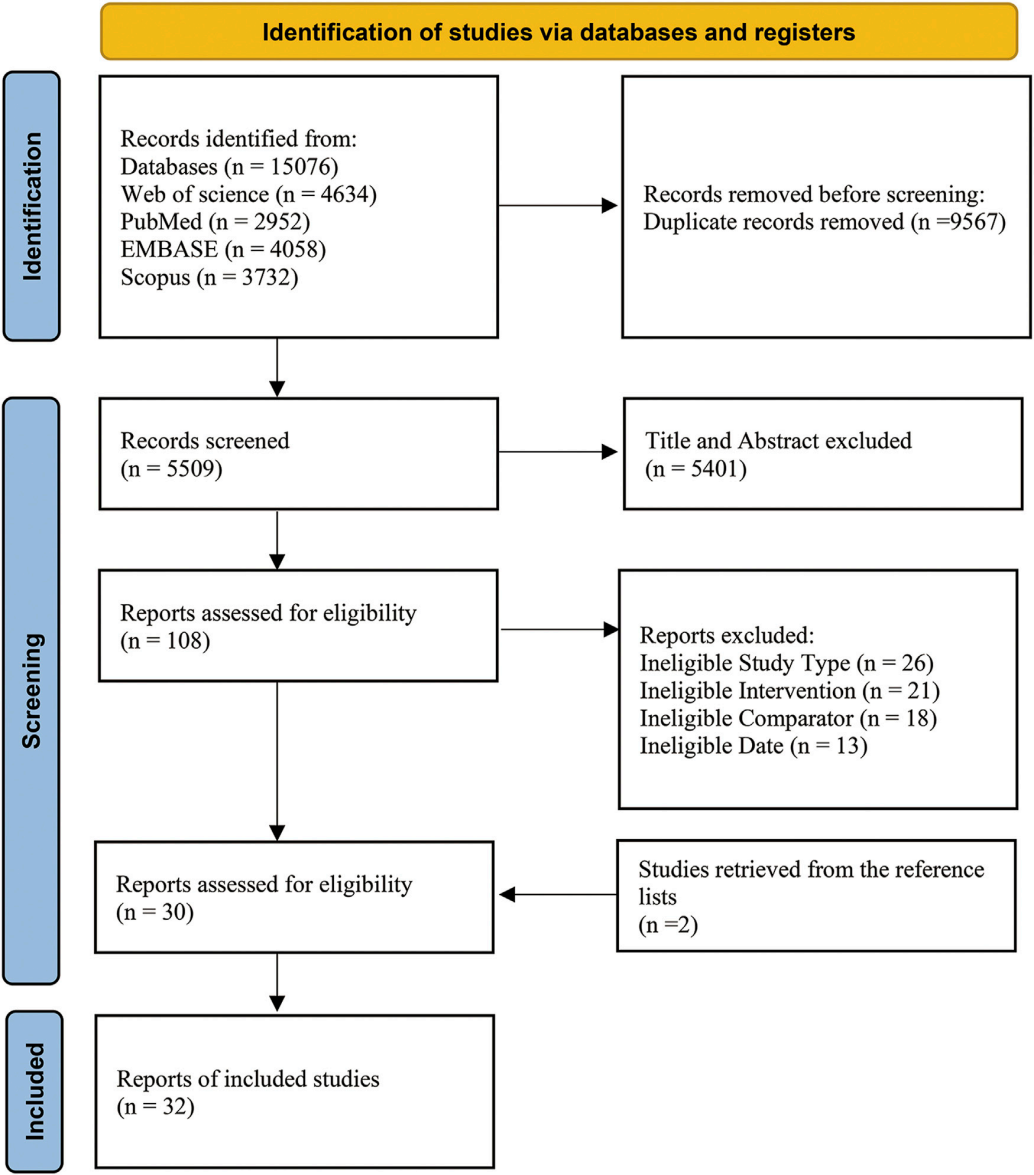
Flow of information through the different phases of the systematic review.

The search resulted in 15,076 studies. After removing duplicate literature, 5,509 studies remained. After screening the titles and abstracts, 108 studies were read in full text for refinement. Then, 30 studies met the inclusion and exclusion criteria. After searching the reference list from relevant papers and reviews, two studies were obtained. The final meta-analysis included 32 studies (Figure 1).

### 3.2 Study characteristics

Thirty-two studies included in the network meta-analysis were published from 2006 to 2022. The regions of publication involved Europe (*n* = 5) (Farpour-Lambert et al., 2009; Giovanni et al., 2017; Cvetković et al., 2018; Martín-García et al., 2019; Salus et al., 2022), Asia (*n* = 15) (Wong et al., 2008; 2018; Karacabey, 2009; Lee et al., 2010; 2012; 2013; Saygın, 2011; Song et al., 2011; Jeon et al., 2013; Youssef et al., 2015; Tan et al., 2016; Tan et al., 2017; Kim et al., 2019; Said et al., 2021; Cao et al., 2022), Africa (*n* = 4) (Regaieg et al., 2012; Racil et al., 2016; Khammassi et al., 2018; Bouamra et al., 2022), North America (*n* = 4) (Shaibi et al., 2006; Alberga et al., 2013; Sigal et al., 2014; Deldin et al., 2019), South America (*n* = 3) (Monteiro et al., 2015; Vasconcellos et al., 2015; Duft et al., 2020), and Oceania (*n* = 1) (Dias et al., 2018). The total sample included in the analysis was 1,452 individuals. Exercise interventions included aerobic, resistance, and aerobic combined with resistance exercise. Fourteen studies involved high-intensity aerobic exercise (AE-HI), 16 studies involved moderate-intensity aerobic exercise (AE-M), four studies involved high-intensity resistance exercise (RT-HI), seven studies involved moderate-intensity resistance exercise (RT-M), three studies involved high-intensity combined exercise (COM-HI), six studies involved moderate-intensity combined exercise (COM-M), and 29 studies involved exercise interventions not received by the control group (CON). The mean intervention duration was 12.67 ± 2.58 weeks, ranging from 9 to 22 weeks, with 75.8% of the studies having a 12-week intervention duration. The average session was 3.06 ± 0.75 times per week, and the average time per session was 57.78 ± 13.14 min. Outcome indicators included body weight, BMI, fat mass, body fat percentage, and fat-free mass. The details of the included literature’s characteristics are shown in Table 2.

**TABLE 2:** Basic characteristics of the included literature.

Study	Country	Duration, (weeks)	Sample	Mean age	Exercise category	Summary description of exercise intervention, frequency, intensity, time, and type (F.I.T.T)	Outcome measures reported
Shaibi *et al.* (2006)	United States	16	11	15.6 ± 0.5	CON	no exercise	Weight, BMI, BFP, FM, FFM
			11	15.1 ± 0.5	RT-HI	2 d/week; week1–4: 62–71% of 1RM; weeks 5∼10: 74–88% of 1RM; weeks11∼16: 92–97% of 1RM; less than 60 min; periodized resistance exercise	
Wong *et al.* (2008)	Singapore	12	12	14.3 ± 1.5	CON	no exercise	Weight, BMI, BPF, FM, FFM
			12	13.8 ± 1.1	COM-M	2 d/week; 65%–85% HR_max_ and 55%–65% 1RM; 55 min; aerobic exercise and resistance training	
Karacabey (2009)	Turkey	12	20	11.2 ± 0.8	CON	no exercise	Weight, BMI
			20	11.8 ± 0.5	AE-M	3 d/week; 60%–65%HRR; 65 min; walking–jogging exercise	
Farpour-Lambert *et al.* (2009)	Switzerland	12	22	8.8 ± 1.6	CON	no exercise	Weight, BMI, BPF, FFM
			22	9.1 ± 1.4	AE-M	3 d/week; 55%–65% HR_max_; 60 min; aerobic exercise	
Lee *et al.* (2010)	Korea	10	18	13.3 ± 1.2	CON	no exercise	BMI
			16	12.8 ± 1.5	AE-HI	3 d/week; 60%–80% VO_2max_ and 70%–90% HR_max_; 60 min; aerobic exercise	
			20	13.9 ± 1.2	COM-M	3 d/week; 70%–80% maximum strength; 60 min; circuit weight training and aerobic exercise	
Saygın (2011)	Turkey	12	19	10–12^a^	CON	no exercise	Weight, BMI, BPF
			20		AE-M	3 d/week; 50%–60% HR_max_; 60–90 min; aerobic exercise	
Song *et al.* (2011)	Korea	12	10	12.6 ± 0.2	CON	no exercise	Weight, BMI, BPF, FM, FFM
			12	12.7 ± 0.2	AE-M	3 d/week; 60%–70% HR_max_; 50 min; aerobic exercise	
Lee *et al.* (2012)	Korea	12	13	14.8 ± 1.4	CON	no exercise	Weight, BMI, FM, FFM
			16	15.2 ± 1.9	AE-M	3 d/week; 50%–75% VO_2max_; 60 min; treadmills, ellipticals, or stationary bikes	
			16	14.6 ± 1.5	RT-M	3 d/week; 60% 1RM; 60 min; whole-body exercises	
Regaieg *et al.* (2012)	Tunisia	16	14	10.6 ± 0.7	CON	no exercise	Weight, BMI, BPF
			14	10.9 ± 0.6	AE-HI	1 day/week; 70%–85% HR_max_; 60 min; combination of games and sports activities	
Lee *et al.* (2013)	Korea		12	15.0 ± 2.2	CON	no exercise	Weight, BMI, BPF
			16	14.6 ± 1.9	AE-M	3 d/week; 50%–75% VO_2max_; 60 min; treadmills	
			16	14.8 ± 1.9	RT-M	3 d/week; 60% 1RM; 60 min; whole-body exercises	
Alberga *et al.* (2013)	Canada	12	7	10 ± 2	CON	no exercise	Weight, BMI, FM, BPF, FFM
			12	10 ± 1	RT-M	2 d/week; 65%–85% 1RM; 75 min; resistance exercise	
Jeon *et al.* (2013)	Korea	12	7	12.4 ± 0.7	CON	no exercise	Weight, BMI, FM, BPF
			8	13.1 ± 0.5	COM-M	2 d/week; 55%–75% HR_max_ walking exercise and 70% 1RM rubber band exercise; 85 min	
Sigal *et al.* (2014)	Canada	22	76	15.6 ± 1.3	CON	no exercise	FM. BPF, FFM
			75	15.5 ± 1.4	AE-HI	4 d/week; 65%–85% HR_max_; 20 min–45 min; treadmills, elliptical machines, or bicycle ergometers	
			78	15.9 ± 1.5	RT-HI	4 d/week; 2 sets×15 repetitions at moderate intensity and 3 sets of 8 repetitions at the maximum resistance; 20 min–45 min	
			75	15.5 ± 1.3	COM-HI	4 d/week; full aerobic training program plus the resistance training program; 20 min–45 min	
Youssef *et al.* (2015)	Lebanon	12	9	16.3 ± 0.5	CON	no exercise	Weight, BMI, BPF, FFM
			14	16.1 ± 0.3	AE-HI	3 d/week; 75%–75% HRR; 90 min; aerobic exercise and interval training	
Monteiro *et al.* (2015)	Brazil	20	16	11.04 ± 1.9	CON	no exercise	Weight, BMI, FM, BPF, FFM
			18	11.0 ± 1.02	AE-HI	3 d/week; 65%–85%VO_2max_; 50 min; walking and running	
			14	11.03 ± 1.34	COM-HI	3 d/week; 65%–85%VO_2max_ for aerobic exercise and 55%–75% RM for resistance training; 60 min; 50% of resistance training followed by 50% of the aerobic exercise	
Tan et al. (2016)	China	10	13	9.4 ± 0.9	CON	no exercise	Weight, BMI, FM, BPF, FFM
			11	9.4 ± 1.3	AE-M	5 d/week; HR of FAT_max_ intensity; 60 min; walking, running, and ball game	
Vasconcellos *et al.* (2015)	Brazil	12	10	14.8 ± 1.4	CON	no exercise	Weight, BMI, BPF
			10	14.1 ± 1.3	AE-HI	3 d/week; 84.5% ± 4.1% HR_max_; 60 min; recreational soccer program	
Racil *et al.* (2016)	Tunisia	12	14	14.2 ± 1.2	CON	no exercise	Weight, BPF
			16		AE-M	3 d/week; 80% maximal aerobic speed; 60 min; moderate-intensity interval training	
			17		AE-HI	3 d/week; 100% maximal aerobic speed; 60 min; high-intensity interval training	
Giovanni *et al.* (2017)	Italy	16	12	12.21 ± 0.43	AE-M	3 d/week; 45%–50% HRR; 60 min; cycle-ergometers and treadmills	Weight, BMI, BPF, FFM
			15	12.73 ± 0.70	RT-M	3 d/week; 13-RM; 60 min; isotonic machines	
			14	12.67 ± 0.65	RT-HI	3 d/week; 9-RM; 60 min; isotonic machines	
Tan *et al.* (2017)	China	10	21	5.1 ± 0.4	CON	no exercise	Weight, BMI, FM, BFP, FFM
			21		AE-M	5 d/week; 50% HRR; 60 min; quick walking, slow running, jumping, rope skipping, semi-squatting	
Khammassi *et al.* (2018)	Tunisia	12	10	17.4 ± 1.1	CON	no exercise	Weight, BMI, BPF
			10	17.8 ± 0.7	AE-HI	3 d/week; alternate 30 s of running at 100% of MAV and 30 s of active recovery at 50% of MAV; 50 min; running	
Dias *et al.* (2018)	Australia	12	34	11.8 ± 2.4	CON	no exercise	Weight, BMI, FM, BPF, FFM
			32	11.9 ± 2.4	AE-M	3 d/week; 60%–70% HR_max_; 44 min; moderate-intensity continuous training	
			33	12.4 ± 1.9	AE-HI	3 d/week; 85%–95% HR_max_; 40 min; high-intensity interval training	
Cvetković *et al.* (2018)	Serbia	12	14	11–13^a^	CON	no exercise	Weight, BMI, FM, BPF, FFM
			10		AE-HI	3 d/week; 60 min; 75%–90% HR_max_; Football training	
Wong *et al.* (2018)	Korea	12	15	15.3 ± 1.1	CON	no exercise	Weight, BMI, BPF
			15	15.2 ± 1.2	COM-M	3 d/week; 40%–70%HRR; 60 min; resistance band exercise and treadmill walking	
Martín-García *et al.* (2019)	Spain	12	28	11.1 ± 2.6	CON	no exercise	Weight, BMI, BPF, FM, FFM
			19	11.5 ± 2.4	AE-HI	2 d/week; 75.5% HR_max_; 90 min; aerobic games and some strength exercises	
Deldin et al. (2019)	United States	12	27	15.1 ± 1.7	AE-M	3 d/week; 50%–75%VO_2max_; 60 min; treadmills, ellipticals, or stationary bikes	Weight, BMI
			28	14.8 ± 1.5	RT-M	3 d/week; whole body resistance training (1–2 sets, 8–12 repetitions); 60 min; weight machines	
Kim *et al.* (2019)	Korea	12	24	15 ± 1	CON	no exercise	Weight, BMI, BPF
			24	15 ± 1	AE-M	5 d/week; 40%–70% HRR; 50 min; jump rope exercise	
Duft *et al.* (2020)	Brazil	12	19	14.7 ± 1.1	CON	no exercise	Weight, BMI, FM, BPF
			18	14.4 ± 1.4	COM-M	3 d/week; 60% VO_2max_; 60 min; resistance training and aerobic training	
Said *et al.* (2021)	Saudi Arabia	12	15	17.74 ± 1.42	CON	no exercise	Weight, BMI, BPF
			15		AE-M	4 d/week; 50%–70% HR_max_; 60 min; treadmills, ellipticals, or stationary bikes	
			16		RT-M	4 d/week; 50%–75% 1RM; 60 min; resistance training	
			15		COM-M	4 d/week; 50%–70% HR_max_ for aerobic exercise and 50%–75% 1RM for resistance training; 60 min; 50% of resistance training and 50% of the aerobic exercise	
Bouamra *et al.* (2022)	Tunisia	9	12	12.8 ± 0.9	AE-HI	3 d/week; 80%–110% VO_2max_; running; 60 min	Weight, BMI, BPF, FM, FFM
			12	12.7 ± 0.9	RT-HI	3 d/week; 3–4 sets × 10 RM; thera-band; 60 min	
			13	13.2 ± 0.9	COM-HI	3 d/week; 80%–110% VO_2max_ for aerobic exercise and 2 sets × 10 RM for resistance training; 60 min; 50% of resistance training and 50% of the aerobic exercise	
Cao *et al.* (2022)	China	12	13	11.0 ± 0.7	CON	no exercise	Weight, BMI, FM, BPF, FFM
			11	11.2 ± 0.7	AE-M	3 d/week; 60%–70% HR_max_; 30–40 min; moderate-intensity continuous training	
			12	11.4 ± 0.8	AE-HI	3 d/week; 80%–90% HR_max_; 30–40 min; high-intensity interval training	
Salus *et al.* (2022)	Estonia	12	14	13.7 ± 0.4	CON	no exercise	Weight, FM, BPF, FFM
			14	13.1 ± 0.3	AE-HI	3 d/week; 79.8%–81.7% HR_max_; 29–38 min; all-out sprint interval training	

BMI, body mass index; BFP, body fat percentage; FM, fat mass; FFM, fat-free mass; AE-M, moderate-intensity aerobic exercise; AE-HI, high-intensity aerobic exercise; RT-M, moderate-intensity resistance exercise; RT-HI, high-intensity resistance exercise; COM-M, moderate-intensity combined exercise; COM-HI, high-intensity combined exercise; CON, blank controls; VO_2max_, maximal oxygen uptake; reps, repetitions; RM, repetition maximum; HRmax, maximum heart rate; HRR, heart rate reserve.

^a^ Age range for entire study sample not mean (SD).

### 3.3 Risk of bias assessment results

The risk of bias was assessed for the included literature. According to the risk of bias assessment, 26 studies (81.3%) were classified as having “some concerns,” four studies (12.5%) were classified as “high risk,” and two studies (6.3%) were classified as “low risk.” The results of the risk of bias assessment for all studies are shown in Supplementary Table S3 and Supplementary Figure S1.

### 3.4 Network meta-analysis

#### 3.4.1 Network evidence

**FIGURE 2:**
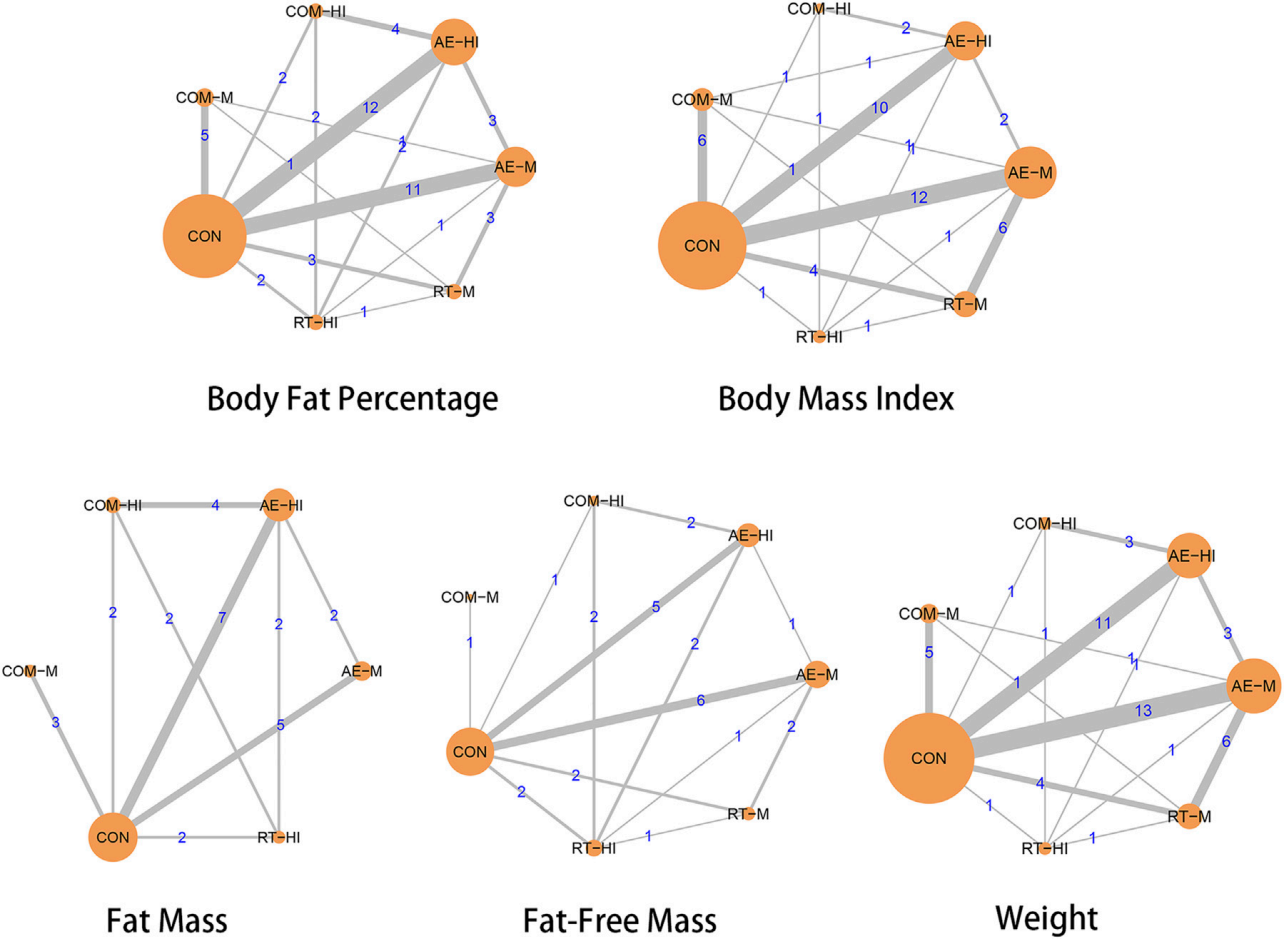
Network plot of included studies.

The network meta-analysis included five body composition indicators: body fat percentage, BMI, fat mass, fat-free mass, and weight. Figure 2 shows the network plots of different intensity exercise modalities with the five body composition indicators. In each intervention’s relationship, the circles represent different interventions, with their size indicating the sample size included in that intervention. The lines represent the existence of direct comparisons between interventions, and the thickness represents the number of studies between two interventions. Both global and local inconsistency test analyses showed significant *p* > 0.05 for each outcome indicator, indicating that the consistency assumption was met (as shown in Supplementary Table S5, S6).

#### 3.4.2 Body fat percentage

Twenty-eight studies compared the effects of interventions with different intensity exercise modalities on body fat percentage. The results of the pairwise and network meta-analysis are shown in Table 3. All exercise interventions, except for RT-M (*p* < 0.05), improved body fat percentage in obese children and adolescents when compared with the blank control group. Direct comparisons between the interventions showed that high-intensity resistance exercise improved body fat percentage better than high-intensity aerobic exercise [mean difference in % = −0.49, 95% CI (−0.82, −0.16)]. Forest plots for pairwise comparative meta-analysis are available in the Supplementary Figure S4.

**TABLE 3:** Pairwise and network meta-analysis of the effectiveness of interventions on body fat percentage.

	COM-M	COM-HI	AE-M	RT-HI	AE-HI	RT-M	CON
COM-M	COM-M	NA	−0.11 (−0.77, 0.55) N = 1, *I* ^*2*^ = NA	NA	NA	−0.82 (−1.65, −0.01) N = 1, *I* ^*2*^ = NA	**−2.37 (−4.17, −0.57) N = 4, *I*** ^***2***^ **= 92%**
COM-HI	−0.33 (−2.33,1.66)	COM-HI	NA	−0.03 (−0.81, 0.74) N = 2, *I* ^*2*^ = 25%	−0.20 (−0.90, 0.49) N = 3, *I* ^*2*^ = 41%	N = 2, *I* ^*2*^ = 25%	**−1.97 (−3.88, −0.06) N = 2, *I*** ^***2***^ **= 87%**
AE-M	−0.43 (−1.90,1.04)	−0.10 (−1.77,1.57)	AE-M	2.69 (−0.15, 5.53) N = 1, *I* ^*2*^ = NA	−0.04 (−1.09, 1.02) N = 3, *I* ^*2*^ = 59%	0.58 (−0.99, 2.16) N = 3, *I* ^*2*^ = 85%	**−2.36 (−2.18, −1.53) N = 11, *I*** ^***2***^ **= 93%**
RT-HI	−0.58 (−2.12,0.97)	−0.25 (−1.74,1.25)	−0.14 (−1.21,0.92)	RT-HI	**−0.49 (−0.82, -0.16) N = 2, *I*** ^***2***^ **= 0%**	1.61 (−1.44, 4.66) N = 1, *I* ^*2*^ = NA	**−1.55 (−2.13, −0.97) N = 2, *I*** ^***2***^ **= 67%**
AE-HI	−0.51 (−2.44,1.43)	−0.17 (−1.92,1.57)	−0.07 (−1.66,1.51)	−0.07 (−1.57,1.43)	AE-HI	NA	**−2.03 (−2.96, −1.11) N = 12, *I*** ^***2***^ **=95%**
RT-M	−0.73 (−2.49,1.04)	−0.39 (−2.40,1.61)	−0.29 (−1.71,1.12)	−0.22 (−2.11,1.67)	−0.15 (−1.71,1.41)	RT-M	−0.71 (−1.97, 0.55) N = 3, *I* ^*2*^ = 85%
CON	**−2.52 (−3.83, −1.20)**	**−2.18 (−3.70, −0.67)**	**−2.08 (−2.88, −1.28)**	**−2.01 (−3.45, −0.57)**	**−1.94 (−2.77, −1.11)**	**−1.79 (−3.15, −0.43)**	CON

The gray area represents the results of the network meta-analysis, and the white area represents the results of the pairwise meta-analysis. The bold values represent statistical significance at a level of p < 0.05. Estimates are shown as columns versus rows for network meta-analysis and rows versus columns for pairwise meta-analysis. The results are presented as mean difference (MD). N, number of studies in the comparison; NA, not available; AE-M, moderate-intensity aerobic exercise; AE-HI, high-intensity aerobic exercise; RT-M, moderate-intensity resistance exercise; RT-HI, high-intensity resistance exercise; COM-M, moderate-intensity combined exercise; COM-HI, high-intensity combined exercise; CON, blank controls.

The results of the network meta-analysis showed that COM-M [mean difference in % = −2.52, 95% CI (−3.83, −1.20)], COM-HI [mean difference in % = −2.18, 95% CI (−3.70, −0.67)], AE-M [mean difference in % = -2.08, 95% CI (−2.88, −1.28)], RT-HI [mean difference in % = -2.01, 95% CI (−3.45, −0.57)], AE-HI [mean difference in % = −1.94, 95% CI (−2.77, −1.11)], and RT-M [mean difference in % = −1.79, 95% CI (−3.15, −0.43)] were significantly superior to the control group (*p* < 0.05). The contribution matrix of direct and indirect evidence is shown in the Supplementary Figure S7; Table 3 presents the estimates for the comparison of all interventions. The SUCRA values for the different interventions are shown in Table 4, with COM-M being the most effective intervention in reducing the percentage of body fat (76.0%), followed by COM-HI (63.1%), AE-M (58.7%), RT-HI (55.5%), AE-HI (50.6%), and RT-M (45.9%) in the ranking of the other interventions.

**TABLE 4:** Ranking of each intervention on body fat percentage.

	SUCRA, %	Rank*, %	Mean rank
CON	0.2	0.0	7.0
AE-M	58.7	7.5	3.5
AE-HI	50.6	4.3	4.0
RT-M	45.9	8.5	4.2
RT-HI	55.5	14.4	3.7
COM-M	76.0	41.9	2.4
COM-HI	63.1	23.4	3.2

SUCRA, surface under the cumulative ranking curve. *Rank probability ranges from 0 to 100, where higher value indicates best intervention and lower value indicates worst intervention. AE-M, moderate-intensity aerobic exercise; AE-HI, high-intensity aerobic exercise; RT-M, moderate-intensity resistance exercise; RT-HI, high-intensity resistance exercise; COM-M, moderate-intensity combined exercise; COM-HI, high-intensity combined exercise; CON, blank controls.

#### 3.4.3 Body mass index

Thirty studies compared the effects of interventions using different exercise modalities of varying intensities on BMI. The results of the pairwise and network meta-analysis are shown in Table 5. Compared to blank controls, COM-HI [mean difference in kg/m^2^ = −1.90, 95% CI (−3.22, −0.56)], AE-M [mean difference in kg/m^2^ = −1.53, 95% CI (−2.56, −1.09)], COM-M [mean difference in kg/m^2^ = −1.34, 95% CI (−2.27, −0.42)], and AE-HI [mean difference in kg/m^2^ = −1.29, 95% CI (−2.01, −0.58)] showed significant improvement in BMI (*p* < 0.05). Direct comparisons between interventions revealed that COM-M was superior to RT-M [mean difference in kg/m^2^ = −0.76, 95% CI (−0.46, −0.06)], and COM-HI was superior to RT-HI [mean difference in kg/m^2^ = −1.20, 95% CI (−2.26, −0.14)] in terms of BMI improvement. Forest plots for pairwise comparative meta-analysis are available in the Supplementary Figure S4.

**TABLE 5:** Pairwise and network meta-analysis of the effectiveness of interventions on body mass index.

	COM-HI	AE-M	COM-M	AE-HI	RT-M	RT-HI	CON
COM-HI	COM-HI	NA	NA	−0.36 (−2.25, 1.54) N = 2, *I* ^*2*^ = 81%	NA	**−1.20 (−2.26, −0.14) N = 1, *I*** ^***2***^ **= NA**	**−1.90 (−3.22, −0.58) N = 1, *I*** ^***2***^ **= NA**
AE-M	−0.16 (−1.86, 1.55)	AE-M	0.36 (−0.29, 1.01) N = 1, *I* ^*2*^ = NA	−0.72 (−1.45, 0.01) N = 2, *I* ^*2*^ = 0%	−0.13 (−0.43, 0.18) N = 6, *I* ^*2*^ = 78%	−0.84 (−2.41, 0.73) N = 1, *I* ^*2*^ = NA	**−1.53 (−2.06, −1.00) N = 12, *I*** ^***2***^ **= 91%**
COM-M	−0.22 (−2.06, 1.63)	−0.06 (−1.13, 1.00)	COM-M	0.81 (−0.53, 2.15) N = 1, *I* ^*2*^ = NA	**−0.76 (−1.46, −0.06) N = 1, *I*** ^***2***^ **= NA**	NA	**−1.34 (−2.27, −0.42) N = 6, *I*** ^***2***^ **= 86%**
AE-HI	−0.46 (−2.06, 1.13)	−0.31 (−1.21, 0.59)	−0.25 (−1.38, 0.88)	AE-HI	NA	0.10 (−0.86, 1.06) N = 1, *I* ^*2*^ = NA	**1.29 (−2.01, −0.58) N = 10, *I*** ^***2***^ **= 79%**
RT-M	−0.73 (−2.54, 1.08)	−0.57 (−1.41, 0.26)	−0.52 (−1.71, 0.68)	−0.27 (−1.36, 0.83)	RT-M	−1.21 (−3.00, 0.58) N = 1, *I* ^*2*^ = NA	−0.30 (−0.89, 0.28) N = 4, *I* ^*2*^ = 85%
RT-HI	−1.21 (−3.03, 0.60)	−1.06 (−2.45, 0.33)	−1.00 (−2.59, 0.60)	−0.75 (−2.15, 0.65)	−0.48 (−1.97, 1.01)	RT-HI	−0.10 (−0.40, 0.20) N = 1, *I* ^*2*^ = NA
CON	**−1.65 (−3.27, −0.02)**	**−1.49 (−2.12, −0.86)**	**−1.43 (−2.35, −0.51)**	**−1.18 (−1.93, −0.44)**	**−0.91 (−1.78, −0.05)**	−0.43 (−1.76, 0.90)	CON

The gray area represents the results of the network meta-analysis, and the white area represents the results of the pairwise meta-analysis. The bold values represent statistical significance at a level of p < 0.05. Estimates are shown as columns versus rows for network meta-analysis and rows versus columns for pairwise meta-analysis. The results are presented as mean difference (MD). N, number of studies in the comparison; NA, not available; AE-M, moderate-intensity aerobic exercise; AE-HI, high-intensity aerobic exercise; RT-M, moderate-intensity resistance exercise; RT-HI, high-intensity resistance exercise; COM-M, moderate-intensity combined exercise; COM-HI, high-intensity combined exercise; CON, blank controls.

The results of the network meta-analysis showed that compared to controls, COM-HI [mean difference in kg/m^2^ = −1.65, 95% CI (−3.27, −0.02)], AE-M [mean difference in kg/m^2^ = −1.49, 95% CI (−2.12, −0.86)], COM-M [mean difference in kg/m^2^ = −1.43, 95% CI (−2.35, −0.51)], AE-HI [mean difference in kg/m^2^ = −1.18, 95% CI (−1.93, −0.44)], RT-M [mean difference in kg/m^2^ = −0.91, 95% CI (−1.78, −0.05)], and RT-HI [mean difference in kg/m^2^ = −0.43, 95% CI (−1.76, −0.90)] were more effective. The contribution matrix of direct and indirect evidence is presented in the Supplementary Figure S7. The SUCRA values for each intervention are shown in Table 6, with COM-HI having the highest effectiveness (76.1%), followed by AE-M (75.9%), COM-M (70.6%), AE-HI (57.1%), RT-M (41.9%), and RT-HI (23.7%).

**TABLE 6:** Ranking of each intervention on body mass index.

	SUCRA, %	Rank*, %	Mean rank
CON	5.3	0.0	6.7
AE-M	75.9	21.7	2.4
AE-HI	56.6	5.0	3.6
RT-M	42.6	2.5	4.4
RT-HI	23.4	1.3	5.6
COM-M	70.2	22.7	2.8
COM-HI	76.1	46.7	2.4

SUCRA, surface under the cumulative ranking curve. *Rank probability ranges from 0 to 100, where higher value indicates best intervention and lower value indicates worst intervention. AE-M, moderate-intensity aerobic exercise; AE-HI, high-intensity aerobic exercise; RT-M, moderate-intensity resistance exercise; RT-HI, high-intensity resistance exercise; COM-M, moderate-intensity combined exercise; COM-HI, high-intensity combined exercise; CON, blank controls.

#### 3.4.4 Fat mass

Fifteen studies compared the effects of exercise interventions with different intensity modalities on fat mass. The results of pairwise and network meta-analysis are presented in Table 7. Pairwise meta-analysis revealed that COM-HI [mean difference in kg = −2.40, 95% CI (−3.87, −0.92)], AE-HI [mean difference in kg = −2.55, 95% CI (−3.77, −1.34)], AE-M [mean difference in kg = −2.38, 95% CI (−4.33, −0.42)], and RT-HI [mean difference in kg = −1.47, 95% CI (−2.40, −0.90)] were more effective at reducing fat mass than blank controls (*p* < 0.05). Forest plots for the pairwise meta-analysis are provided in the Supplementary Figure S4. Network meta-analysis results showed that AE-M [mean difference in kg = −2.31, 95% CI (−3.83, −0.79)], AE-HI [mean difference in kg = −2.25, 95% CI (−3.51, −1.00)], COM-M [mean difference in kg = −2.87, 95% CI (−4.97, −0.76)], and COM-HI [mean difference in kg = −2.87, 95% CI (−4.84, −0.91)] had significantly better effects than blank controls (*p* < 0.05). RT-HI [mean difference in kg = −1.56, 95% CI (−3.40, 0.28)] did not show statistical significance (*p* > 0.05). Table 8 provide the SUCRA values for each intervention, with the COM-HI intervention being the most effective (76.0%), and the other interventions ranked by cumulative probability as COM-M (73.4%), AE-M (58.5%), AE-HI (55.7%), and RT-HI (35.2%).

**TABLE 7:** Pairwise and network meta-analysis of the effectiveness of each intervention on fat mass.

	COM-HI	COM-M	AE-M	AE-HI	RT-HI	CON
COM-HI	COM-HI	NA	NA	−0.51 (−1.28, 0.26) N = 3, *I* ^*2*^ = 19%	−1.16 (−3.35, 1.02) N = 2, *I* ^*2*^ = 74%	**−2.40 (−3.87, −0.92) N = 2, *I*** ^***2***^ **-40%**
COM-M	−0.01 (−2.89, 2.88)	COM-M	NA	NA	NA	−2.78 (−5.68, 0.12) N = 3, *I* ^*2*^ = 85%
AE-M	−0.56 (−2.96, 1.84)	−0.55 (−3.15, 2.04)	AE-M	−0.86 (−2.20, 0.48) N = 2, *I* ^*2*^ = 14%	NA	**−2.38 (−4.33, −0.42) N = 5, *I*** ^***2***^ **= 92%**
AE-HI	−0.62 (−2.53, 1.29)	−0.61 (−3.06, 1.84)	−0.06 (−1.84, 1.72)	AE-HI	0.09 (−0.10, 0.29) N = 2, *I* ^*2*^ = 0%	**−2.55 (−3.77, −1.34) N = 7, *I*** ^***2***^ **= 91%**
RT-HI	−1.31 (−3.47, 0.84)	−1.30 (−4.10, 1.49)	−0.75 (−3.07, 1.57)	−0.69 (−2.58, 1.19)	RT-HI	**−1.47 (−2.40, −0.90) N = 2, *I*** ^***2***^ **= 59%**
CON	**−2.87 (−4.84, −0.91)**	**−2.87 (−4.97, −0.76)**	**−2.31 (−3.83, −0.79)**	**−2.25 (−3.51, −1.00)**	−1.56 (−3.40, 0.28)	CON

The gray area represents the results of the network meta-analysis, and the white area represents the results of the pairwise meta-analysis. The bold values represent statistical significance at a level of p < 0.05. Estimates are shown as columns versus rows for network meta-analysis and rows versus columns for pairwise meta-analysis. The results are presented as mean difference (MD). N, number of studies in the comparison; NA, not available; AE-M, moderate-intensity aerobic exercise; AE-HI, high-intensity aerobic exercise; RT-M, moderate-intensity resistance exercise; RT-HI, high-intensity resistance exercise; COM-M, moderate-intensity combined exercise; COM-HI, high-intensity combined exercise; CON, blank controls.

**TABLE 8:** Ranking of each intervention on fat mass.

	SUCRA, %	Rank*, %	Mean rank
CON	1.1	0.0	5.9
AE-M	58.5	13.1	3.1
AE-HI	55.7	6.0	3.2
RT-HI	35.2	2.6	4.2
COM-M	73.4	40.3	2.3
COM-HI	76.0	38.0	2.2

SUCRA, surface under the cumulative ranking curve. *Rank probability ranges from 0 to 100, where higher value indicates best intervention and lower value indicates worst intervention. AE-M, moderate-intensity aerobic exercise; AE-HI, high-intensity aerobic exercise; RT-M, moderate-intensity resistance exercise; RT-HI, high-intensity resistance exercise; COM-M, moderate-intensity combined exercise; COM-HI, high-intensity combined exercise; CON, blank controls.

#### 3.4.5 Fat-free mass

Fifteen studies examined the effect of different intensity exercise modalities on fat-free mass. The results of pairwise and network meta-analysis are presented in Table 9. Pairwise meta-analysis results showed that RT-M significantly increased fat-free mass compared to blank controls [mean difference in kg = 0.70, 95% CI (0.46, 0.94)]. Direct comparisons between interventions showed that RT-HI intervention was superior to AE-HI [mean difference in kg = 0.69, 95% CI (0.47, 0.91)] and RT-M was superior to AE-M [mean difference in kg = 0.88, 95% CI (0.65, 1.11)]. Pairwise meta-analysis forest plots are available in the Supplementary Figure S4. The results of the network meta-analysis showed that RT-HI [mean difference in kg = 1.10, 95% CI (0.22, 1.99)] was significantly better than the blank controls (*p* < 0.05). Table 10 present the SUCRA results, with RT-HI showing the best effect (84.0%), and the cumulative probabilities of the other interventions ranked as RT-M (63.9%), AE-HI (61.0%), COM-HI (51.7%), COM-M (39.5%), and AE-M (31.3%).

**TABLE 9:** Pairwise and network meta-analysis of the effectiveness of each intervention on fat-free mass.

	RT-HI	RT-M	AE-HI	COM-HI	COM-M	AE-M	CON
RT-HI	RT-HI	1.40 (−1.74, 4.54) N = 1, *I* ^*2*^ = NA	**0.69 (0.47, 0.91) N = 2, *I*** ^***2***^ **= 0%**	0.44 (−0.44, 1.31) N = 2, *I* ^*2*^ = 22%	NA	1.64 (−2.98, 6.26) N = 1, *I* ^*2*^ = NA	0.97 (−0.40, 2.34) N = 2, *I* ^*2*^ = 94%
RT-M	0.40 (−0.83, 1.63)	RT-M	NA	NA	NA	**0.88 (0.65, 1.11) N = 2, *I*** ^***2***^ **= 0%**	**0.70 (0.46, 0.94) N = 2, *I*** ^***2***^ **= 0%**
AE-HI	0.45 (−0.52, 1.43)	0.05 (−1.09, 1.19)	AE-HI	NA	NA	0.70 (−1.53, 2.93) N = 1, *I* ^*2*^ = NA	0.63 (−0.40, 1.66) N = 5, *I* ^*2*^ = 95%
COM-HI	0.59 (−0.57, 1.75)	0.19 (−1.25, 1.63)	0.14 (−0.99, 1.27)	COM-HI	0.10 (−0.12, 0.32) N = 2, I^2^ = 0%	NA	**−0.30 (−0.52, −0.08) N = 1, *I*** ^***2***^ **= NA**
COM-M	1.10 (−2.70, 4.91)	0.70 (−3.11, 4.51)	0.65 (−3.12, 4.42)	0.51 (−3.36, 4.38)	COM-M	NA	0.00 (−3.45, 3.45) N = 1, *I* ^*2*^ = NA
AE-M	0.94 (−0.17, 2.05)	0.54 (−0.48, 1.56)	0.49 (−0.48, 1.45)	0.35 (−0.96, 1.65)	−0.16 (−3.93, 3.61)	AE-M	−0.02 (−0.30, 0.26) N = 6, I^2^ = 25%
CON	**1.10 (0.22, 1.99)**	0.70 (−0.21, 1.61)	0.65 (−0.06, 1.36)	0.51 (−0.62, 1.64)	−0.00 (−3.70, 3.70)	0.16 (−0.54, 0.87)	CON

The gray area represents the results of the network meta-analysis, and the white area represents the results of the pairwise meta-analysis. The bold values represent statistical significance at a level of p < 0.05. Estimates are shown as columns versus rows for network meta-analysis and rows versus columns for pairwise meta-analysis. The results are presented as mean difference (MD). N, number of studies in the comparison; NA, not available; AE-M, moderate-intensity aerobic exercise; AE-HI, high-intensity aerobic exercise; RT-M, moderate-intensity resistance exercise; RT-HI, high-intensity resistance exercise; COM-M, moderate-intensity combined exercise; COM-HI, high-intensity combined exercise; CON, blank controls.

**TABLE 10:** Ranking of each intervention on fat-free mass.

	SUCRA, %	Rank*, %	Mean rank
CON	18.6	0.0	5.9
AE-M	31.3	0.8	5.1
AE-HI	61.0	6.7	3.3
RT-M	63.9	15.0	3.2
RT-HI	84.0	44.0	2.0
COM-M	39.5	25.7	4.6
COM-HI	51.7	7.8	3.9

SUCRA, surface under the cumulative ranking curve. *Rank probability ranges from 0 to 100, where higher value indicates best intervention and lower value indicates worst intervention. AE-M, moderate-intensity aerobic exercise; AE-HI, high-intensity aerobic exercise; RT-M, moderate-intensity resistance exercise; RT-HI, high-intensity resistance exercise; COM-M, moderate-intensity combined exercise; COM-HI, high-intensity combined exercise; CON, blank controls.

#### 3.4.6 Weight

In this study, thirty-one different research works compared the effects of various exercise modalities with different intensities on weight loss. The results of pairwise and network meta-analysis are presented in Table 11. The results of the pairwise meta-analysis revealed that COM-M [mean difference in kg = −4.49, 95% CI (−6.24, −2.73)], AE-HI [mean difference in kg = −2.33, 95% CI (−3.01, −1.46)], and AE-M [mean difference in kg = −2.03, 95% CI (−2.58, −1.47)] were more effective in weight loss than the blank controls. Moreover, the direct comparisons of the interventions revealed that COM-M was superior to RT-M [mean difference in kg = -1.82, 95% CI (−5.09, −1.45)] and AE-HI was superior to AE-M [mean difference in kg = −0.68, 95% CI (−1.74, −0.37)]. The pairwise meta-analysis forest plots are presented in the Supplementary Figure S4.

**TABLE 11:** Pairwise and network meta-analysis of the effectiveness of each intervention on weight.

	COM-M	COM-HI	AE-HI	AE-M	RT-M	RT-HI	CON
COM-M	COM-M	NA	NA	−1.32 (−1.86, 4.50) N = 1, *I* ^*2*^ = NA	**−1.82 (−5.09, −1.45) N = 1, *I*** ^***2***^ **= NA**	NA	**−4.49 (−6.24, −2.73) N = 5, *I*** ^***2***^ **= 67%**
COM-HI	−1.46 (−4.76, 1.83)	COM-HI	−1.00 (−4.87, 2.86) N = 3, *I* ^*2*^ = 49%	NA	NA	−3.10 (−7.46, 1.26) N = 1, *I* ^*2*^ = NA	−2.81 (−7.58, 1.96) N = 1, *I* ^*2*^ = NA
AE-HI	−2.29 (−3.94, −0.65)	−0.83 (−3.79, 2.13)	AE-HI	**−0.68 (−1.74, -0.37) N = 3, *I*** ^***2***^ **= 27%**	NA	0.00 (-4.02, 4.02) N = 1, *I* ^*2*^ = NA	**−2.33 (−3.01, −1.46) N = 11, *I*** ^***2***^ **= 60%**
AE-M	−2.55 (−4.08, −1.01)	−1.08 (−4.13, 1.97)	−0.25 (−1.30, 0.79)	AE-M	−0.42 (−1.26, 0.42) N = 6, *I* ^*2*^ = 78%	−2.05 (−6.69, 2.59) N = 1, *I* ^*2*^ = NA	**−2.03 (−2.58, −1.47) N = 13, *I*** ^***2***^ **= 57%**
RT--M	−3.17 (−4.81, −1.53)	−1.71 (−4.84, 1.42)	−0.88 (−2.15, 0.39)	−0.63 (−1.55, 0.30)	RTM	−1.66 (−6.82, 3.50) N = 1, *I* ^*2*^ = NA	−1.44 (−3.35, 0.47) N = 4, *I* ^*2*^ = 91%
RT-HI	−4.22 (−6.47, −1.96)	−2.75 (−5.99, 0.49)	−1.92 (−3.85, −0.00)	−1.67 (−3.55, 0.21)	−1.04 (−3.03, 0.94)	RT-HI	−0.20 (−1.04, 0.64) N = 1, *I* ^*2*^ = NA
CON	**−4.58 (−5.94, −3.22)**	**−3.11 (−6.11, −0.12)**	**−2.29 (−3.19, −1.39)**	**−2.03 (−2.76, −1.31)**	**−1.41 (−2.38, −0.44)**	−0.36 (−2.15, 1.43)	CON

The gray area represents the results of the network meta-analysis, and the white area represents the results of the pairwise meta-analysis. The bold values represent statistical significance at a level of p < 0.05. Estimates are shown as columns versus rows for network meta-analysis and rows versus columns for pairwise meta-analysis. The results are presented as mean difference (MD). N, number of studies in the comparison; NA, not available; AE-M, moderate-intensity aerobic exercise; AE-HI, high-intensity aerobic exercise; RT-M, moderate-intensity resistance exercise; RT-HI, high-intensity resistance exercise; COM-M, moderate-intensity combined exercise; COM-HI, high-intensity combined exercise; CON, blank controls.

Furthermore, the results of the network meta-analysis indicated that AE-M [mean difference in kg = −2.03, 95%CI (−2.76, −1.31)], AE-HI [mean difference in kg = −2.29, 95%CI(-3.19, −1.39)], RT-M [mean difference in kg = −1.41, 95%CI(−2.38, −0.44)], COM-M [mean difference in kg = −4.58, 95% CI (−5.94, −3.22)], and COM-HI [mean difference in kg = −3.11, 95% CI (−6.11, −0.12)] were significantly more effective in weight loss than the blank control (p < 0.05). However, COM-HI [mean difference in kg = −0.36, 95% CI (−2.15, 1.43)] did not show any statistically significant difference (*p* > 0.05). The cumulative probability ranking of each intervention is presented in Table 12. The results showed that the COM-M intervention was the most effective (96.9%), followed by COM-HI (73.4%), AE-HI (64.5%), AE-M (57.5%), RT-M (36.1%), and RT-HI (15.5%).

**TABLE 12:** Ranking of each intervention on weight.

	SUCRA, %	Rank*, %	Mean rank
CON	6.1	0.0	6.6
AE-M	57.5	0.0	3.6
AE-HI	64.5	0.1	3.1
RT-M	36.1	0.0	4.8
RT-HI	15.5	0.0	6.1
COM-M	96.9	81.9	1.2
COM-HI	73.4	18.0	2.6

SUCRA, surface under the cumulative ranking curve. *Rank probability ranges from 0 to 100, where higher value indicates best intervention and lower value indicates worst intervention. AE-M, moderate-intensity aerobic exercise; AE-HI, high-intensity aerobic exercise; RT-M, moderate-intensity resistance exercise; RT-HI, high-intensity resistance exercise; COM-M, moderate-intensity combined exercise; COM-HI, high-intensity combined exercise; CON, blank controls.

### 3.5 Publication bias

To test the publication bias, “corrected-comparison” funnel plots were drawn for body fat percentage, BMI, body fat mass, fat-free mass, and weight. The results showed poor symmetry on both sides of the funnel plots, suggesting the presence of publication bias or small sample effect in this study, as shown in the Supplementary Figure S9.

## 4 Discussion

The Network meta-analysis results showed that combined exercise (moderate and high intensity) was the most effective in reducing body fat percentage, BMI, fat mass, and body weight compared to the blank controls. Among the six type interventions, high-intensity resistance exercise was the less effective in reducing body weight, BMI, and fat mass but the most effective in increasing fat-free mass. These findings are consistent with the results of pairwise meta-analyses. Based on the effect size and Surface Under the Cumulative Ranking values (SUCRA), combined exercise (moderate and high intensity) had the highest likelihood of being the optimal measure of the combined effect of current interventions on body composition in overweight and obese children and adolescents. Overall, in terms of fat reduction, the effectiveness of all exercise models was ordered as combined exercise—aerobic exercise—resistance exercise.

Weight and body mass index (BMI) are overall indicators of overweight and obesity and are the primary targets for treating obesity. In the current meta-analysis, all exercise interventions were effective in reducing body weight and BMI, except for high-intensity resistance exercise. Meanwhile, it was found that the effectiveness of exercise interventions depends mainly on the type of exercise. Specifically, combined exercise, whether moderate or high intensity, was more effective than single aerobic or resistance exercise, and aerobic exercise was more effective than resistance exercise. These findings are consistent with the results of previous studies (Willis et al., 2012; O’Donoghue et al., 2021).

The mechanisms of combined exercise suggest that aerobic exercise decreases plasma insulin levels and increases the secretion of glucagon, catecholamines, and epinephrine, prompting an increase in the activity of rate-limiting enzymes of the fat hydrolysis process. This accelerates fat hydrolysis and promotes lipolysis (Zouhal et al., 2013; Nakhostin-Roohi and Havaskar, 2018). Resistance exercise reduces fat mass mainly by activating the musculoskeletal system, increasing de-fatted body weight, and increasing resting metabolic rate (Dinas et al., 2014). Thus, integrating aerobic and resistance exercise stimuli increases exercise energy expenditure and resting metabolic rate, inducing a cumulative effect of both exercise modalities (Gäbler et al., 2018). Based on these theories, combined training may be the best exercise modality to reduce body weight and BMI.

Increases in fat mass and body fat percentage, and decreases in fat-free mass, are important features of obesity in children and adolescents. In the current study, the effects of different intensity exercise modalities varied for different outcome indicators. Combined exercise of two intensities was the most effective intervention for fat-related outcome indicators, such as fat mass and body fat percentage. This is consistent with the findings of George and Schwingshackl et al. (2013); George et al., 2017). For individuals specifically seeking to reduce total body fat volume, the best option is a form of exercise that produces the greatest total energy expenditure (Wilhelm and Pinto, 2019). However, individuals may experience fatigue when consistently performing a single exercise modality for long periods. Therefore, combined training can be an alternative to increase total energy expenditure by additionally increasing training volume and diversifying training modalities (Donges et al., 2013).

Furthermore, combined training may reduce the amount of fat not only directly but also further reduce the percentage of body fat by increasing the amount of fat-free mass. The study also found that resistance exercise was less effective than other exercise modalities in reducing fat but was optimally effective in increasing fat-free mass. These findings are consistent with previous studies (Wilson et al., 2012; Schranz et al., 2013; Grgic et al., 2019). This may be because resistance exercise can selectively hypertrophy type II muscle fibers, thereby increasing total lean body mass (Fry, 2004; Farup et al., 2012). In contrast, aerobic exercise leaves type II muscle fibers unchanged or reduces muscle fibers (Edström and Ekblom, 1972).

This study may have several biases that could affect its validity. First, the total volume of exercise could be a crucial factor in the effectiveness of the intervention. Evidence suggests that combined aerobic and resistance exercise requires twice the training volume of single aerobic or resistance exercise, resulting in greater energy expenditure and fat reduction (Martins et al., 2010; Willis et al., 2012). Additionally, Rhea et al. (2003) found that combining resistance and aerobic exercise led to higher total training volumes and superior improvements in body composition compared to single-mode exercise (Rhea et al., 2003). Second, the frequency and duration of exercise should also be carefully considered. Although most studies in this review used a three-times-per-week intervention, some studies used more or fewer sessions per week or varied the duration of each session. Such variations could lead to an unbalanced intervention protocol and introduce bias to the results. Therefore, it is important to standardize these variables to minimize their confounding effects on the outcomes. However, the study’s methodological limitations precluded an in-depth analysis of the effects of exercise frequency and single exercise session duration on the intervention’s efficacy.

To the best of our knowledge, this study is the first to focus on the effects of different types of exercise intensity on changes in body composition in overweight and obese children and adolescents using a network meta-analysis. This study can provide a reference for choosing the optimal exercise program for overweight and obese children and adolescents. Additionally, this study contains a relatively comprehensive set of body composition indicators, including body fat percentage, BMI, body fat mass, fat-free mass, and weight. It compares the effects of different exercise patterns on different indicators, providing instructors, researchers, and policymakers with useful and up-to-date information on the effects of different exercise patterns on different body components.

However, there are several limitations to this study. First, despite a comprehensive search of the published literature, some studies that used keywords other than those searched for in this paper may not have been included, resulting in selection bias. Second, the study currently lacks evidence for interventions such as low-intensity aerobic exercise, resistance exercise, and combined exercise, limiting the study’s comprehensiveness. Third, as other components of the exercise intervention, such as time and frequency, were not considered in this study, this may have influenced the study’s findings. Fourth, the included literature primarily focuses on aerobic exercise, with fewer studies on resistance and combined exercise interventions, which limits the number of direct comparisons between different exercise interventions and thus lowers the reliability of the evidence. Fifth, according to the CINeMA assessment, most evidence was rated as low or very low in the different comparisons. Thus, this may weaken the strength of the current findings.

## 5 Conclusion

In conclusion, combined exercise, whether of moderate or high intensity, has been found to be the most effective intervention for promoting weight loss and improving BMI, fat mass, and body fat percentage in overweight and obese children and adolescents. Additionally, high-intensity resistance exercise has been found to be the best intervention for increasing fat-free mass. These findings are significant for developing effective exercise interventions for managing obesity in children and adolescents. However, while this study provides important insights into the effects of different types of exercise intensity on changes in body composition in overweight and obese children and adolescents, there are several limitations that need to be addressed in future research to improve the comprehensiveness and reliability of the evidence.

## Data Availability

The original contributions presented in the study are included in the article/Supplementary Material, further inquiries can be directed to the corresponding author.

## References

[B2] Alberga A. S. Farnesi B. C. Lafleche A. Legault L. Komorowski J. (2013). The effects of resistance exercise training on body composition and strength in obese prepubertal children. Physician Sports Med. 41 (3), 103–109. 10.3810/psm.2013.09.2028 24113708

[B3] Bouamra M. Zouhal H. Ratel S. Makhlouf I. Bezrati I. Chtara M. (2022). Concurrent training promotes greater gains on body composition and components of physical fitness than single-mode training (endurance or resistance) in youth with obesity. Front. Physiology 13, 869063. 10.3389/fphys.2022.869063 PMC9164296 35669575

[B4] Cao M. Yucheng T. Shu L. Yu Z. (2022). Effects of school-based high-intensity interval training on body composition, cardiorespiratory fitness and cardiometabolic markers in adolescent boys with obesity: A randomized controlled trial. BMC Pediatr. 22 (1), 112. 10.1186/s12887-021-03079-z 35232402 PMC8886768

[B5] Chaimani A. Caldwell D. M. Li T. Higgins J. P. T. Salanti G. (2017). Additional considerations are required when preparing a protocol for a systematic review with multiple interventions. J. Clin. Epidemiol. 83, 65–74. 10.1016/j.jclinepi.2016.11.015 28088593

[B6] Cvetković N. Stojanović E. Stojiljković N. Nikolić D. Scanlan A. T. Milanović Z. (2018). Exercise training in overweight and obese children: Recreational football and high-intensity interval training provide similar benefits to physical fitness. Scand. J. Med. Sci. Sports 28 (Suppl. 1), 18–32. 10.1111/sms.13241 29979479

[B7] Daniels S. R. Arnett D. K. Eckel R. H. Gidding S. S. Hayman L. L. Kumanyika S. (2005). Overweight in children and adolescents: Pathophysiology, consequences, prevention, and treatment. Circulation 111 (15), 1999–2012. 10.1161/01.CIR.0000161369.71722.10 15837955

[B8] Deldin A. Kuk J. L. Lee S. (2019). Influence of sex on the changes in regional fat and skeletal muscle mass in response to exercise training in adolescents with obesity. Child. Obes. 15 (3), 216–222. 10.1089/chi.2018.0329 30694699 PMC6442263

[B9] Dias K. A. Ingul C. B. Tjønna A. E. Keating S. E. Gomersall S. R. Follestad T. (2018). Effect of high-intensity interval training on fitness, fat mass and cardiometabolic biomarkers in children with obesity: A randomised controlled trial. Sports Med. 48 (3), 733–746. 10.1007/s40279-017-0777-0 28853029

[B10] Dinas P. C. Markati A. S. Carrillo A. E. (2014). Exercise-Induced Biological and Psychological Changes in Overweight and Obese Individuals: A Review of Recent Evidence. ISRN Physiol. 1–12. 10.1155/2014/964627

[B11] Donges C. E. Duffield R. Guelfi K. J. Smith G. C. Adams D. R. Edge J. A. (2013). Comparative effects of single-mode vs. duration-matched concurrent exercise training on body composition, low-grade inflammation, and glucose regulation in sedentary, overweight, middle-aged men. Appl. Physiology, Nutr. Metabolism 38 (7), 779–788. 10.1139/apnm-2012-0443 23980737

[B12] Duft R. G. Castro A. Bonfante I. L. P. Lopes W. A. da Silva L. R. Chacon-Mikahil M. P. T. (2020). Altered metabolomic profiling of overweight and obese adolescents after combined training is associated with reduced insulin resistance. Sci. Rep. 10 (1), 16880. 10.1038/s41598-020-73943-y 33037261 PMC7547065

[B13] Edström L. Ekblom B. (1972). Differences in sizes of red and white muscle fibres in vastus lateralis of musculus quadriceps femoris of normal individuals and athletes. Relation to physical performance. Scand. J. Clin. Laboratory Investigation 30 (2), 175–181. 10.3109/00365517209081108 4640627

[B14] Farpour-Lambert N. J. Aggoun Y. Marchand L. M. Martin X. E. Herrmann F. R. Beghetti M. (2009). Physical activity reduces systemic blood pressure and improves early markers of atherosclerosis in pre-pubertal obese children. J. Am. Coll. Cardiol. 54 (25), 2396–2406. 10.1016/j.jacc.2009.08.030 20082930

[B15] Farup J. Kjølhede T. Sørensen H. Dalgas U. Møller A. B. Vestergaard P. F. (2012). Muscle morphological and strength adaptations to endurance vs. resistance training. J. Strength Cond. Res. 26 (2), 398–407. 10.1519/JSC.0b013e318225a26f 22266546

[B16] Freedman D. S. Mei Z. Srinivasan S. R. Berenson G. S. Dietz W. H. (2007). Cardiovascular risk factors and excess adiposity among overweight children and adolescents: The bogalusa heart study. J. Pediatr. 150 (1), 12–17.e2. 10.1016/j.jpeds.2006.08.042 17188605

[B17] Fry A. C. (2004). The role of resistance exercise intensity on muscle fibre adaptations. Sports Med. Auckl. N.Z.) 34 (10), 663–679. 10.2165/00007256-200434100-00004 15335243

[B18] Gäbler M. Prieske O. Hortobágyi T. Granacher U. (2018). The effects of concurrent strength and endurance training on physical fitness and athletic performance in youth: A systematic review and meta-analysis. Front. Physiology 9, 1057. 10.3389/fphys.2018.01057 PMC6090054 30131714

[B19] George K. Kristi K. Russell P. (2017). Exercise and BMI z-score in overweight and obese children and adolescents: A systematic review and network meta-analysis of randomized trials. J. evidence-based Med. 10 (2), 108–128. 10.1111/jebm.12228 PMC5553313 27792271

[B20] Giovanni F. Iuliano E. Aquino G. Campanella E. Tsopani D. Di Costanzo A. (2017). Different consecutive training protocols to design an intervention program for overweight youth: A controlled study. Diabetes, metabolic syndrome Obes. 10, 37–45. 10.2147/DMSO.S122110 PMC5248930 28144155

[B21] Grgic J. Mcllvenna L. C. Fyfe J. J. Sabol F. Bishop D. J. Schoenfeld B. J. (2019). Does aerobic training promote the same skeletal muscle hypertrophy as resistance training? A systematic review and meta-analysis. Sports Med. 49 (2), 233–254. 10.1007/s40279-018-1008-z 30341595

[B22] Gutin B. Barbeau P. Owens S. Lemmon C. R. Bauman M. Allison J. (2002). Effects of exercise intensity on cardiovascular fitness, total body composition, and visceral adiposity of obese adolescents. Am. J. Clin. Nutr. 75 (5), 818–826. 10.1093/ajcn/75.5.818 11976154

[B23] Hamilton D. Dee A. Perry I. J. (2018). The lifetime costs of overweight and obesity in childhood and adolescence: A systematic review. Obes. Rev. 19 (4), 452–463. 10.1111/obr.12649 29271111

[B1] Higgins J. P. T. Thomas J. Chandler J. Cumpston M. Li T. Page M. J. (editors) (2019). Cochrane Handbook for Systematic Reviews of Interventions. 2nd Edn. Chichester, UK: John Wiley & Sons.

[B24] Higgins J. P. T. Thompson S. G. (2002). Quantifying heterogeneity in a meta-analysis. Statistics Med. 21 (11), 1539–1558. 10.1002/sim.1186 12111919

[B25] Hutton B. Salanti G. Caldwell D. M. Chaimani A. Schmid C. H. Cameron C. (2015). The PRISMA extension statement for reporting of systematic reviews incorporating network meta-analyses of health care interventions: Checklist and explanations. Ann. Intern. Med. 162 (11), 777–784. 10.7326/M14-2385 26030634

[B26] Jansen J. P. Schmid C. H. Salanti G. (2012). Directed acyclic graphs can help understand bias in indirect and mixed treatment comparisons. J. Clin. Epidemiol. 65 (7), 798–807. 10.1016/j.jclinepi.2012.01.002 22521579

[B27] Jebeile H. Kelly A. S. O'Malley G. Baur L. A. (2022). Obesity in children and adolescents: Epidemiology, causes, assessment, and management. Lancet Diabetes and Endocrinol. 10 (5), 351–365. 10.1016/S2213-8587(22)00047-X 35248172 PMC9831747

[B28] Jeon J.-Y. Han J. Kim H. J. Park M. S. Seo D. Y. Kwak Y. S. (2013). The combined effects of physical exercise training and detraining on adiponectin in overweight and obese children. Integr. Med. Res. 2 (4), 145–150. 10.1016/j.imr.2013.10.001 28664066 PMC5481692

[B29] Karacabey K. (2009). The effect of exercise on leptin, insulin, cortisol and lipid profiles in obese children. J. Int. Med. Res. 37 (5), 1472–1478. 10.1177/147323000903700523 19930853

[B30] Kelley G. A. Kelley K. S. Pate R. R. (2015). Exercise and BMI in overweight and obese children and adolescents: a systematic review and trial sequential meta-analysis. Biomed Res. Int., 1–17. 10.1155/2015/704539 PMC4633529 26579538

[B31] Kelly A. S. Barlow S. E. Rao G. Inge T. H. Hayman L. L. Steinberger J. (2013). Severe obesity in children and adolescents: Identification, associated health risks, and treatment approaches: A scientific statement from the American heart association. Circulation 128 (15), 1689–1712. 10.1161/CIR.0b013e3182a5cfb3 24016455

[B32] Khammassi M. Ouerghi N. Hadj-Taieb S. Feki M. Thivel D. Bouassida A. (2018). Impact of a 12-week high-intensity interval training without caloric restriction on body composition and lipid profile in sedentary healthy overweight/obese youth. J. Exerc. Rehabilitation 14 (1), 118–125. 10.12965/jer.1835124.562 PMC5833956 29511662

[B33] Kim J. Son W. M. Headid Iii R. J. Pekas E. J. Noble J. M. Park S. Y. (2019). The effects of a 12-week jump rope exercise program on body composition, insulin sensitivity, and academic self-efficacy in obese adolescent girls. J. Pediatr. Endocrinol. Metabolism 33 (1), 129–137. 10.1515/jpem-2019-0327 31812946

[B34] Lazzer S. Lafortuna C. Busti C. Galli R. Agosti F. Sartorio A. (2011). Effects of low- and high-intensity exercise training on body composition and substrate metabolism in obese adolescents. J. Endocrinol. Investigation 34 (1), 45–52. 10.1007/BF03346694 20808072

[B35] Lee S. Bacha F. Hannon T. Kuk J. L. Boesch C. Arslanian S. (2012). Effects of aerobic versus resistance exercise without caloric restriction on abdominal fat, intrahepatic lipid, and insulin sensitivity in obese adolescent boys: A randomized, controlled trial. Diabetes 61 (11), 2787–2795. 10.2337/db12-0214 22751691 PMC3478522

[B36] Lee S. Deldin A. R. White D. Kim Y. Libman I. Rivera-Vega M. (2013). Aerobic exercise but not resistance exercise reduces intrahepatic lipid content and visceral fat and improves insulin sensitivity in obese adolescent girls: A randomized controlled trial. Am. J. Physiology Endocrinol. Metabolism 305 (10), E1222–E1229. 10.1152/ajpendo.00285.2013 PMC3840217 24045865

[B37] Lee Y. H. Song Y. W. Kim H. S. Lee S. Y. Jeong H. S. Suh S. H. (2010). The effects of an exercise program on anthropometric, metabolic, and cardiovascular parameters in obese children. Korean Circulation J. 40 (4), 179–184. 10.4070/kcj.2010.40.4.179 20421958 PMC2859335

[B38] Li T. Puhan M. A. Vedula S. S. Singh S. Dickersin K. Ad Hoc Network Meta-analysis Methods Meeting Working Gro up (2011). Network meta-analysis-highly attractive but more methodological research is needed. BMC Med. 9 (1), 79. 10.1186/1741-7015-9-79 21707969 PMC3159133

[B39] Lobstein C. T. Brinsden H. (2019). Atlas of childhood obesity. London: World Obesity Federation’.

[B40] Martín-García M. Alegre L. M. García-Cuartero B. Bryant E. J. Gutin B. Ara I. (2019). Effects of a 3-month vigorous physical activity intervention on eating behaviors and body composition in overweight and obese boys and girls. J. Sport Health Sci. 8 (2), 170–176. 10.1016/j.jshs.2017.09.012 30997263 PMC6450925

[B41] Martins R. A. Neves A. P. Coelho-Silva M. J. Veríssimo M. T. Teixeira A. M. (2010). The effect of aerobic versus strength-based training on high-sensitivity C-reactive protein in older adults. Eur. J. Appl. Physiology 110 (1), 161–169. 10.1007/s00421-010-1488-5 20437055

[B42] Medicine A. C. of S. (2013). ACSM’s guidelines for exercise testing and prescription. 9th ed. Lippincott Williams and Wilkins.

[B43] Méndez-Hernández L. D. Ramírez-Moreno E. Barrera-Gálvez R. Cabrera-Morales M. D. C. Reynoso-Vázquez J. Flores-Chávez O. R. (2022). Effects of strength training on body fat in children and adolescents with overweight and obesity: A systematic review with meta-analysis. Children 9 (7), 995. 10.3390/children9070995 35883978 PMC9319224

[B44] Monteiro P. A. Chen K. Y. Lira F. S. Saraiva B. T. C. Antunes B. M. M. Campos E. Z. (2015). Concurrent and aerobic exercise training promote similar benefits in body composition and metabolic profiles in obese adolescents. Lipids Health Dis. 14, 153. 10.1186/s12944-015-0152-9 26611872 PMC4660803

[B45] Nakhostin-Roohi B. Havaskar S. (2018). The Effect of Concurrent Training Program on Body Composition Indices in Overweight and Obese Female Students, 1, 6–12.

[B46] Nikolakopoulou A. Higgins J. P. T. Papakonstantinou T. Chaimani A. Del Giovane C. Egger M. (2020). CINeMA: An approach for assessing confidence in the results of a network meta-analysis. PLoS Med. 17 (4), e1003082. 10.1371/journal.pmed.1003082 32243458 PMC7122720

[B47] O’Donoghue G. Blake C. Cunningham C. Lennon O. Perrotta C. (2021). What exercise prescription is optimal to improve body composition and cardiorespiratory fitness in adults living with obesity? A network meta-analysis. Obes. Rev. 22 (2), e13137. 10.1111/obr.13137 32896055 PMC7900983

[B48] Papakonstantinou T. Nikolakopoulou A. Higgins J. P. T. Egger M. Salanti G. (2020). CINeMA: Software for semiautomated assessment of the confidence in the results of network meta-analysis. Campbell Syst. Rev. 16 (1), e1080. 10.1002/cl2.1080 37131978 PMC8356302

[B49] Peirson L. Fitzpatrick-Lewis D. Morrison K. Ciliska D. Kenny M. Usman Ali M. (2015). Prevention of overweight and obesity in children and youth: A systematic review and meta-analysis. CMAJ open 3 (1), E23–E33. 10.9778/cmajo.20140053 PMC4382039 25844367

[B50] Peirson L. Fitzpatrick-Lewis D. Morrison K. Warren R. Usman Ali M. Raina P. (2015). Treatment of overweight and obesity in children and youth: A systematic review and meta-analysis. CMAJ open 3 (1), E35–E46. 10.9778/cmajo.20140047 PMC4382035 25844368

[B51] Pont S. J. Puhl R. Cook S. R. Slusser W. SECTION ON OBESITY and OBESITY SOCIETY (2017). Stigma experienced by children and adolescents with obesity. Pediatrics 140 (6), e20173034. 10.1542/peds.2017-3034 29158228

[B52] Puhl R. M. Lessard L. M. (2020). Weight stigma in youth: Prevalence, consequences, and considerations for clinical practice. Curr. Obes. Rep. 9 (4), 402–411. 10.1007/s13679-020-00408-8 33079337

[B53] Racil G. Coquart J. B. Elmontassar W. Haddad M. Goebel R. Chaouachi A. (2016). Greater effects of high-compared with moderate-intensity interval training on cardio-metabolic variables, blood leptin concentration and ratings of perceived exertion in obese adolescent females. Biol. Sport 33 (2), 145–152. 10.5604/20831862.1198633 27274107 PMC4885625

[B54] Regaieg S. Charfi N. Kamoun M. Ghroubi S. Rebai H. Elleuch H. (2012). The effects of an exercise training program on body composition and aerobic capacity parameters in Tunisian obese children. Indian J. Endocrinol. Metabolism 17 (6), 1040–1045. 10.4103/2230-8210.122619 PMC3872682 24381881

[B55] Rhea M. R. Alvar B. A. Burkett L. N. Ball S. D. (2003). A meta-analysis to determine the dose response for strength development. Med. Sci. Sports Exerc. 35 (3), 456–464. 10.1249/01.MSS.0000053727.63505.D4 12618576

[B56] Said M. A. Abdelmoneim M. A. Alibrahim M. S. Kotb A. A. H. (2021). Aerobic training, resistance training, or their combination as a means to fight against excess weight and metabolic syndrome in obese students - which is the most effective modality? A randomized controlled trial. Appl. Physiology, Nutr. Metabolism 46 (8), 952–963. 10.1139/apnm-2020-0972 33630712

[B57] Salus M. Tillmann V. Remmel L. Unt E. Mäestu E. Parm Ü. (2022). Effect of supervised sprint interval training on cardiorespiratory fitness and body composition in adolescent boys with obesity. J. Sports Sci. 40 (18), 2010–2017. 10.1080/02640414.2022.2125199 36126151

[B58] Saygın O. (2011). The effect of twelve week aerobic exercise programme on health related physical fitness components and blood lipids in obese girls. Afr. J. Pharm. Pharmacol. 5 (12), 1441–1445. 10.5897/AJPP11.114

[B59] Schranz N. Tomkinson G. Olds T. (2013). What is the effect of resistance training on the strength, body composition and psychosocial status of overweight and obese children and adolescents? A systematic review and meta-analysis. Sports Med. 43 (9), 893–907. 10.1007/s40279-013-0062-9 23729196

[B60] Schwingshackl L. Dias S. Strasser B. Hoffmann G. (2013). Impact of different training modalities on anthropometric and metabolic characteristics in overweight/obese subjects: A systematic review and network meta-analysis. PLoS ONE 8 (12), e82853. 10.1371/journal.pone.0082853 24358230 PMC3866267

[B61] Shaibi G. Q. Cruz M. L. Ball G. D. C. Weigensberg M. J. Salem G. J. Crespo N. C. (2006). Effects of resistance training on insulin sensitivity in overweight Latino adolescent males. Med. Sci. Sports Exerc. 38 (7), 1208–1215. 10.1249/01.mss.0000227304.88406.0f 16826016

[B62] Sigal R. J. Alberga A. S. Goldfield G. S. Prud'homme D. Hadjiyannakis S. Gougeon R. (2014). Effects of aerobic training, resistance training, or both on percentage body fat and cardiometabolic risk markers in obese adolescents: The healthy eating aerobic and resistance training in youth randomized clinical trial. JAMA Pediatr. 168 (11), 1006–1014. 10.1001/jamapediatrics.2014.1392 25243536

[B63] Sinha R. Fisch G. Teague B. Tamborlane W. V. Banyas B. Allen K. (2002). Prevalence of impaired glucose tolerance among children and adolescents with marked obesity. N. Engl. J. Med. 346 (11), 802–810. 10.1056/NEJMoa012578 11893791

[B64] Song J. K. Stebbins C. L. Kim T. K. Kim H. B. Kang H. J. Chai J. H. (2011). Effects of 12 weeks of aerobic exercise on body composition and vascular compliance in obese boys. J. Sports Med. Phys. Fit. 52 (5), 522–529. 22976739

[B65] Sterne J. A. C. Savović J. Page M. J. Elbers R. G. Blencowe N. S. Boutron I. (2019). RoB 2: A revised tool for assessing risk of bias in randomised trials. BMJ Clin. Res. ed. 366, l4898. 10.1136/bmj.l4898 31462531

[B66] Stewart A. Sutton L. (Editors) (2012). Body composition in sport, exercise and health (London: Routledge). 10.4324/9780203133040

[B67] Tan S. Chen C. Sui M. Xue L. Wang J. (2017). Exercise training improved body Composition,Cardiovascular function, and physical fitness of 5-year-old children with obesity or normal body mass. Pediatr. Exerc. Sci. 29 (2), 245–253. 10.1123/pes.2016-0107 27768546

[B68] Tan S. Wang J. Cao L. (2016). Exercise training at the intensity of maximal fat oxidation in obese boys. Appl. Physiology, Nutr. Metabolism 41 (1), 49–54. 10.1139/apnm-2015-0174 26701116

[B69] Vasconcellos F. Seabra A. Cunha F. Montenegro R. Penha J. Bouskela E. (2015). Health markers in obese adolescents improved by a 12-week recreational soccer program: A randomised controlled trial. J. Sports Sci. 34 (6), 564–575. 10.1080/02640414.2015.1064150 26208409

[B70] Watt J. Del Giovane C. (2022). Network meta-analysis. Methods Mol. Biol. Clift. N.J.) 2345, 187–201. 10.1007/978-1-0716-1566-9_12 34550592

[B71] Wilhelm E. N. Pinto R. S. (2019). “Concurrent aerobic and strength training for body composition and health,” in Concurrent aerobic and strength training: Scientific basics and practical applications. Editors Schumann M. Rønnestad B. R. (Cham: Springer International Publishing), 293–307. 10.1007/978-3-319-75547-2_19

[B72] Willis L. H. Slentz C. A. Bateman L. A. Shields A. T. Piner L. W. Bales C. W. (2012). Effects of aerobic and/or resistance training on body mass and fat mass in overweight or obese adults. J. Appl. Physiology 113 (12), 1831–1837. 10.1152/japplphysiol.01370.2011 PMC3544497 23019316

[B73] Wilson J. M. Marin P. J. Rhea M. R. Wilson S. M. C. Loenneke J. P. Anderson J. C. (2012). Concurrent training: A meta-analysis examining interference of aerobic and resistance exercises. J. Strength and Cond. Res. 26 (8), 2293–2307. 10.1519/JSC.0b013e31823a3e2d 22002517

[B74] Wong A. Sanchez-Gonzalez M. A. Son W. M. Kwak Y. S. Park S. Y. (2018). The effects of a 12-week combined exercise training program on arterial stiffness, vasoactive substances, inflammatory markers, metabolic profile, and body composition in obese adolescent girls. Pediatr. Exerc. Sci. 30 (4), 480–486. 10.1123/pes.2017-0198 30193554

[B75] Wong P. Chia M. Y. H. Tsou I. Y. Y. Wansaicheong G. K. L. Tan B. Wang J. C. K. (2008)., 37. Singapore, 286–293. Effects of a 12-week exercise training programme on aerobic fitness, body composition, blood lipids and C-reactive protein in adolescents with obesity. Ann. Acad. Med. 10.47102/annals-acadmedsg.v37n4p286 18461212

[B76] Youssef H. Groussard C. Lemoine-Morel S. Pincemail J. Jacob C. Moussa E. (2015). Aerobic training suppresses exercise-induced lipid peroxidation and inflammation in overweight/obese adolescent girls. Pediatr. Exerc. Sci. 27 (1), 67–76. 10.1123/pes.2014-0008 25387489

[B77] Zouhal H. Lemoine-Morel S. Mathieu M. E. Casazza G. A. Jabbour G. (2013). Catecholamines and obesity: Effects of exercise and training. Sports Med. Auckl. N.Z.) 43 (7), 591–600. 10.1007/s40279-013-0039-8 23613311

