# Supplementary material for: Effects of different exercise modalities and intensities on body composition in overweight and obese children and adolescents: a systematic review and network meta-analysis
Source: Front Physiol. 2023 Jul 11;14:1193223. doi: 10.3389/fphys.2023.1193223 (PMC10366610; doi:10.3389/fphys.2023.1193223)
Supplement: Supplementary file 1 [file DataSheet1.PDF]

Supplementary Material

Effects of Different Exercise Modalities and Intensities on Body Composition in Overweight and Obese Children and Adolescents: A Systematic Review and Network Meta-Analysis

Zan Huang, Jiayu Li, Yanjie Liu, Yulan Zhou*

*** Correspondence:**

Yulan Zhou

zhouyulan004@outlook.com

1 Supplementary Tables and Figures

1.1 Supplementary Tables

Table S1 PRISMA Network Meta-Analysis Checklist

Table S2 Search Strategy

Table S3 Risk of Bias Assessment Results

Table S4 Confidence in Network Meta-analysis (CINeMA) Report

Table S5 Estimated Global Inconsistency in Networks

Table S6 Estimated Local Inconsistency for Each Pairwise Comparison

1.2 Supplementary Figures

Figure S1 Summary of The Risk of Bias Assessment

Figure S2 Confidence in Network Meta-analysis (CINeMA) Report

Figure S3 Network Forest

Figure S4 Pairwise Meta-Analysis Forest Plot

Figure S5 Network Meta-Analysis Results—Interval Plot

Figure S6 Loop Inconsistency Plots.

Figure S7 Contribution Matrix of Direct and Indirect Evidence

Figure S8 SUCRA Values for Each Intervention

Figure S9 Funnel Plot Graphics

Table S1 PRISMA Network Meta-Analysis Checklist

Section/Topic	Item #	Checklist Item	Reported on Page #
**TITLE**			
Title	1	Identify the report as a systematic review *incorporating a network meta-analysis (or related form of meta-analysis).*	1
**ABSTRACT**			
Structured summary	2	Provide a structured summary including, as applicable:<br>**Background:** main objectives<br>**Methods:** data sources; study eligibility criteria, participants, and interventions; study appraisal; and *synthesis methods, such as network meta-analysis.*<br>**Results:** number of studies and participants identified; summary estimates with corresponding confidence/credible intervals; *treatment rankings may also be discussed. Authors may choose to summarize pairwise comparisons against a chosen treatment included in their analyses for brevity.*<br>**Discussion/Conclusions:** limitations; conclusions and implications of findings.<br>**Other:** primary source of funding; systematic review registration number with registry name.	1-2
**INTRODUCTION**			
Rationale	3	Describe the rationale for the review in the context of what is already known, *including mention of why a network meta-analysis has been conducted.*	2
Objectives	4	Provide an explicit statement of questions being addressed, with reference to participants, interventions, comparisons, outcomes, and study design (PICOS).	2-3
**METHODS**			
Protocol and registration	5	Indicate whether a review protocol exists and if and where it can be accessed (e.g., Web address); and, if available, provide registration information, including registration number.	3
Eligibility criteria	6	Specify study characteristics (e.g., PICOS, length of follow-up) and report characteristics (e.g., years considered, language, publication status) used as criteria for eligibility, giving rationale. *Clearly describe eligible treatments included in the treatment network, and note whether any have been clustered or merged into the same node (with justification).*	3-4
Information sources	7	Describe all information sources (e.g., databases with dates of coverage, contact with study authors to identify additional studies) in the search and date last searched.	3
Search	8	Present full electronic search strategy for at least one database, including any limits used, such that it could be repeated.	3
Study selection	9	State the process for selecting studies (i.e., screening, eligibility, included in systematic review, and, if applicable,	4

		included in the meta-analysis).	
Data collection process	10	Describe method of data extraction from reports (e.g., piloted forms, independently, in duplicate) and any processes for obtaining and confirming data from investigators.	4
Data items	11	List and define all variables for which data were sought (e.g., PICOS, funding sources) and any assumptions and simplifications made.	3-4
**Geometry of the network**	**S1**	Describe methods used to explore the geometry of the treatment network under study and potential biases related to it. This should include how the evidence base has been graphically summarized for presentation, and what characteristics were compiled and used to describe the evidence base to readers.	5
Risk of bias within individual studies	12	Describe methods used for assessing risk of bias of individual studies (including specification of whether this was done at the study or outcome level), and how this information is to be used in any data synthesis.	4
Summary measures	13	State the principal summary measures (e.g., risk ratio, difference in means). *Also describe the use of additional summary measures assessed, such as treatment rankings and surface under the cumulative ranking curve (SUCRA) values, as well as modified approaches used to present summary findings from meta-analyses.*	5
Planned methods of analysis	14	Describe the methods of handling data and combining results of studies for each network meta-analysis. This should include, but not be limited to:<br>• *Handling of multi-arm trials;*<br>• *Selection of variance structure;*<br>• *Selection of prior distributions in Bayesian analyses; and*<br>• *Assessment of model fit.*	5
**Assessment of Inconsistency**	**S2**	Describe the statistical methods used to evaluate the agreement of direct and indirect evidence in the treatment network(s) studied. Describe efforts taken to address its presence when found.	5
Risk of bias across studies	15	Specify any assessment of risk of bias that may affect the cumulative evidence (e.g., publication bias, selective reporting within studies).	4
Additional analyses	16	Describe methods of additional analyses if done, indicating which were pre-specified. This may include, but not be limited to, the following:<br>• Sensitivity or subgroup analyses;<br>• Meta-regression analyses;<br>• *Alternative formulations of the treatment network; and*<br>• *Use of alternative prior distributions for Bayesian analyses (if applicable).*	

RESULTS†

Study selection	17	Give numbers of studies screened, assessed for eligibility, and included in the review, with reasons for exclusions at each stage, ideally with a flow diagram.	5
**Presentation of network structure**	**S3**	Provide a network graph of the included studies to enable visualization of the geometry of the treatment network.	6
**Summary of network geometry**	**S4**	Provide a brief overview of characteristics of the treatment network. This may include commentary on the abundance of trials and randomized patients for the different interventions and pairwise comparisons in the network, gaps of evidence in the treatment network, and potential biases reflected by the network structure.	6
Study characteristics	18	For each study, present characteristics for which data were extracted (e.g., study size, PICOS, follow-up period) and provide the citations.	5
Risk of bias within studies	19	Present data on risk of bias of each study and, if available, any outcome level assessment.	6
Results of individual studies	20	For all outcomes considered (benefits or harms), present, for each study: 1) simple summary data for each intervention group, and 2) effect estimates and confidence intervals. *Modified approaches may be needed to deal with information from larger networks.*	Table S3 (Online pp.5-6)
Synthesis of results	21	Present results of each meta-analysis done, including confidence/credible intervals. *In larger networks, authors may focus on comparisons versus a particular comparator (e.g. placebo or standard care), with full findings presented in an appendix. League tables and forest plots may be considered to summarize pairwise comparisons.* If additional summary measures were explored (such as treatment rankings), these should also be presented.	6-8
**Exploration for inconsistency**	**S5**	Describe results from investigations of inconsistency. This may include such information as measures of model fit to compare consistency and inconsistency models, *P* values from statistical tests, or summary of inconsistency estimates from different parts of the treatment network.	Table S5-S6 (Online pp.12-14)
Risk of bias across studies	22	Present results of any assessment of risk of bias across studies for the evidence base being studied.	Table S4 (Online pp.7-11)
Results of additional analyses	23	Give results of additional analyses, if done (e.g., sensitivity or subgroup analyses, meta-regression analyses, *alternative network geometries studied, alternative choice of prior distributions for Bayesian analyses*, and so forth).	

DISCUSSION

Summary of evidence	24	Summarize the main findings, including the strength of evidence for each main outcome; consider their relevance to key groups (e.g., healthcare providers, users, and policy-makers).	8
Limitations	25	Discuss limitations at study and outcome level (e.g., risk of bias), and at review level (e.g., incomplete retrieval of identified research, reporting bias). *Comment on the validity of the assumptions, such as transitivity and consistency. Comment*	9

		on any concerns regarding network geometry (e.g., avoidance of certain comparisons).	
Conclusions	26	Provide a general interpretation of the results in the context of other evidence, and implications for future research.	10
**FUNDING**			
Funding	27	Describe sources of funding for the systematic review and other support (e.g., supply of data); role of funders for the systematic review. This should also include information regarding whether funding has been received from manufacturers of treatments in the network and/or whether some of the authors are content experts with professional conflicts of interest that could affect use of treatments in the network.	11

PICOS = population, intervention, comparators, outcomes, study design.
* Text in italics indicateS wording specific to reporting of network meta-analyses that has been added to guidance from the PRISMA statement.
† Authors may wish to plan for use of appendices to present all relevant information in full detail for items in this section.

Table S2 Search Strategy

PubMed<br><br>(("Exercise"[Title] OR "physical activit*"[Title] OR "Aerobic Exercise"[Title] OR "Muscle Stretching Exercise"[Title] OR "Physical Exercise"[Title] OR exercise[Title] OR sport*[Title] OR aerobic[Title] OR resistance[Title] OR movement[Title] OR workout[Title] OR "strength training"[Title] OR "combined training"[Title] OR "endurance training"[Title] OR "concurrent training"[Title] OR "circuit training"[Title] OR "HIIT training"[Title] OR "interval training"[Title]) AND ("body weight"[Title] OR "body mass index"[Title] OR BMI[Title] OR "body fat percentage"[Title] OR "body fat"[Title] OR "fat mass"[Title] OR "body composition"[Title] OR "weight loss"[Title] OR "body mass"[Title] OR obesity[Title] OR overweight[Title] OR obese[Title] OR "waist circumference"[Title])) AND (child*[Title] OR children[Title] OR adolescent*[Title] OR juvenile*[Title] OR teen*[Title] OR teenager*[Title] OR youth[Title] OR preschool*[Title] OR kindergarten*[Title] OR student*[Title])
Web of Science<br><br>((TI=(Exercise OR "physical activit*" OR "Aerobic Exercise" OR "Muscle Stretching Exercise" OR "Physical Exercise" OR exercise OR sport* OR aerobic OR resistance OR movement OR workout OR "strength training" OR "combined training" OR "endurance training" OR "concurrent training" OR "circuit training" OR "HIIT training" OR "interval training")) AND TI=("body weight" OR "body mass index" OR BMI OR "body fat percentage" OR "body fat" OR "fat mass" OR "body composition" OR "weight loss" OR "body mass" OR obesity OR overweight OR obese OR "waist circumference")) AND AB=(child* OR children OR adolescent* OR juvenile* OR teen* OR teenager* OR youth OR preschool* OR kindergarten* OR student*)
Scopus<br><br>(TITLE ("Exercise" OR "physical activit*" OR "Aerobic Exercise" OR "Muscle Stretching Exercise" OR "Physical Exercise" OR exercise OR sport* OR aerobic OR resistance OR movement OR workout OR "strength training" OR "combined training" OR "endurance training" OR "concurrent training" OR "circuit training" OR "HIIT training" OR "interval training") AND TITLE ("body weight" OR "body mass index" OR bmi OR "body fat

percentage" OR "body fat" OR "fat mass" OR "body composition" OR "weight loss" OR "body mass" OR obesity OR overweight OR obese OR "waist circumference") AND TITLE (child* OR children OR adolescent* OR juvenile* OR teen* OR teenager* OR youth OR preschool* OR kindergarten* OR student*))
EMBASE<br><br>(TITLE ("Exercise" OR "physical activit*" OR "Aerobic Exercise" OR "Muscle Stretching Exercise" OR "Physical Exercise" OR exercise OR sport* OR aerobic OR resistance OR movement OR workout OR "strength training" OR "combined training" OR "endurance training" OR "concurrent training" OR "circuit training" OR "HIIT training" OR "interval training") AND TITLE ("body weight" OR "body mass index" OR bmi OR "body fat percentage" OR "body fat" OR "fat mass" OR "body composition" OR "weight loss" OR "body mass" OR obesity OR overweight OR obese OR "waist circumference") AND TITLE (child* OR children OR adolescent* OR juvenile* OR teen* OR teenager* OR youth OR preschool* OR kindergarten* OR student*))

Table S3 Risk of bias assessment results

	Randomization process	Deviations from intended interventions	Missing outcome data	Measurement of the outcome	Selection of the reported result	
SHAIBI 2006	High	Low	Low	Low	Low	High
Wong 2008	Some concerns	Some concerns	Low	Low	Low	Some concerns
KARACABEY 2009	Some concerns	Low	Low	Low	Low	Some concerns
Farpour-Lambert 2009	Low	Some concerns	Low	Low	Some concerns	Some concerns
Lee 2010	High	High	Low	Low	Some concerns	High
Saygın 2011	Low	Some concerns	Low	Low	Low	Some concerns
SONG 2012	Some concerns	Some concerns	Low	Low	Some concerns	Some concerns
Lee 2012	Low	Low	Low	Low	Low	Low
Regaieg 2012	Some concerns	Low	Low	Low	Some concerns	Some concerns
Lee 2013	Low	Low	Low	Low	Low	Low
Alberga 2013	Low	Some concerns	Low	Low	Low	Some concerns
Jeon 2013	High	Some concerns	Low	Low	Some concerns	High
Sigal 2014	Low	Some concerns	Low	Low	Low	Some concerns
Youssef 2015	Low	Some concerns	Low	Low	Some concerns	Some concerns
Monteiro 2015	Some concerns	Low	Low	Low	Some concerns	Some concerns
Tan 2016	Some concerns	Some concerns	Low	Low	Some concerns	Some concerns
Vasconcellos 2015	Low	Some concerns	Low	Low	Low	Some concerns
Racil 2016	Some concerns	Some concerns	Low	Low	Some concerns	Some concerns
Fiorilli 2017	Low	Some concerns	Low	Low	Some concerns	Some concerns
Tan 2017	Some concerns	Some concerns	Low	Some concerns	Some concerns	Some concerns
Khammassi 2018	High	Some concerns	Low	Low	Some concerns	High
Dias 2017	Low	Some concerns	Low	Low	Some concerns	Some concerns
Cvetković 2018	Some concerns	Some concerns	Low	Low	Some concerns	Some concerns
Wong 2017	Some concerns	Some concerns	Low	Low	Some concerns	Some concerns
Martín-García 2017	Some concerns	Some concerns	Low	Low	Some concerns	Some concerns
Deldin 2019	Some concerns	Some concerns	Low	Low	Some concerns	Some concerns
Kim 2019	Some concerns	Some concerns	Low	Low	Some concerns	Some concerns
Duft 2020	Some concerns	Some concerns	Low	Low	Some concerns	Some concerns

Said 2021	Low	Some concerns	Low	Low	Some concerns	Some concerns
Bouamra 2021	Some concerns	Some concerns	Low	Low	Some concerns	Some concerns
Cao 2022	Low	Some concerns	Low	Low	Some concerns	Some concerns
Salus 2022	Low	Some concerns	Low	Low	Some concerns	Some concerns
Salus 2022	Low	Some concerns	Low	Low	Some concerns	Some concerns

Table S4 Confidence in Network Meta-analysis (CINeMA) report

Table 1. Summary Grading of Evidence for Body Fat Percentage

Comparison	Number of studies	Within-study bias	Reporting bias	Indirectness	Imprecision	Heterogeneity	Incoherence	Confidence rating
**Mixed estimates**								
AE-M:AE-HI	3	Some concerns	Undetected	No concerns	Some concerns	Some concerns	No concerns	Low
AE-M:COM-M	1	Some concerns	Undetected	No concerns	Major concerns	No concerns	No concerns	Very low
AE-M:CON	11	No concern	Undetected	No concerns	No concerns	Some concerns	Major concerns	Low
AE-M:RT-HI	1	Some concerns	Undetected	No concerns	Major concerns	No concerns	No concerns	Very low
AE-M:RT-M	3	Some concerns	Undetected	No concerns	Major concerns	No concerns	Some concerns	Very low
AE-HI:COM-HI	3	Some concerns	Undetected	No concerns	Major concerns	No concerns	No concerns	Very low
AE-HI:CON	12	No concerns	Undetected	No concerns	No concerns	Some concerns	No concerns	Moderate
AE-HI:RT-HI	2	Some concerns	Undetected	No concerns	Major concerns	No concerns	No concerns	Very low
COM-HI:CON	2	No concerns	Undetected	No concerns	No concerns	Some concerns	No concerns	Moderate
COM-HI:RT-HI	2	Some concerns	Undetected	No concerns	Major concerns	No concerns	No concerns	Very low
COM-M:CON	5	No concerns	Undetected	No concerns	No concerns	Some concerns	No concerns	Moderate
COM-M:RT-M	1	Some concerns	Undetected	No concerns	Major concerns	No concerns	No concerns	Very low
CON:RT-HI	2	Some concerns	Undetected	No concerns	No concerns	Major concerns	No concerns	Very low
CON:RT-M	3	No concerns	Undetected	No concerns	No concerns	Major concerns	Some concerns	Low
RT-HI:RT-M	1	Some concerns	Undetected	No concerns	Major concerns	No concerns	No concerns	Very low
**Indirect estimates**								
AE-M:COM-HI	0	Some concerns	Undetected	No concerns	Major concerns	No concerns	No concerns	Very low
AE-HI:COM-M	0	Some concerns	Undetected	No concerns	Some concerns	Some concerns	No concerns	Low
AE-HI:RT-M	0	Some concerns	Undetected	No concerns	Major concerns	No concerns	No concerns	Very low
COM-HI:COM-M	0	Some concerns	Undetected	No concerns	Major concerns	No concerns	No concerns	Very low
COM-HI:RT-M	0	Some concerns	Undetected	No concerns	Major concerns	No concerns	No concerns	Very low
COM-M:RT-HI	0	Some concerns	Undetected	No concerns	Major concerns	No concerns	No concerns	Very low

AE-M, moderate-intensity aerobic exercise; AE-HI, high-intensity aerobic exercise; RT-M, moderate-intensity resistance exercise; RT-HI, high-intensity resistance exercise; COM-M, moderate-intensity combined exercise; COM-HI, high-intensity combined exercise; CON, blank controls

Table 2. Summary Grading of Evidence for Body Mass Index

Comparison	Number of studies	Within-study bias	Reporting bias	Indirectness	Imprecision	Heterogeneity	Incoherence	Confidence rating
**Mixed estimates**								
AE-M:AE-HI	2	Some concerns	Undetected	No concerns	Some concerns	Some concerns	No concerns	Low
AE-M:COM-M	1	Some concerns	Undetected	No concerns	Major concerns	No concerns	No concerns	Very low
AE-M:CON	12	Some concerns	Undetected	No concerns	No concerns	Some concerns	No concerns	Moderate
AE-M:RT-HI	1	Some concerns	Undetected	No concerns	No concerns	Major concerns	No concerns	Very low
AE-M:RT-M	6	Some concerns	Undetected	No concerns	Some concerns	Some concerns	Some concerns	Low
AE-HI:COM-HI	2	Some concerns	Undetected	No concerns	Major concerns	No concerns	No concerns	Very low
AE-HI:COM-M	1	Some concerns	Undetected	No concerns	Some concerns	Some concerns	No concerns	Low
AE-HI:CON	10	Some concerns	Undetected	No concerns	No concerns	Some concerns	No concerns	Moderate
AE-HI:RT-HI	1	Some concerns	Undetected	No concerns	Some concerns	Some concerns	No concerns	Low
COM-HI:CON	1	Some concerns	Undetected	No concerns	No concerns	Some concerns	No concerns	Moderate
COM-HI:RT-HI	1	Some concerns	Undetected	No concerns	Some concerns	Some concerns	No concerns	Low
COM-M:CON	6	Some concerns	Undetected	No concerns	No concerns	No concerns	No concerns	Moderate
COM-M:RT-M	1	Some concerns	Undetected	No concerns	Some concerns	Some concerns	No concerns	Low
CON:RT-HI	1	Some concerns	Undetected	No concerns	Some concerns	Some concerns	No concerns	Low
CON:RT-M	4	Some concerns	Undetected	No concerns	No concerns	Major concerns	Some concerns	Very low
RT-HI:RT-M	1	Some concerns	Undetected	No concerns	Major concerns	No concerns	No concerns	Very low
**Indirect estimates**								
AE-M:COM-HI	0	Some concerns	Undetected	No concerns	Major concerns	No concerns	Major concerns	Very low
AE-HI:RT-M	0	Some concerns	Undetected	No concerns	Some concerns	Some concerns	Major concerns	Very low
COM-HI:COM-M	0	Some concerns	Undetected	No concerns	Major concerns	No concerns	Major concerns	Very low
COM-HI:RT-M	0	Some concerns	Undetected	No concerns	Some concerns	Some concerns	Major concerns	Very low
COM-M:RT-HI	0	Some concerns	Undetected	No concerns	No concerns	Major concerns	Major concerns	Very low

AE-M, moderate-intensity aerobic exercise; AE-HI, high-intensity aerobic exercise; RT-M, moderate-intensity resistance exercise; RT-HI, high-intensity resistance exercise; COM-M, moderate-intensity combined exercise; COM-HI, high-intensity combined exercise; CON, blank controls

Table 3. Summary Grading of Evidence for Fat Mass

Comparison	Number of studies	Within-study bias	Reporting bias	Indirectness	Imprecision	Heterogeneity	Incoherence	Confidence rating
**Mixed estimates**								
AE-M:AE-HI	2	Some concerns	Undetected	No concerns	Major concerns	No concerns	No concerns	Very low
AE-M:CON	5	Some concerns	Undetected	No concerns	No concerns	Some concerns	No concerns	Moderate
AE-HI:COM-HI	3	Some concerns	Undetected	No concerns	Major concerns	No concerns	No concerns	Very low
AE-HI:CON	7	Some concerns	Undetected	No concerns	No concerns	Some concerns	No concerns	Moderate
AE-HI:RT-HI	2	Some concerns	Undetected	No concerns	Major concerns	No concerns	No concerns	Very low
COM-HI:CON	2	Some concerns	Undetected	No concerns	No concerns	Some concerns	No concerns	Moderate
COM-HI:RT-HI	2	Some concerns	Undetected	No concerns	Some concerns	Some concerns	No concerns	Low
COM-M:CON	3	Some concerns	Undetected	No concerns	No concerns	Some concerns	No concerns	Moderate
CON:RT-HI	2	Some concerns	Undetected	No concerns	No concerns	Major concerns	No concerns	Very low
**Indirect estimates**								
AE-M:COM-HI	0	Some concerns	Undetected	No concerns	Major concerns	No concerns	No concerns	Very low
AE-M:COM-M	0	Some concerns	Undetected	No concerns	Major concerns	No concerns	No concerns	Very low
AE-M:RT-HI	0	Some concerns	Undetected	No concerns	Major concerns	No concerns	No concerns	Very low
AE-HI:COM-M	0	Some concerns	Undetected	No concerns	Major concerns	No concerns	No concerns	Very low
COM-HI:COM-M	0	Some concerns	Undetected	No concerns	Major concerns	No concerns	No concerns	Very low

AE-M, moderate-intensity aerobic exercise; AE-HI, high-intensity aerobic exercise; RT-M, moderate-intensity resistance exercise; RT-HI, high-intensity resistance exercise; COM-M, moderate-intensity combined exercise; COM-HI, high-intensity combined exercise; CON, blank controls

Table 4. Summary Grading of Evidence for Fat-free Mass

Comparison	Number of studies	Within-study bias	Reporting bias	Indirectness	Imprecision	Heterogeneity	Incoherence	Confidence rating
**Mixed estimates**								
AE-M:AE-HI	1	Some concerns	Undetected	No concerns	Some concerns	Some concerns	No concerns	Low
AE-M:CON	6	Some concerns	Undetected	No concerns	Some concerns	Some concerns	No concerns	Low
AE-M:RT-HI	1	Some concerns	Undetected	No concerns	Some concerns	Some concerns	No concerns	Low
AE-M:RT-M	2	No concerns	Undetected	No concerns	Some concerns	Some concerns	No concerns	Low
AE-HI:COM-HI	2	Some concerns	Undetected	No concerns	Major concerns	No concerns	No concerns	Very low
AE-HI:CON	5	Some concerns	Undetected	No concerns	Some concerns	Some concerns	No concerns	Low
AE-HI:RT-HI	2	Some concerns	Undetected	No concerns	Some concerns	Some concerns	No concerns	Low
COM-HI:CON	1	Some concerns	Undetected	No concerns	Some concerns	Some concerns	Some concerns	Low
COM-HI:RT-HI	2	Some concerns	Undetected	No concerns	Some concerns	Some concerns	No concerns	Low
COM-M:CON	1	Some concerns	Undetected	No concerns	Major concerns	No concerns	No concerns	Very low
CON:RT-HI	2	Some concerns	Undetected	No concerns	No concerns	Some concerns	No concerns	Moderate
CON:RT-M	2	No concerns	Undetected	No concerns	Some concerns	Some concerns	No concerns	Moderate
RT-HI:RT-M	1	Some concerns	Undetected	No concerns	Major concerns	No concerns	No concerns	Low
**Indirect estimates**								
AE-M:COM-HI	0	Some concerns	Undetected	No concerns	Major concerns	No concerns	No concerns	Low
AE-M:COM-M	0	Some concerns	Undetected	No concerns	Major concerns	No concerns	No concerns	Low
AE-HI:COM-M	0	Some concerns	Undetected	No concerns	Major concerns	No concerns	No concerns	Low
AE-HI:RT-M	0	Some concerns	Undetected	No concerns	Major concerns	No concerns	No concerns	Low
COM-HI:COM-M	0	Some concerns	Undetected	No concerns	Major concerns	No concerns	No concerns	Low
COM-HI:RT-M	0	Some concerns	Undetected	No concerns	Major concerns	No concerns	No concerns	Low
COM-M:RT-HI	0	Some concerns	Undetected	No concerns	Major concerns	No concerns	No concerns	Low
COM-M:RT-M	0	Some concerns	Undetected	No concerns	Major concerns	No concerns	No concerns	Low

AE-M, moderate-intensity aerobic exercise; AE-HI, high-intensity aerobic exercise; RT-M, moderate-intensity resistance exercise; RT-HI, high-intensity resistance exercise; COM-M, moderate-intensity combined exercise; COM-HI, high-intensity combined exercise; CON, blank controls

Table 5. Summary Grading of Evidence for Weight

Comparison	Number of studies	Within-study bias	Reporting bias	Indirectness	Imprecision	Heterogeneity	Incoherence	Confidence rating
**Mixed estimates**								
AE-M:AE-HI	3	Some concerns	Undetected	No concerns	Major concerns	No concerns	No concerns	Low
AE-M:COM-M	1	Some concerns	Undetected	No concerns	No concerns	No concerns	No concerns	Moderate
AE-M:CON	13	Some concerns	Undetected	No concerns	No concerns	No concerns	No concerns	Moderate
AE-M:RT-HI	1	Some concerns	Undetected	No concerns	Some concerns	Some concerns	No concerns	Low
AE-M:RT-M	6	Some concerns	Undetected	No concerns	Some concerns	Some concerns	No concerns	Low
AE-HI:COM-HI	2	Some concerns	Undetected	No concerns	Major concerns	No concerns	No concerns	Low
AE-HI:CON	11	Some concerns	Undetected	No concerns	No concerns	No concerns	No concerns	Moderate
AE-HI:RT-HI	1	Some concerns	Undetected	No concerns	No concerns	Major concerns	No concerns	Low
COM-HI:CON	1	Some concerns	Undetected	No concerns	No concerns	Some concerns	No concerns	Moderate
COM-HI:RT-HI	1	Some concerns	Undetected	No concerns	Some concerns	Some concerns	No concerns	Low
COM-M:CON	5	Some concerns	Undetected	No concerns	No concerns	No concerns	No concerns	Moderate
COM-M:RT-M	1	Some concerns	Undetected	No concerns	No concerns	No concerns	No concerns	Moderate
CON:RT-HI	1	Major concerns	Undetected	No concerns	Major concerns	No concerns	No concerns	Very low
CON:RT-M	4	Some concerns	Undetected	No concerns	No concerns	Major concerns	No concerns	Low
RT-HI:RT-M	1	Some concerns	Undetected	No concerns	Major concerns	No concerns	No concerns	Low
**Indirect estimates**								
AE-M:COM-HI	0	Some concerns	Undetected	No concerns	Major concerns	No concerns	No concerns	Low
AE-HI:COM-M	0	Some concerns	Undetected	No concerns	No concerns	No concerns	No concerns	Moderate
AE-HI:RT-M	0	Some concerns	Undetected	No concerns	Some concerns	Some concerns	No concerns	Low
COM-HI:COM-M	0	Some concerns	Undetected	No concerns	Major concerns	No concerns	No concerns	Low
COM-HI:RT-M	0	Some concerns	Undetected	No concerns	Major concerns	No concerns	No concerns	Low
COM-M:RT-HI	0	Some concerns	Undetected	No concerns	No concerns	No concerns	No concerns	Moderate

AE-M, moderate-intensity aerobic exercise; AE-HI, high-intensity aerobic exercise; RT-M, moderate-intensity resistance exercise; RT-HI, high-intensity resistance exercise; COM-M, moderate-intensity combined exercise; COM-HI, high-intensity combined exercise; CON, blank controls

Table S5 Estimated Global Inconsistency in Networks

Outcome	Chi square	Prob > chi2
Body fat percentage	14.5	0.5215
Body mass index	9.89	0.8264
Fat mass	0.52	1.0000
Fat free mass	5.80	0.8316
Weight	11.53	0.5666

Table S6 Estimated Local Inconsistency for Each Pairwise Comparison

Table 1. Body Fat Percentage

Side	Direct		Indirect		Difference			tau
	Coef.	Std. Err.	Coef.	Std. Err.	Coef.	Std. Err.	P>z	
CON: AE-M	-2.35173	0.421119	-0.08526	1.162597	-2.26648	1.235415	0.067	1.300767
CON: AE-HI	-2.02221	0.451098	-1.18455	1.323209	-0.83767	1.387555	0.546	1.361175
CON: RT-M	-0.69933	0.759844	-4.37753	1.182662	3.678199	1.397583	0.008	1.225726
CON: RT-HI	-1.59378	0.973237	-2.5821	1.139836	0.988323	1.498301	0.509	1.354455
CON: COM-M *	-2.34682	0.707334	-4.19255	2.207597	1.845727	2.316342	0.426	1.354547
CON: COM-HI	-1.99769	1.024119	-2.45571	1.21953	0.458017	1.588095	0.773	1.369029
AE-M: AE-HI	0.091209	0.891954	0.170559	0.702353	-0.07935	1.134815	0.944	1.370517
AE-M: RT-M	-0.62734	0.848233	2.285881	1.217134	-2.91322	1.475874	0.048	1.289446
AE-M: RT-HI	-3.12384	1.935688	0.680506	0.850384	-3.80434	2.098973	0.07	1.297322
AE-M: COM-M	-0.10034	1.413058	-0.57692	0.90818	0.476574	1.681497	0.777	1.371901
AE-HI: RT-HI	-0.37223	1.083319	0.24104	1.10328	-0.61327	1.541797	0.691	1.363753
AE-HI: COM-HI *	-0.17413	0.854569	-0.58847	1.853949	0.414337	2.041242	0.839	1.372125
RT-M: RT-HI	1.927304	2.047855	-0.8208	1.084526	2.748105	2.313772	0.235	1.333941
RT-M: COM-M	-0.77243	1.435701	-0.68604	1.192047	-0.08639	1.866863	0.963	1.372273
RT-HI: COM-HI	-0.28369	1.058161	0.117202	1.740928	-0.4009	2.039707	0.844	1.369847

AE-M, moderate-intensity aerobic exercise; AE-HI, high-intensity aerobic exercise; RT-M, moderate-intensity resistance exercise; RT-HI, high-intensity resistance exercise; COM-M, moderate-intensity combined exercise; COM-HI, high-intensity combined exercise; CON, blank controls

Table 2. Body Mass Index

Side	Direct		Indirect		Difference			tau
	Coef.	Std. Err.	Coef.	Std. Err.	Coef.	Std. Err.	P>z	
CON:AE-M	-1.32831	0.261571	-0.85104	0.62296	-0.47727	0.675918	0.48	0.814797
CON:AE-HI	-0.8666	0.290233	-1.10441	0.757253	0.237812	0.812621	0.77	0.820207
CON: RT-M	-0.75124	0.465018	-0.84582	0.525128	0.094574	0.70312	0.893	0.822126
CON: RT-HI	-0.27208	0.926053	-0.63233	0.60506	0.360252	1.106197	0.745	0.82081
CON:COM-M *	-1.10963	0.382056	-1.96064	1.056076	0.851007	1.122916	0.449	0.8117
CON:COM-HI	-1.13114	0.908431	-1.27303	0.786983	0.141894	1.204458	0.906	0.822414
AE-M:AE-HI	0.336939	0.629805	0.369047	0.399744	-0.03211	0.746216	0.966	0.823084
AE-M: RT-M	0.257921	0.360518	1.342805	0.74348	-1.08488	0.825435	0.189	0.796037
AE-M: RT-HI	0.364349	0.909271	0.914736	0.639142	-0.55039	1.112366	0.621	0.818643
AE-M:COM-M	-0.35811	0.895203	0.157952	0.466627	-0.51607	1.009206	0.609	0.816649
AE-HI: RT-HI	-0.07224	0.913173	0.596645	0.647813	-0.66889	1.119753	0.55	0.816783
AE-HI:COM-M	0.3215	0.879836	-0.50635	0.488622	0.827849	1.006693	0.411	0.81282
AE-HI:COM-HI *	-0.26618	0.642674	-0.53492	1.367166	0.268744	1.509161	0.859	0.821603
RT-M: RT-HI	0.523434	0.902674	0.110416	0.709891	0.413018	1.1478	0.719	0.820296
RT-M:COM-M	-0.75725	0.895796	-0.28521	0.556817	-0.47204	1.054723	0.654	0.817445
RT-HI:COM-HI	-0.8708	0.919949	-0.47172	0.997945	-0.39908	1.356996	0.769	0.820877

AE-M, moderate-intensity aerobic exercise; AE-HI, high-intensity aerobic exercise; RT-M, moderate-intensity resistance exercise; RT-HI, high-intensity resistance exercise; COM-M, moderate-intensity combined exercise; COM-HI, high-intensity combined exercise; CON, blank controls

Table 3. Fat Mass

Side	Direct		Indirect		Difference			tau
	Coef.	Std. Err.	Coef.	Std. Err.	Coef.	Std. Err.	P>z	
CON:AE-M *	-2.24088	0.677287	-1.22109	2.150673	-1.01979	2.262262	0.652	1.403038
CON:AE-HI	-1.31206	0.548491	-1.95848	1.428178	0.646421	1.532895	0.673	1.397226
CON: RT-HI	-1.47481	1.018198	-0.77685	1.319917	-0.69795	1.667352	0.676	1.395886
CON:COM-M	.	.	.	.	.	.	.	.
CON:COM-HI	-1.50307	1.01843	-1.53463	1.086128	0.031554	1.489297	0.983	1.409257
AE-M:AE-HI	0.362115	1.018864	1.159382	1.049193	-0.79727	1.460073	0.585	1.398054
AE-HI:RT-HI	0.072471	1.018652	0.368772	1.328546	-0.2963	1.673859	0.859	1.407399
AE-HI:COM-HI *	-0.10021	0.721713	-0.28796	2.046105	0.187751	2.168897	0.931	1.407742
RT-HI:COM-HI	-0.60151	1.005777	0.527835	1.677185	-1.12935	1.955637	0.564	1.387617

AE-M, moderate-intensity aerobic exercise; AE-HI, high-intensity aerobic exercise; RT-M, moderate-intensity resistance exercise; RT-HI, high-intensity resistance exercise; COM-M, moderate-intensity combined exercise; COM-HI, high-intensity combined exercise; CON, blank controls

Table 4. Fat Free Mass

Side	Direct		Indirect		Difference			tau
	Coef.	Std. Err.	Coef.	Std. Err.	Coef.	Std. Err.	P>z	
CON:AE-M	0.265369	0.382258	-1.00547	1.292465	1.27084	1.346482	0.345	0.702175
CON:AE-HI	0.559948	0.379103	1.564654	1.218772	-1.00471	1.282055	0.433	0.682971
CON: RT-M	0.664728	0.528748	0.863552	1.164617	-0.19882	1.279937	0.877	0.715403
CON: RT-HI	0.93768	0.511389	1.669525	0.952141	-0.73185	1.085278	0.5	0.682372
CON:COM-M	.	.	.	.	.	.	.	.
CON:COM-HI	-0.30002	0.539143	2.181546	0.83831	-2.48157	0.99666	0.013	0.526968
AE-M:AE-HI	0.900253	1.331323	0.418887	0.540052	0.481366	1.436875	0.738	0.701622
AE-M: RT-M	0.789086	0.696427	0.139271	0.85221	0.649815	1.103124	0.556	0.718447
AE-M: RT-HI	1.984987	2.309025	0.86456	0.592642	1.120427	2.399339	0.641	0.694103
AE-HI: RT-HI *	0.43559	0.6681	0.465564	0.83106	-0.02997	1.069958	0.978	0.727853
AE-HI:COM-HI *	0.137559	0.612933	-1.47629	1.349136	1.613848	1.483432	0.277	0.660703
RT-M: RT-HI	1.402035	1.745453	0.254354	0.678645	1.147682	1.872899	0.54	0.696302
RT-HI:COM-HI *	-0.33121	0.622514	-2.61771	1.692278	2.286499	1.802539	0.205	0.675708

AE-M, moderate-intensity aerobic exercise; AE-HI, high-intensity aerobic exercise; RT-M, moderate-intensity resistance exercise; RT-HI, high-intensity resistance exercise; COM-M, moderate-intensity combined exercise; COM-HI, high-intensity combined exercise; CON, blank controls

Table 5. Weight

Side	Direct		Indirect		Difference			tau
	Coef.	Std. Err.	Coef.	Std. Err.	Coef.	Std. Err.	P>z	
CON:AE-M	-2.12443	0.399095	-1.46743	0.98347	-0.65699	1.051553	0.532	1.045156
CON:AE-HI	-2.36951	0.500954	-1.70721	1.408187	-0.6623	1.502449	0.659	1.065423
CON: RT-M	-1.46656	0.632508	-1.31043	0.826928	-0.15613	1.033198	0.88	1.060786
CON: RT-HI	-0.2	1.134591	-0.69082	1.588647	0.49082	1.952203	0.801	1.050552
CON:COM-M *	-4.67565	0.705213	-2.53743	2.697967	-2.13822	2.737292	0.435	1.030272
CON:COM-HI	-2.51897	2.630438	-3.46056	1.968582	0.941589	3.374876	0.78	1.038666
AE-M:AE-HI	-0.61506	0.807062	0.03027	0.719679	-0.64533	1.0876	0.553	1.035634
AE-M: RT-M	0.426282	0.531829	1.511815	1.112344	-1.08553	1.231561	0.378	1.056602
AE-M: RT-HI	2.080222	2.583348	1.600846	1.045835	0.479376	2.798309	0.864	1.041373
AE-M:COM-M	-1.2707	1.897375	-2.82019	0.86278	1.549484	2.110663	0.463	1.021435
AE-HI: RT-HI	-0.01761	2.298787	2.363163	1.098734	-2.38077	2.54623	0.35	1.041045
AE-HI:COM-HI*	-0.94586	1.640407	-0.25936	3.391723	-0.68651	3.668641	0.852	1.043584
RT-M: RT-HI	1.681073	2.7945	0.937492	1.105423	0.743581	3.03166	0.806	1.041138
RT-M:COM-M	-1.78721	1.931796	-3.50631	0.932114	1.719098	2.168551	0.428	1.022947
RT-HI:COM-HI	-2.94059	2.452869	-2.59924	2.220702	-0.34135	3.283168	0.917	1.042785

AE-M, moderate-intensity aerobic exercise; AE-HI, high-intensity aerobic exercise; RT-M, moderate-intensity resistance exercise; RT-HI, high-intensity resistance exercise; COM-M, moderate-intensity combined exercise; COM-HI, high-intensity combined exercise; CON, blank controls

Figure S1 Summary of The Risk of Bias Assessment

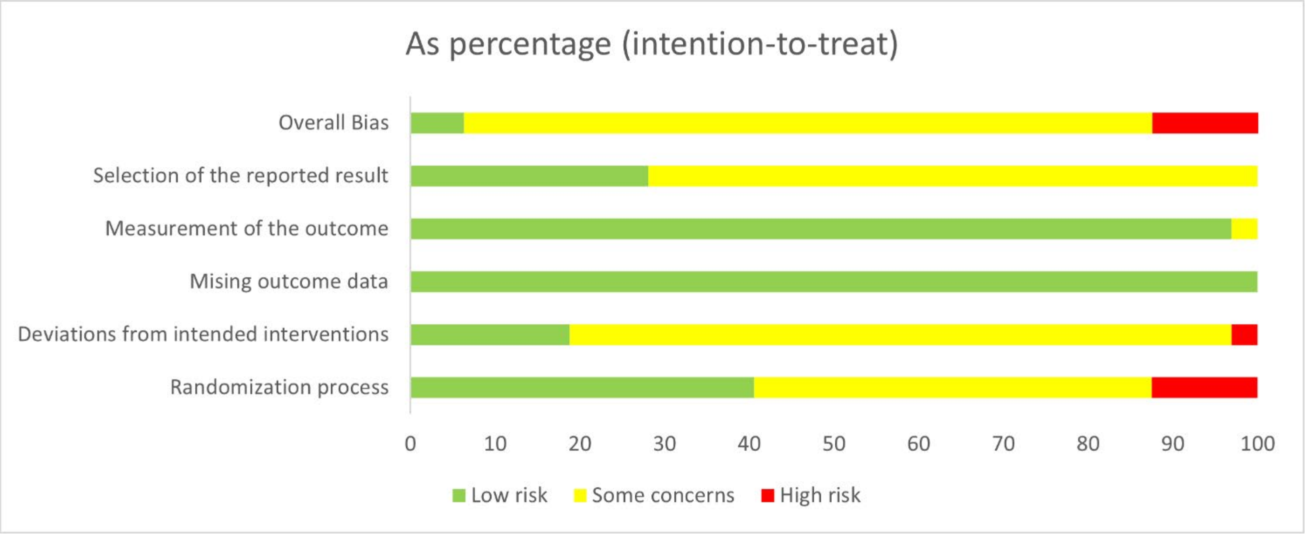

Figure S2 Confidence in Network Meta-analysis (CINeMA) Report

Confidence in Network Meta-analysis (CINeMA) Report for Body Fat Percentage

The colors in the circles indicate the percentage of low RoB studies [green], moderate RoB studies [yellow] and high RoB studies [red] involving each intervention.

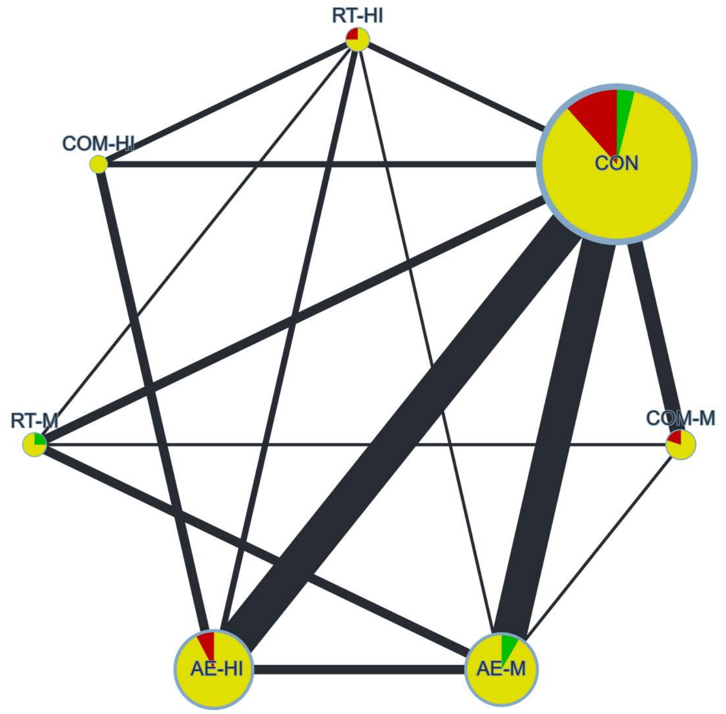

Figure 1. Summary of study limitations of the included studies

AE-M, moderate-intensity aerobic exercise
AE-HI, high-intensity aerobic exercise
RT-M, moderate-intensity resistance exercise
RT-HI, high-intensity resistance exercise
COM-M, moderate-intensity combined exercise
COM-HI, high-intensity combined exercise
CON, blank controls

Based on the above assessment of RoB for each comparison and the contribution matrix detailing contribution of each direct comparison to all network estimates, the following bar graphs show the percentage of low or moderate RoB contributions for each network estimate. The judgements about study limitations in each direct comparison is shown at the beginning of the graph. Each bar corresponds to a NMA relative treatment effect and shows how much information comes from comparisons at moderate risk of bias [yellow].

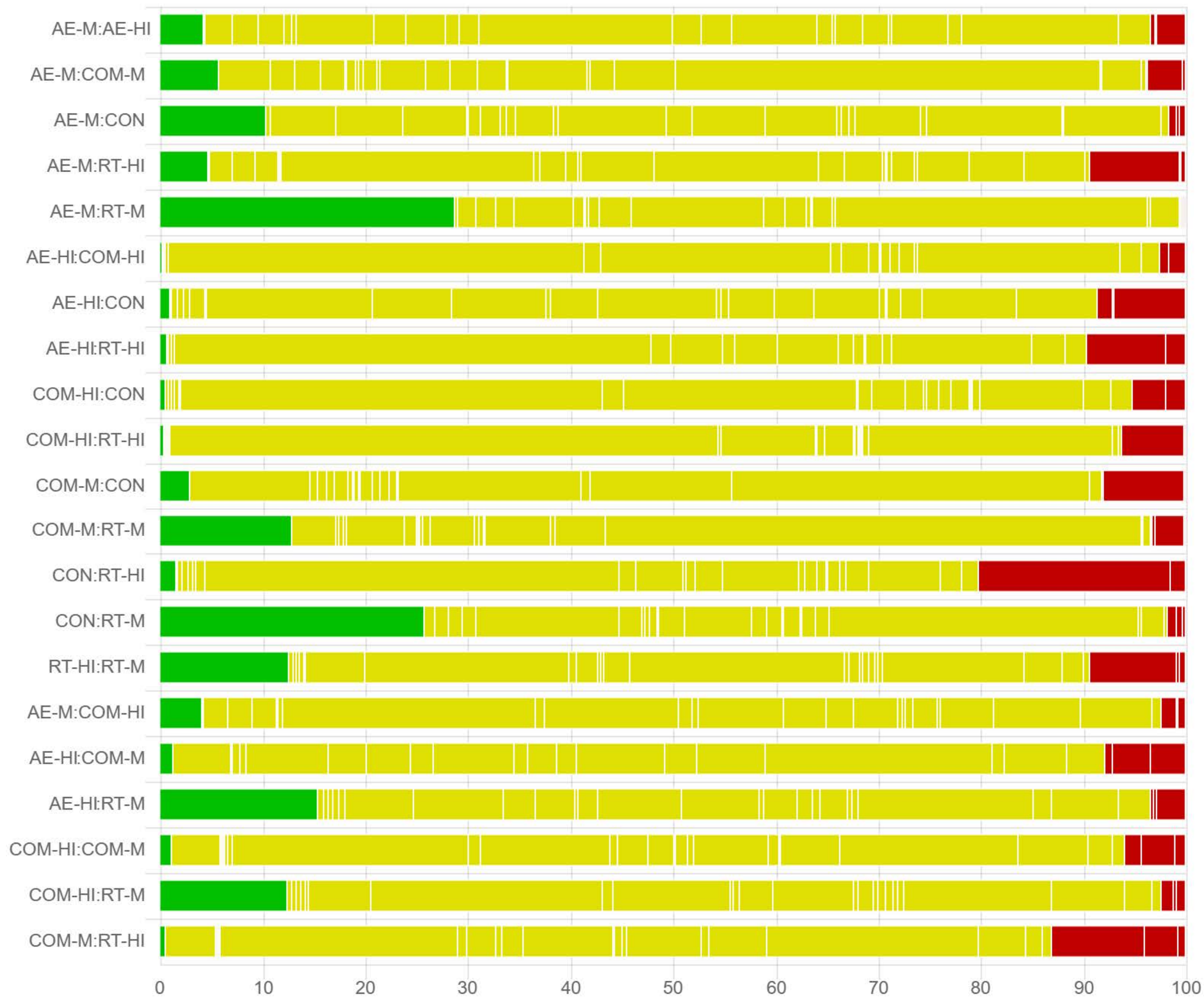

Figure 2. Contribution of low or moderate RoB comparisons to each network estimate

AE-M, moderate-intensity aerobic exercise
AE-HI, high-intensity aerobic exercise
RT-M, moderate-intensity resistance exercise
RT-HI, high-intensity resistance exercise
COM-M, moderate-intensity combined exercise
COM-HI, high-intensity combined exercise
CON, blank controls

Confidence in Network Meta-analysis (CINeMA) Report for Body Mass Index

The colors in the circles indicate the percentage of low RoB studies [green], moderate RoB studies [yellow] and high RoB studies [red] involving each intervention.

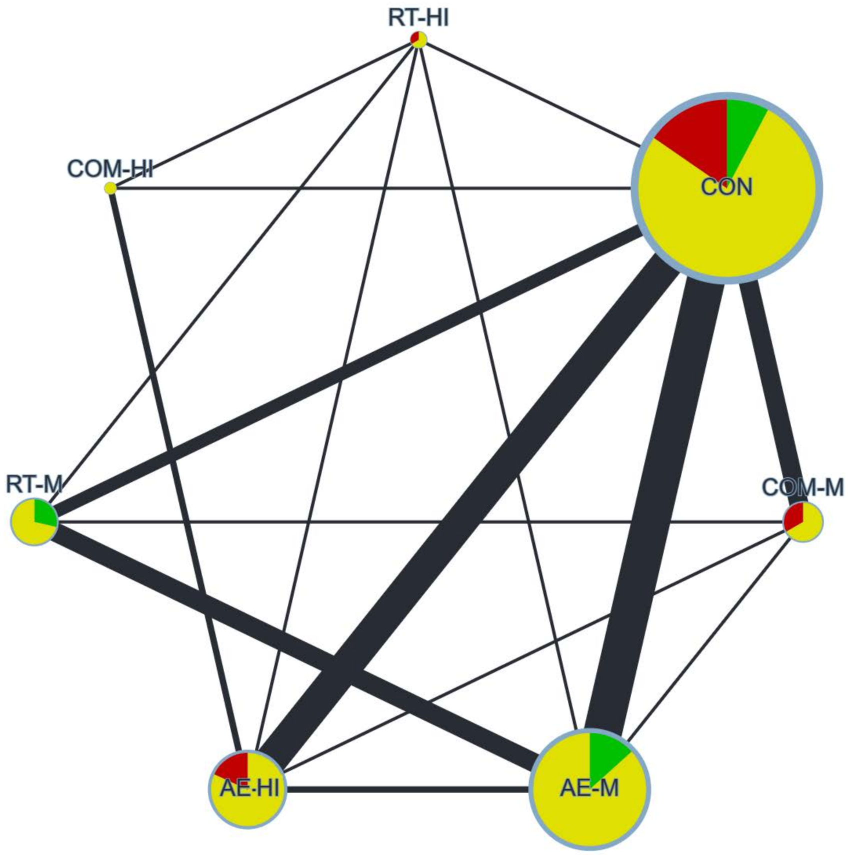

Figure 3. Summary of study limitations of the included studies

AE-M, moderate-intensity aerobic exercise
AE-HI, high-intensity aerobic exercise
RT-M, moderate-intensity resistance exercise
RT-HI, high-intensity resistance exercise
COM-M, moderate-intensity combined exercise
COM-HI, high-intensity combined exercise
CON, blank controls

Based on the above assessment of RoB for each comparison and the contribution matrix detailing contribution of each direct comparison to all network estimates, the following bar graphs show the percentage of low or moderate RoB contributions for each network estimate. The judgements about study limitations in each direct comparison is shown at the beginning of the graph. Each bar corresponds to a NMA relative treatment effect and shows how much information comes from comparisons at moderate risk of bias [yellow].

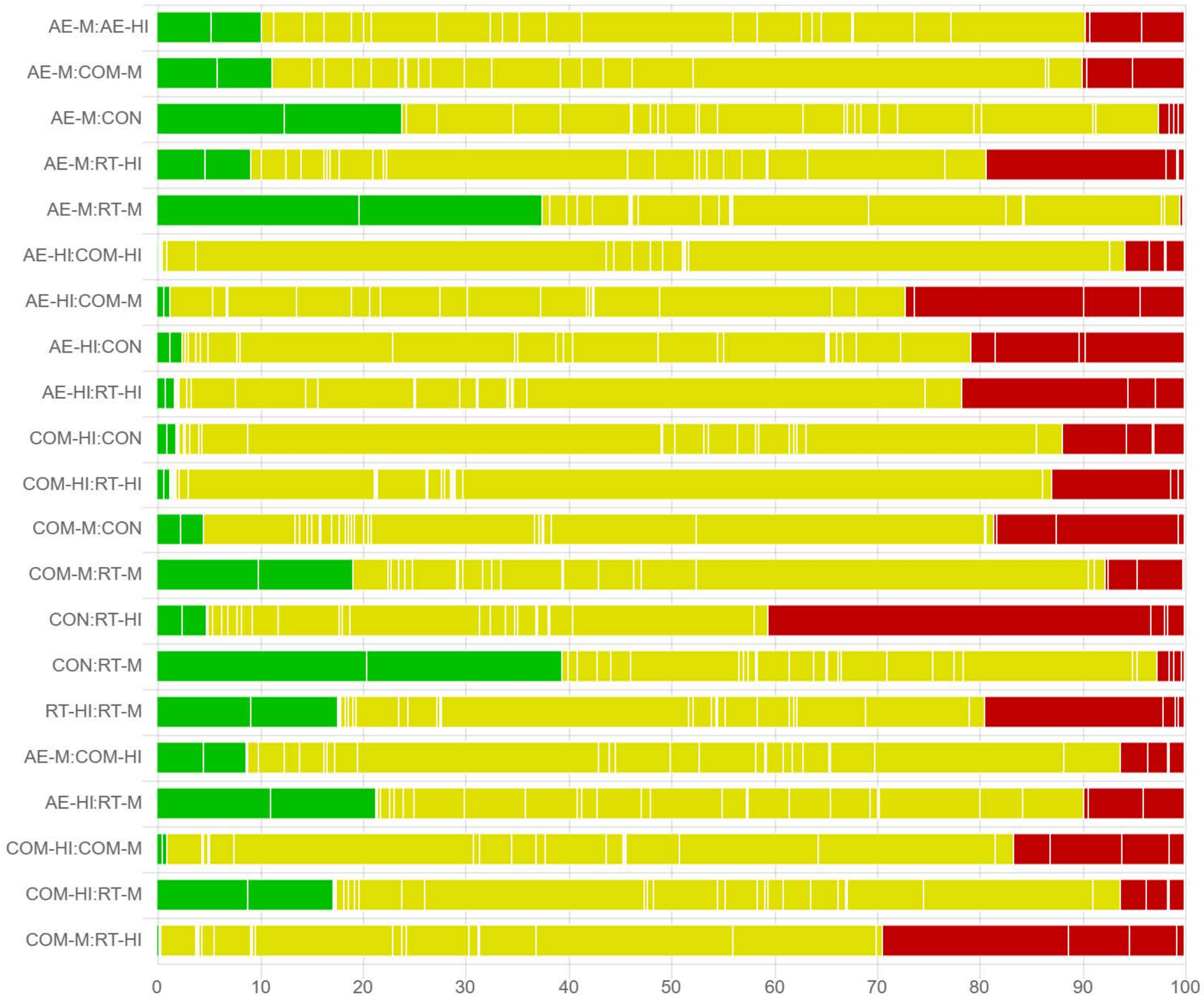

Figure 4. Contribution of low or moderate RoB comparisons to each network estimate

AE-M, moderate-intensity aerobic exercise
AE-HI, high-intensity aerobic exercise
RT-M, moderate-intensity resistance exercise
RT-HI, high-intensity resistance exercise
COM-M, moderate-intensity combined exercise
COM-HI, high-intensity combined exercise
CON, blank controls

Confidence in Network Meta-analysis (CINeMA) Report for Fat Mass

The colors in the circles indicate the percentage of low RoB studies [green], moderate RoB studies [yellow] and high RoB studies [red] involving each intervention.

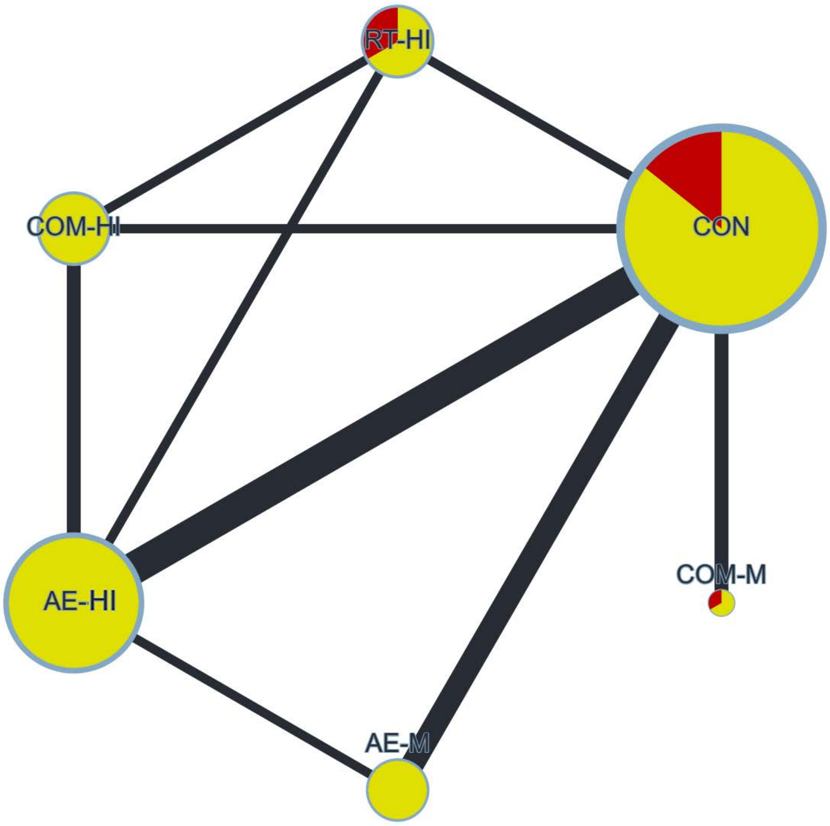

Figure 5. Summary of study limitations of the included studies

AE-M, moderate-intensity aerobic exercise
AE-HI, high-intensity aerobic exercise
RT-HI, high-intensity resistance exercise
COM-M, moderate-intensity combined exercise
COM-HI, high-intensity combined exercise
CON, blank controls

Based on the above assessment of RoB for each comparison and the contribution matrix detailing contribution of each direct comparison to all network estimates, the following bar graphs show the percentage of low or moderate RoB contributions for each network estimate. The judgements about study limitations in each direct comparison is shown at the beginning of the graph. Each bar corresponds to a NMA relative treatment effect and shows how much information comes from comparisons at moderate risk of bias [yellow].

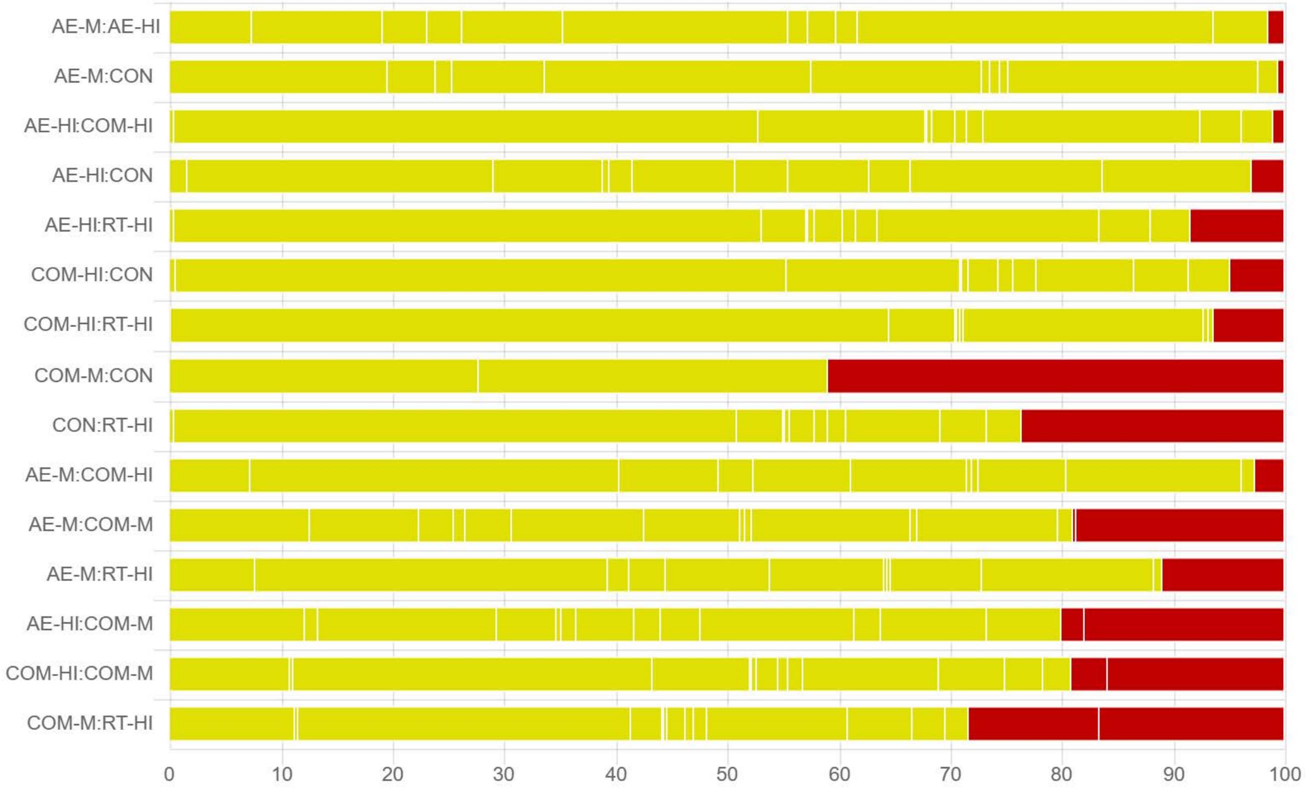

Figure 6. Contribution of low or moderate RoB comparisons to each network estimate

AE-M, moderate-intensity aerobic exercise
AE-HI, high-intensity aerobic exercise
RT-HI, high-intensity resistance exercise
COM-M, moderate-intensity combined exercise
COM-HI, high-intensity combined exercise
CON, blank controls

Confidence in Network Meta-analysis (CINeMA) Report for Fat-Free Mass

The colors in the circles indicate the percentage of low RoB studies [green], moderate RoB studies [yellow] and high RoB studies [red] involving each intervention.

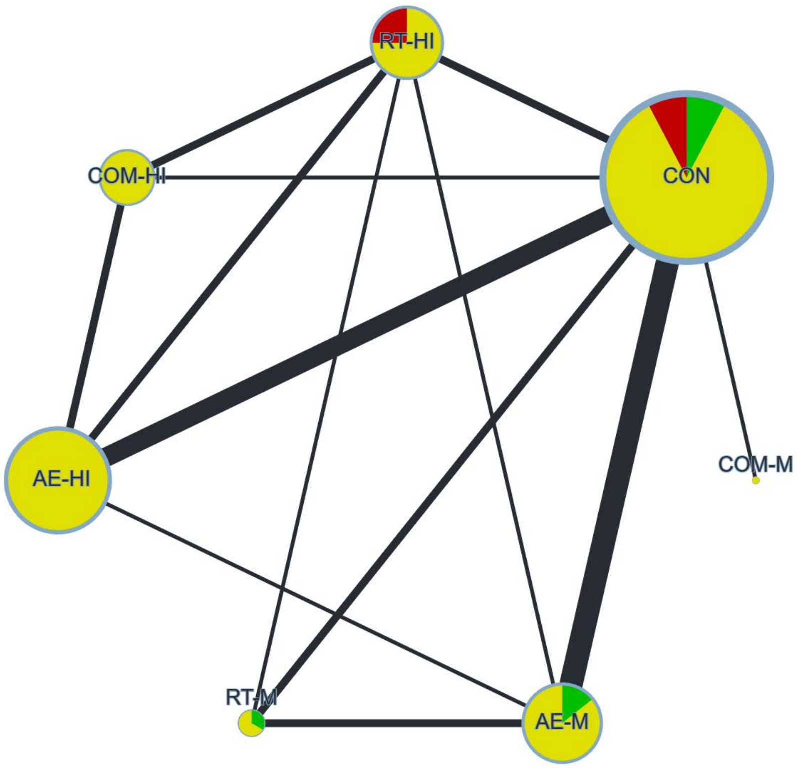

Figure 7. Summary of study limitations of the included studies

AE-M, moderate-intensity aerobic exercise
AE-HI, high-intensity aerobic exercise
RT-M, moderate-intensity resistance exercise
RT-HI, high-intensity resistance exercise
COM-M, moderate-intensity combined exercise
COM-HI, high-intensity combined exercise
CON, blank controls

Based on the above assessment of RoB for each comparison and the contribution matrix detailing contribution of each direct comparison to all network estimates, the following bar graphs show the percentage of low or moderate RoB contributions for each network estimate. The judgements about study limitations in each direct comparison is shown at the beginning of the graph. Each bar corresponds to a NMA relative treatment effect and shows how much information comes from comparisons at moderate risk of bias [yellow].

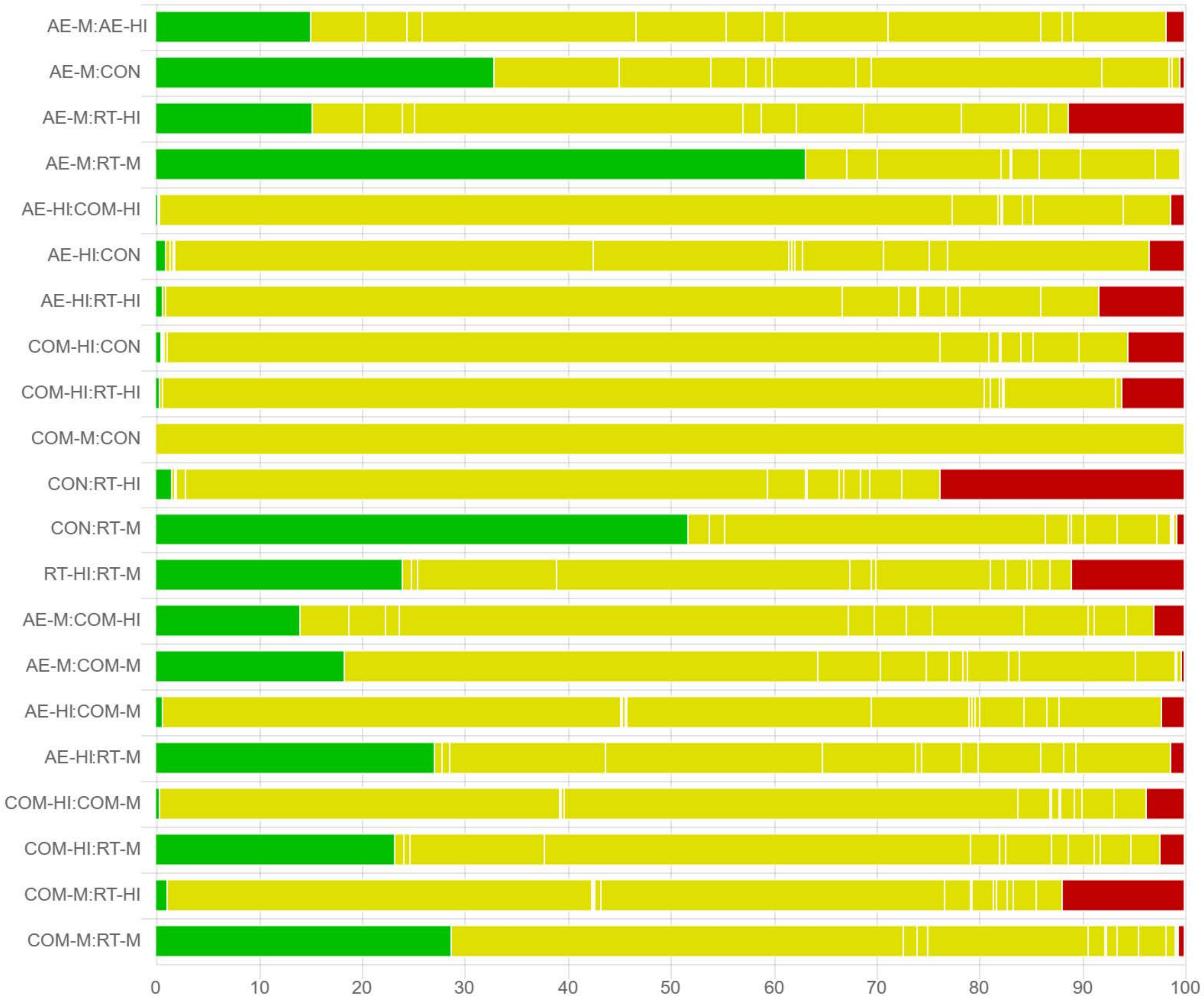

Figure 8. Contribution of low or moderate RoB comparisons to each network estimate

AE-M, moderate-intensity aerobic exercise
AE-HI, high-intensity aerobic exercise
RT-M, moderate-intensity resistance exercise
RT-HI, high-intensity resistance exercise
COM-M, moderate-intensity combined exercise
COM-HI, high-intensity combined exercise
CON, blank controls

Confidence in Network Meta-analysis (CINeMA) Report for Weight

The colors in the circles indicate the percentage of low RoB studies [green], moderate RoB studies [yellow] and high RoB studies [red] involving each intervention.

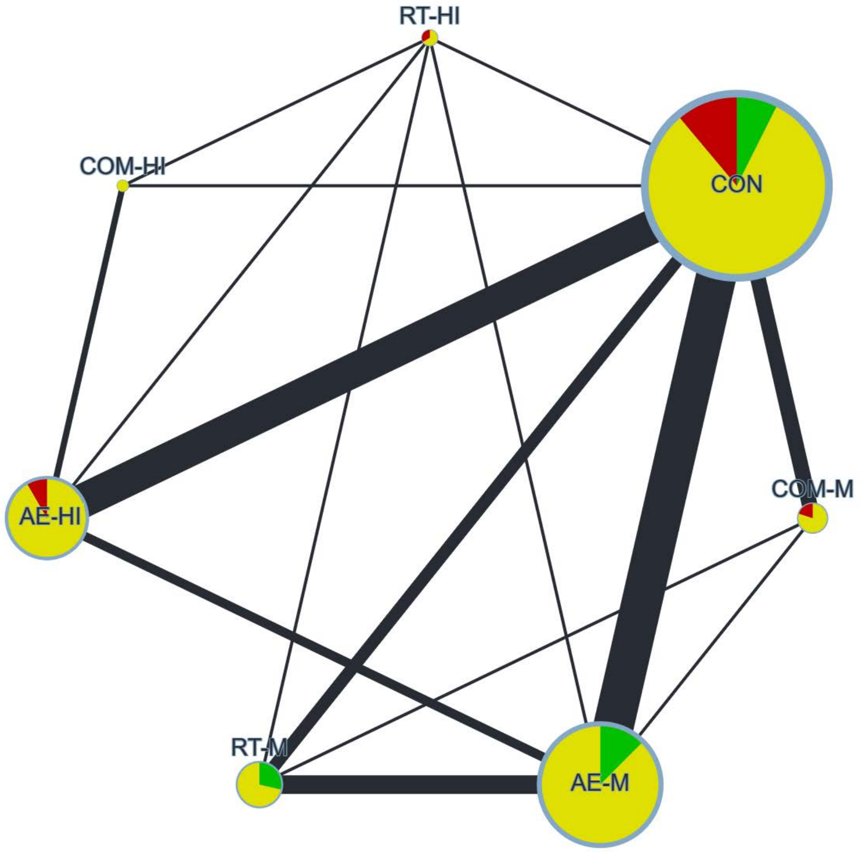

Figure 9. Summary of study limitations of the included studies

AE-M, moderate-intensity aerobic exercise
AE-HI, high-intensity aerobic exercise
RT-M, moderate-intensity resistance exercise
RT-HI, high-intensity resistance exercise
COM-M, moderate-intensity combined exercise
COM-HI, high-intensity combined exercise
CON, blank controls

Based on the above assessment of RoB for each comparison and the contribution matrix detailing contribution of each direct comparison to all network estimates, the following bar graphs show the percentage of low or moderate RoB contributions for each network estimate. The judgements about study limitations in each direct comparison is shown at the beginning of the graph. Each bar corresponds to a NMA relative treatment effect and shows how much information comes from comparisons at moderate risk of bias [yellow].

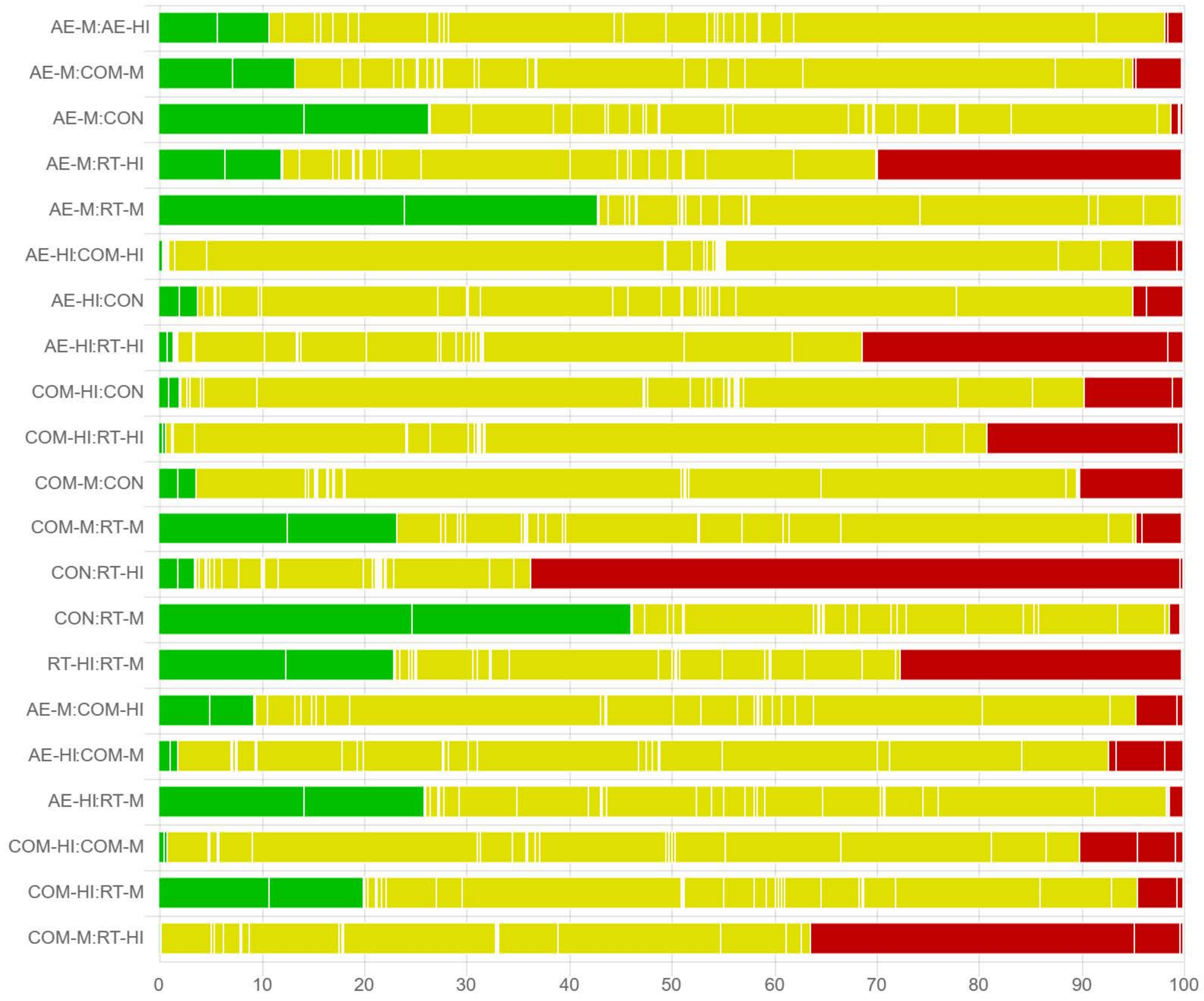

Figure 10. Contribution of low or moderate RoB comparisons to each network estimate

AE-M, moderate-intensity aerobic exercise
AE-HI, high-intensity aerobic exercise
RT-M, moderate-intensity resistance exercise
RT-HI, high-intensity resistance exercise
COM-M, moderate-intensity combined exercise
COM-HI, high-intensity combined exercise
CON, blank controls

Figure S3 Network Forest

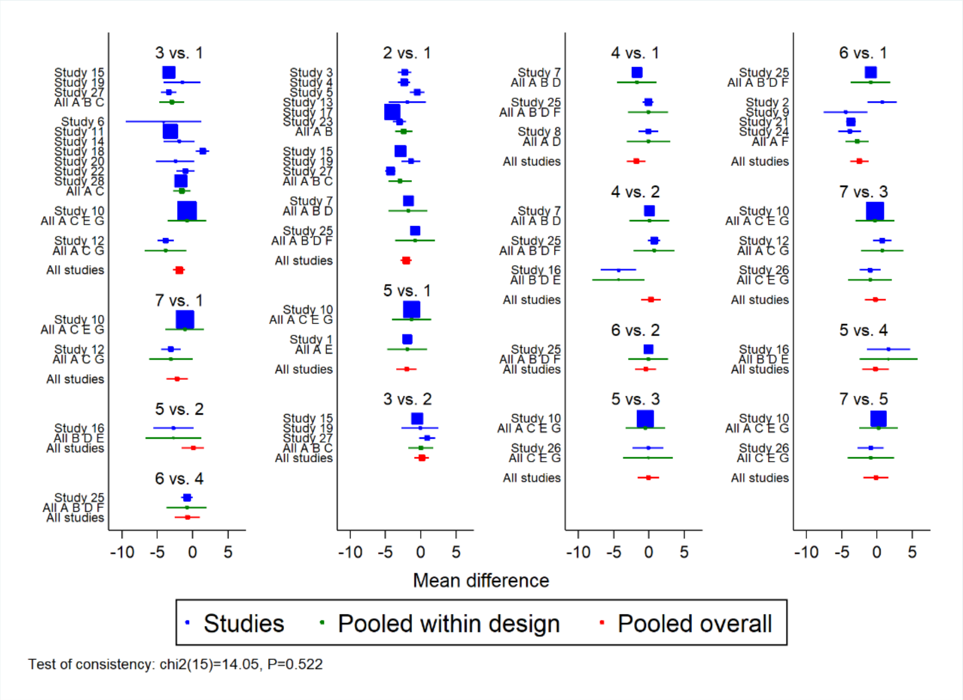

Figure1 Network Forest for Body Fat Percentage

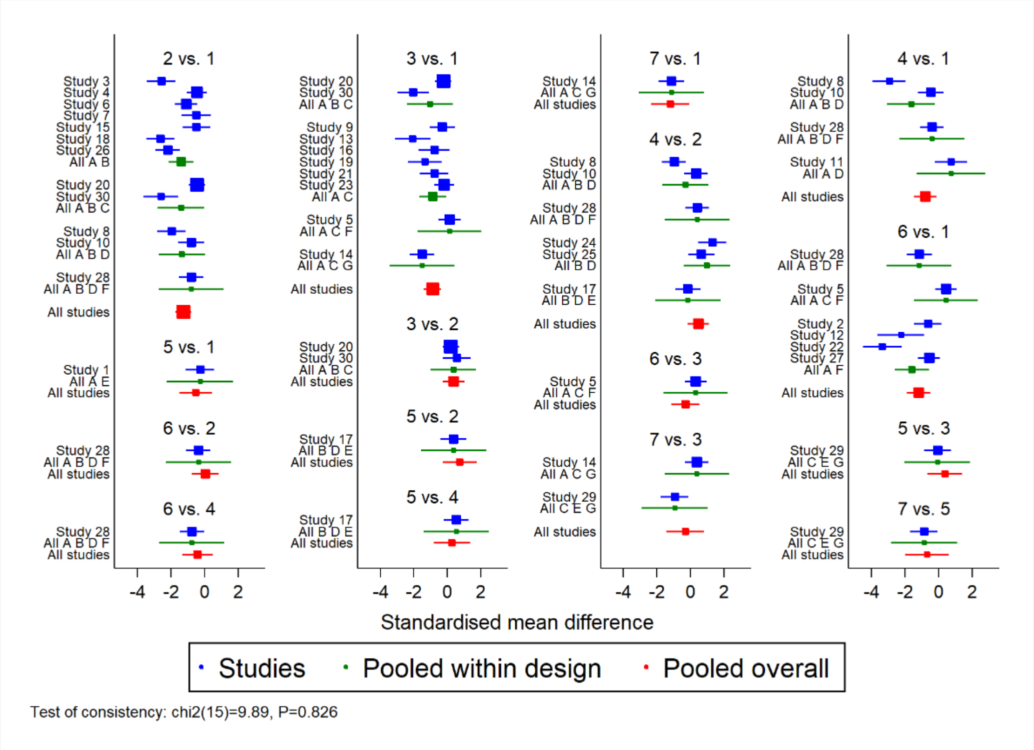

Figure2 Network Forest for Body Mass Index

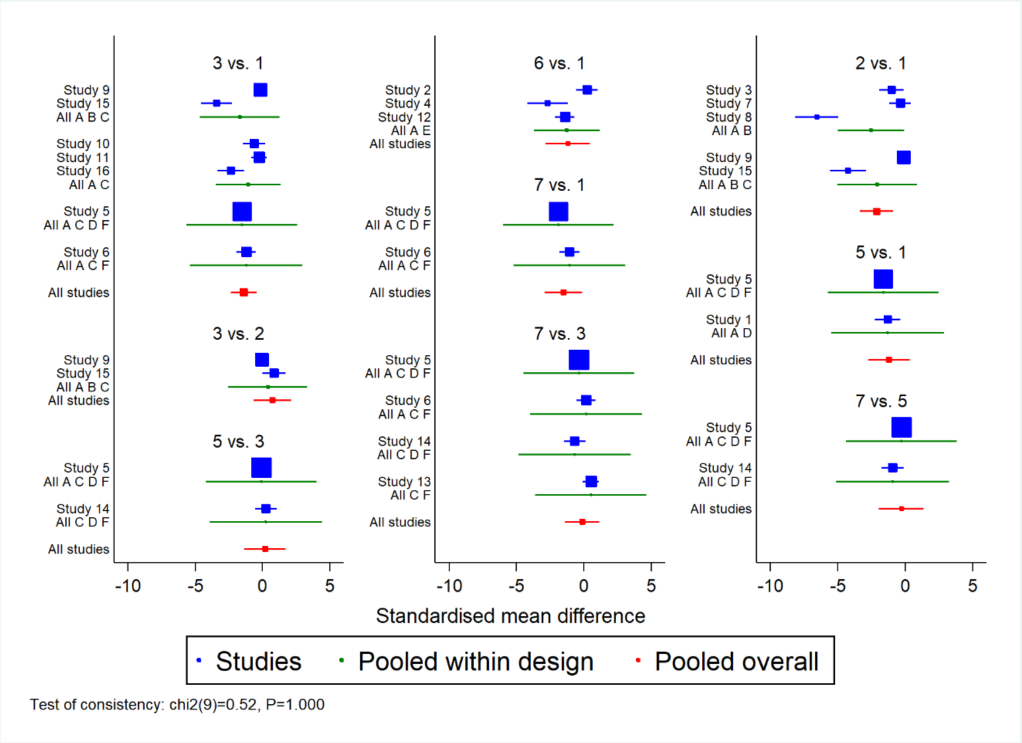

Figure3 Network Forest for Fat Mass

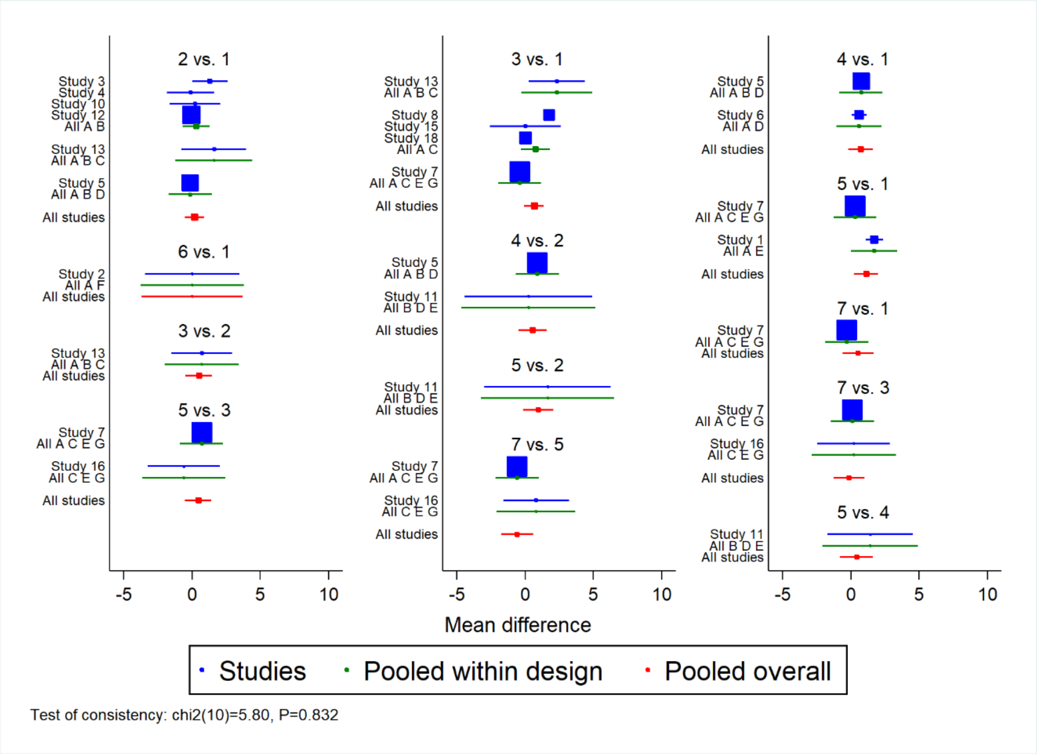

Figure4 Network Forest for Fat Free Mass

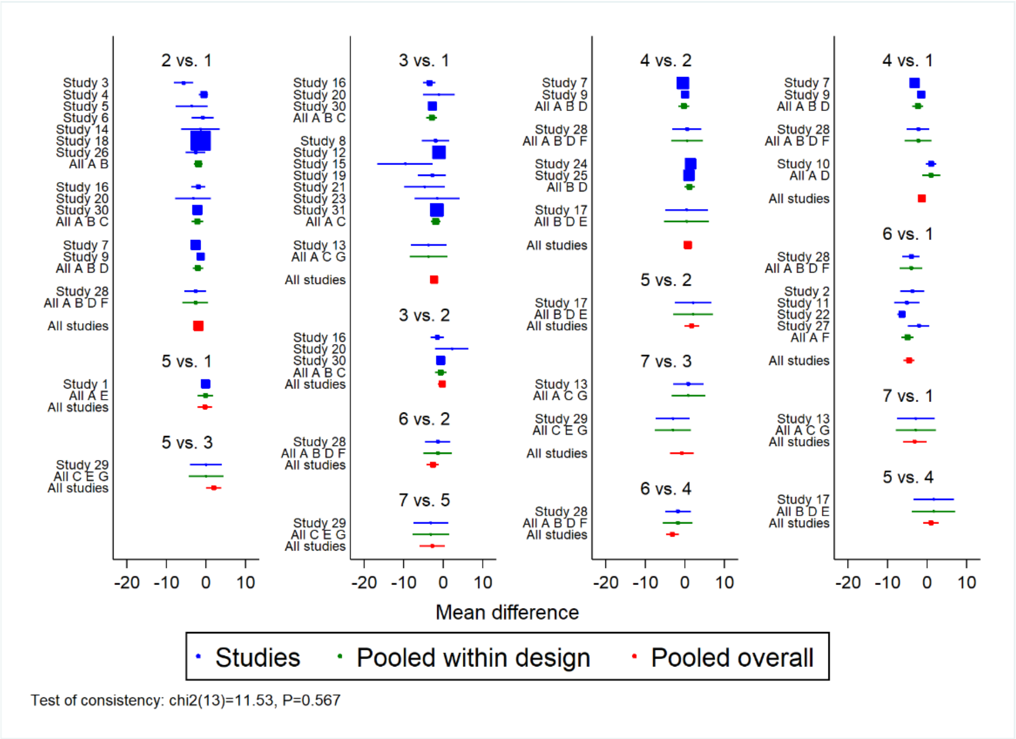

Figure5 Network Forest for Weight

Figure S4 Pairwise Meta-Analysis Forest Plot

Figure 1. Pairwise Meta-Analyses for Body Fat Percentage

AE-M, moderate-intensity aerobic exercise; AE-HI, high-intensity aerobic exercise; RT-M, moderate-intensity resistance exercise; RT-HI, high-intensity resistance exercise; COM-M, moderate-intensity combined exercise; COM-HI, high-intensity combined exercise; CON, blank controls

	AE-M			CON					Weight	Weight
**Study**	**Total**	**Mean**	**SD**	**Total**	**Mean**	**SD**	**MD**	**95%−CI**	**(common)**	**(random)**
Farpour−Lambert (2009)	22	−1.50	1.7000	22	0.80	1.6000	−2.30	[−3.28; −1.32]	4.3%	9.1%
Saygin (2011)	20	−1.71	1.7834	19	0.66	0.9700	−2.37	[−3.27; −1.47]	5.1%	9.3%
Song (2011)	12	−1.00	1.4526	10	−0.50	1.0149	−0.50	[−1.53; 0.53]	3.8%	9.0%
Lee (2013)	16	−1.70	0.8500	12	0.10	0.6600	−1.80	[−2.36; −1.24]	13.1%	10.0%
Tan (2015)	11	−1.80	3.6642	13	0.10	2.6769	−1.90	[−4.51; 0.71]	0.6%	5.1%
Racil (2016)	16	−3.40	0.7376	14	−0.50	0.6465	−2.90	[−3.40; −2.40]	16.7%	10.1%
Tan (2016)	21	−2.04	0.5100	21	2.05	0.6800	−4.09	[−4.45; −3.73]	31.1%	10.3%
Dias (2017)	32	−1.20	2.6153	34	0.20	2.8443	−1.40	[−2.72; −0.08]	2.4%	8.3%
Kim (2019)	24	−3.00	1.4227	24	0.00	1.8138	−3.00	[−3.92; −2.08]	4.8%	9.3%
Said (2021)	15	−2.90	1.0385	15	−2.08	0.6369	−0.82	[−1.44; −0.20]	10.8%	9.9%
Cao (2022)	11	−3.10	0.9220	13	1.20	0.9602	−4.30	[−5.05; −3.55]	7.2%	9.6%
**Common effect model**	**200**			**197**			**−2.82**	**[−3.02; −2.62]**	**100.0%**	**−−**
**Random effects model**							**−2.36**	**[−3.18; −1.53]**	**−−**	**100.0%**

Heterogeneity: $I^2 = 93\%$, $\tau^2 = 1.6869$, $p < 0.01$

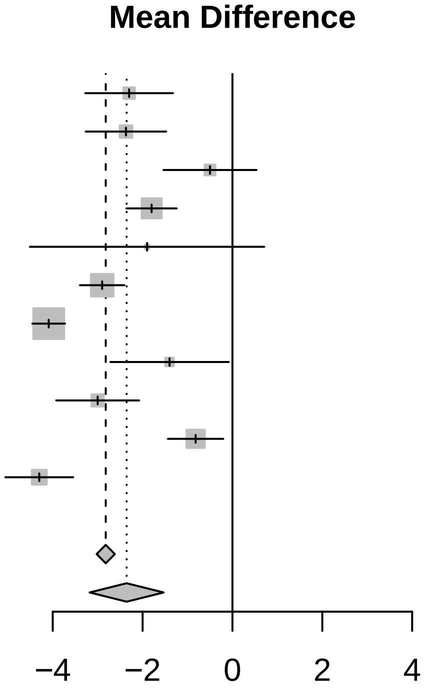

Study	AE-HI Total	AE-HI Mean	AE-HI SD	CON Total	CON Mean	CON SD	MD	95%−CI	Weight (common)	Weight (random)
Regaieg(2012)	14	−4.30	6.7089	14	−0.20	7.6922	−4.10	[−9.45; 1.25]	0.1%	2.3%
Sigal (2014)	75	−1.10	0.8989	76	−0.30	0.8989	−0.80	[−1.09; −0.51]	37.7%	10.7%
Youssef (2015)	14	−1.30	0.4000	9	1.90	0.5000	−3.20	[−3.59; −2.81]	20.6%	10.6%
Monteiro (2015)	18	−3.59	1.6325	16	0.21	1.7176	−3.80	[−4.93; −2.67]	2.4%	9.3%
Vas1cellos (2015)	10	−2.20	2.7280	10	−0.30	2.3660	−1.90	[−4.14; 0.34]	0.6%	6.6%
Racil (2016)	17	−3.90	0.6708	14	−0.50	0.6465	−3.40	[−3.87; −2.93]	14.3%	10.5%
Khammassi (2018)	10	1.60	1.3000	10	0.20	0.8331	1.40	[0.44; 2.36]	3.4%	9.7%
Dias (2017)	33	−1.30	7.2021	34	0.20	2.8443	−1.50	[−4.14; 1.14]	0.4%	5.8%
Cvetkovic (2018)	10	−2.78	3.0742	14	−0.34	3.6675	−2.44	[−5.15; 0.27]	0.4%	5.6%
Martín−García (2017)	19	−1.20	2.1794	28	−0.20	2.3499	−1.00	[−2.31; 0.31]	1.8%	8.9%
Cao (2022)	12	−2.20	1.7692	13	1.20	0.9602	−3.40	[−4.53; −2.27]	2.4%	9.3%
Salus (2022)	14	−1.20	0.6000	14	0.50	0.6000	−1.70	[−2.14; −1.26]	15.7%	10.6%
**Common effect model**	**246**			**252**			**−1.89**	**[−2.07; −1.72]**	**100.0%**	**−−**
**Random effects model**							**−2.03**	**[−2.96; −1.11]**	**−−**	**100.0%**

Heterogeneity: $I^2 = 95\%$, $\tau^2 = 2.0557$, $p < 0.01$

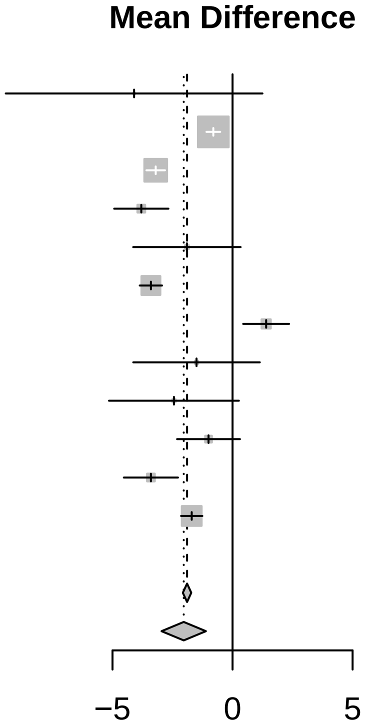

Study	RT-M Total	RT-M Mean	RT-M SD	CON Total	CON Mean	CON SD	MD	95%−CI	Weight (common)	Weight (random)
Lee (2013)	16	−1.63	0.7800	12	0.10	0.6600	−1.73	[−2.26; −1.20]	62.4%	37.9%
Alberga (2013)	12	−0.60	1.3000	7	−0.53	1.6000	−0.07	[−1.46; 1.32]	9.2%	27.1%
Said (2021)	16	−2.19	1.4770	15	−2.08	0.6369	−0.11	[−0.90; 0.68]	28.4%	35.0%
**Common effect model**	**44**			**34**			**−1.12**	**[−1.54; −0.70]**	**100.0%**	**−−**
**Random effects model**							**−0.71**	**[−1.97; 0.55]**	**−−**	**100.0%**

Heterogeneity: $I^2 = 85\%$, $\tau^2 = 1.0175$, $p < 0.01$

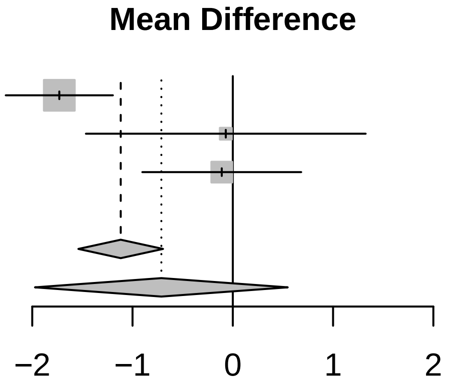

Study	RT-HI Total	RT-HI Mean	RT-HI SD	CON Total	CON Mean	CON SD	MD	95%−CI	Weight (common)	Weight (random)
SHAIBI (2006)	11	−2.50	0.8000	11	−0.60	0.6000	−1.90	[−2.49; −1.31]	24.6%	41.5%
Sigal (2014)	78	−1.60	1.2166	76	−0.30	0.8989	−1.30	[−1.64; −0.96]	75.4%	58.5%
**Common effect model**	**89**			**87**			**−1.45**	**[−1.74; −1.15]**	**100.0%**	**−−**
**Random effects model**							**−1.55**	**[−2.13; −0.97]**	**−−**	**100.0%**

Heterogeneity: $I^2 = 67\%$, $\tau^2 = 0.1197$, $p = 0.08$

Mean Difference

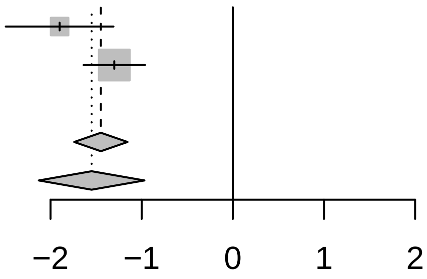

Study	COM-M Total	COM-M Mean	COM-M SD	CON Total	CON Mean	CON SD	MD	95%−CI	Weight (common)	Weight (random)
Wong (2008)	12	−0.80	3.1812	12	−1.50	1.7905	0.70	[−1.37; 2.77]	3.6%	18.4%
Jeon (2013)	8	−5.17	3.7926	7	−0.70	2.2989	−4.47	[−7.60; −1.34]	1.6%	14.0%
Wong (2017)	15	−3.60	1.0030	15	0.10	0.9839	−3.70	[−4.41; −2.99]	30.7%	23.4%
Duft (2020)	18	−2.54	2.4776	19	1.33	2.5316	−3.87	[−5.48; −2.26]	6.0%	20.3%
Said (2021)	15	−3.01	0.7993	15	−2.08	0.6369	−0.93	[−1.45; −0.41]	58.1%	23.8%
**Common effect model**	**68**			**68**			**−1.95**	**[−2.35; −1.56]**	**100.0%**	**−−**
**Random effects model**							**−2.37**	**[−4.17; −0.57]**	**−−**	**100.0%**

Heterogeneity: $I^2 = 92\%$, $\tau^2 = 3.4804$, $p < 0.01$

Mean Difference

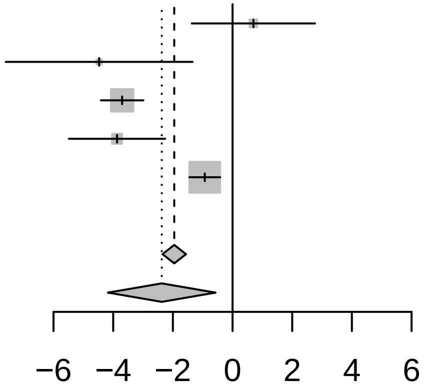

Study	COM-HI Total	COM-HI Mean	COM-HI SD	CON Total	CON Mean	CON SD	Mean Difference	MD	95%−CI	Weight (common)	Weight (random)
Sigal (2014)	75	−1.40	1.0696	76	−0.30	0.8989		−1.10	[−1.42; −0.78]	94.7%	55.7%
Monteiro (2015)	14	−2.85	1.9834	16	0.21	1.7176		−3.06	[−4.40; −1.72]	5.3%	44.3%
**Common effect model**	**89**			**92**				**−1.20**	**[−1.51; −0.90]**	**100.0%**	**−−**
**Random effects model**								**−1.97**	**[−3.88; −0.06]**	**−−**	**100.0%**

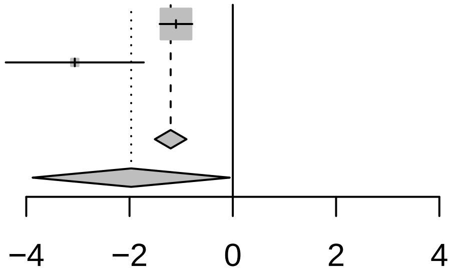

Heterogeneity: $I^2 = 87\%$, $\tau^2 = 1.6752$, $p < 0.01$

Study	AE-M Total	AE-M Mean	AE-M SD	AE-HI Total	AE-HI Mean	AE-HI SD	MD	95%−CI	Weight (common)	Weight (random)
Racil (2016)	16	−3.40	0.7376	17	−3.90	0.6708	0.50	[0.02; 0.98]	82.5%	52.4%
Dias (2017)	32	−1.20	2.6153	33	−1.30	7.2021	0.10	[−2.52; 2.72]	2.8%	12.7%
Cao (2022)	11	−3.10	0.9220	12	−2.20	1.7692	−0.90	[−2.04; 0.24]	14.7%	34.9%
**Common effect model**	**59**			**62**			**0.28**	**[−0.16; 0.72]**	**100.0%**	**−−**
**Random effects model**							**−0.04**	**[−1.09; 1.02]**	**−−**	**100.0%**

Heterogeneity: $I^2 = 59\%$, $\tau^2 = 0.4922$, $p = 0.08$

Mean Difference

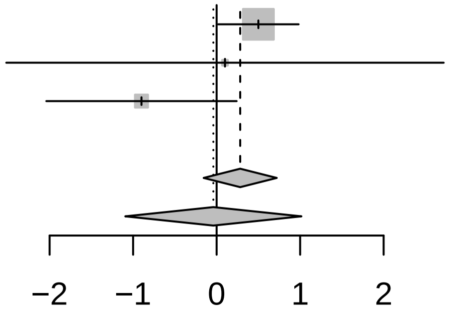

Study	AE-M Total	AE-M Mean	AE-M SD	RT-M Total	RT-M Mean	RT-M SD	MD	95%−CI	Weight (common)	Weight (random)
Lee (2013)	16	−1.70	0.8500	16	−1.63	0.7800	−0.07	[−0.64; 0.50]	69.0%	41.2%
Fiorilli (2017)	12	−0.36	2.8225	15	−4.66	3.8509	4.30	[1.78; 6.82]	3.5%	20.6%
Said (2021)	15	−2.90	1.0385	16	−2.19	1.4770	−0.71	[−1.60; 0.18]	27.6%	38.2%
**Common effect model**	**43**			**47**			**−0.09**	**[−0.56; 0.37]**	**100.0%**	**−−**
**Random effects model**							**0.58**	**[−0.99; 2.16]**	**−−**	**100.0%**

Heterogeneity: $I^2 = 85\%$, $\tau^2 = 1.4774$, $p < 0.01$

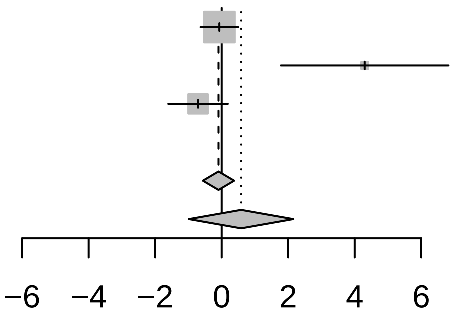

Study	AE-M Total	AE-M Mean	AE-M SD	RT-HI Total	RT-HI Mean	RT-HI SD	Mean Difference	MD	95%−CI
Fiorilli (2017)	12	−0.36	2.8225	14	−3.05	4.4892		2.69	[−0.15; 5.53]

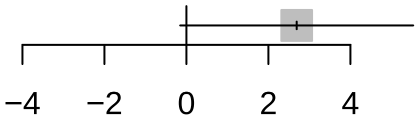

	AE-M			COM-M					
**Study**	**Total**	**Mean**	**SD**	**Total**	**Mean**	**SD**	**Mean Difference**	**MD**	**95%−CI**
Said (2021)	15	−2.90	1.0385	15	−3.01	0.7993	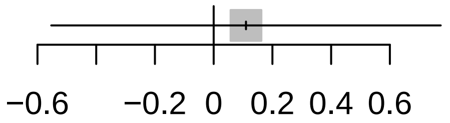	0.11	[−0.55; 0.77]

Study	AE-HI Total	AE-HI Mean	AE-HI SD	RT-HI Total	RT-HI Mean	RT-HI SD	MD	95%−CI	Weight (common)	Weight (random)
Sigal (2014)	75	−1.10	0.8989	78	−1.60	1.2166	0.50	[0.16; 0.84]	97.7%	97.7%
Bouamra (2022)	12	−2.10	2.3580	12	−2.20	3.0650	0.10	[−2.09; 2.29]	2.3%	2.3%
**Common effect model**	**87**			**90**			**0.49**	**[0.16; 0.82]**	**100.0%**	**−−**
**Random effects model**							**0.49**	**[0.16; 0.82]**	**−−**	**100.0%**

Heterogeneity: $I^2 = 0\%$, $\tau^2 = 0$, $p = 0.72$

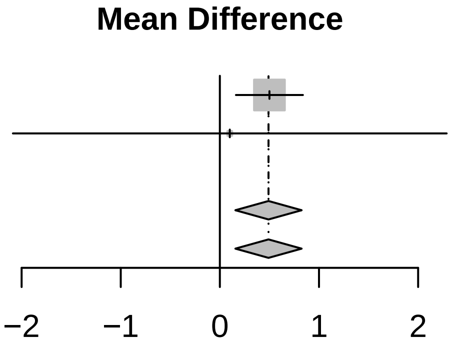

Study	AE-HI Total	AE-HI Mean	AE-HI SD	COM-HI Total	COM-HI Mean	COM-HI SD	Mean Difference	MD	95%−CI	Weight (common)	Weight (random)
Sigal (2014)	75	−1.10	0.8989	75	−1.40	1.0696		0.30	[−0.02; 0.62]	90.3%	61.8%
Monteiro (2015)	18	−3.59	1.6325	14	−2.85	1.9834		−0.74	[−2.02; 0.54]	5.5%	20.9%
Bouamra (2022)	12	−2.10	2.3580	13	−3.10	1.0909		1.00	[−0.46; 2.46]	4.2%	17.3%
**Common effect model**	**105**			**102**				**0.27**	**[−0.03; 0.57]**	**100.0%**	**−−**
**Random effects model**								**0.20**	**[−0.49; 0.90]**	**−−**	**100.0%**

Heterogeneity: $I^2 = 41\%$, $\tau^2 = 0.1791$, $p = 0.19$

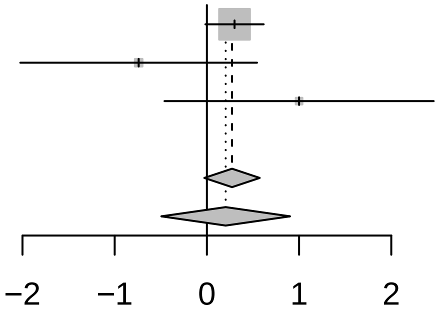

	RT-M			RT-HI					
Study	Total	Mean	SD	Total	Mean	SD	Mean Difference	MD	95%−CI
Fiorilli (2017)	15	−4.66	3.8509	14	−3.05	4.4892		−1.61	[−4.66; 1.44]

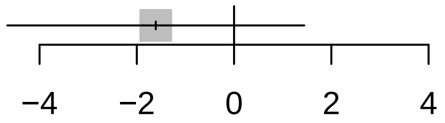

	RT-M			COM-M				
Study	Total	Mean	SD	Total	Mean	SD	MD	95%−CI
Said (2021)	16	−2.19	1.4770	15	−3.01	0.7993	0.82	[−0.01; 1.65]

Mean Difference

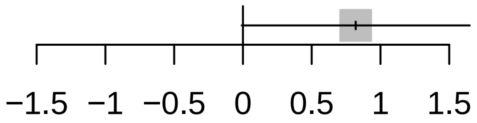

Study	RT-HI Total	RT-HI Mean	RT-HI SD	COM-HI Total	COM-HI Mean	COM-HI SD	Mean Difference	MD	95%−CI	Weight (common)	Weight (random)
Sigal (2014)	78	−1.60	1.2166	75	−1.40	1.0696		−0.20	[−0.56; 0.16]	96.2%	84.7%
Bouamra (2022)	12	−2.20	3.0650	13	−3.10	1.0909		0.90	[−0.93; 2.73]	3.8%	15.3%
**Common effect model**	**90**			**88**				**−0.16**	**[−0.51; 0.20]**	**100.0%**	**−−**
**Random effects model**								**−0.03**	**[−0.81; 0.74]**	**−−**	**100.0%**

Heterogeneity: $I^2 = 25\%$, $\tau^2 = 0.1507$, $p = 0.25$

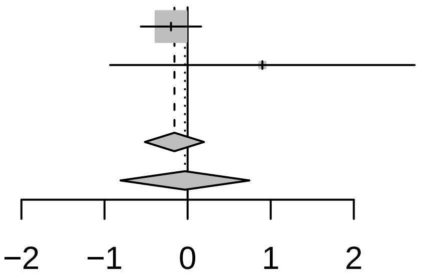

Figure 2. Pairwise Meta-Analyses for Body Mass Index

AE-M, moderate-intensity aerobic exercise; AE-HI, high-intensity aerobic exercise; RT-M, moderate-intensity resistance exercise; RT-HI, high-intensity resistance exercise; COM-M, moderate-intensity combined exercise; COM-HI, high-intensity combined exercise; CON, blank controls

Study	AE-M Total	AE-M Mean	AE-M SD	CON Total	CON Mean	CON SD	MD	95%−CI	Weight (common)	Weight (random)
KARACABEY (2009)	20	−6.20	2.0430	20	1.00	3.3045	−7.20	[−8.90; −5.50]	0.6%	5.2%
Farpour−Lambert (2009)	22	−0.10	1.0000	22	0.30	0.7000	−0.40	[−0.91; 0.11]	6.5%	9.9%
Saygin (2011)	20	−1.98	2.2243	19	0.13	1.4525	−2.11	[−3.28; −0.94]	1.2%	7.1%
Song (2011)	12	−0.50	0.8544	10	−0.10	0.7000	−0.40	[−1.05; 0.25]	4.0%	9.4%
Lee (2012)	16	−0.30	0.3000	13	0.30	0.3000	−0.60	[−0.82; −0.38]	35.1%	10.7%
Lee (2013)	16	−0.46	0.5800	12	−0.03	0.4400	−0.43	[−0.81; −0.05]	11.8%	10.4%
Tan (2015)	11	−1.23	2.3978	13	−0.20	1.7838	−1.03	[−2.75; 0.69]	0.6%	5.1%
Tan (2016)	21	−0.67	0.3100	21	0.41	0.4800	−1.08	[−1.32; −0.84]	28.3%	10.7%
Dias (2017)	32	−1.30	2.2000	34	0.10	4.5133	−1.40	[−3.10; 0.30]	0.6%	5.2%
Kim (2019)	24	−2.00	1.1832	24	0.00	0.4472	−2.00	[−2.51; −1.49]	6.6%	9.9%
Said (2021)	15	−2.40	1.1023	15	−1.60	0.8414	−0.80	[−1.50; −0.10]	3.4%	9.2%
Cao (2022)	11	−3.10	0.9220	13	1.20	1.9000	−4.30	[−5.47; −3.13]	1.2%	7.2%
**Common effect model**	**220**			**216**			**−0.90**	**[−1.03; −0.77]**	**100.0%**	**--**
**Random effects model**							**−1.53**	**[−2.06; −1.00]**	**--**	**100.0%**

Heterogeneity: $I^2 = 91\%$, $\tau^2 = 0.6737$, $p < 0.01$

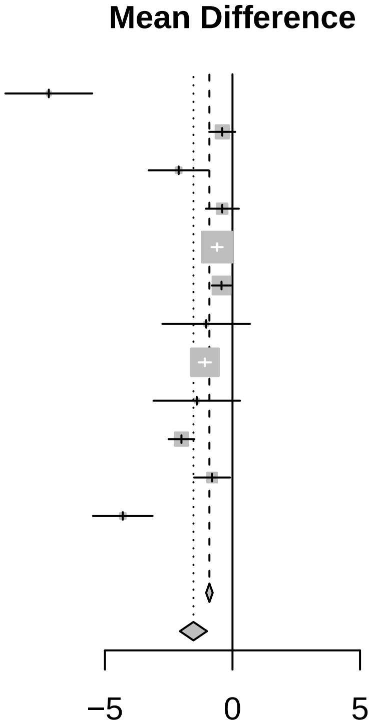

Study	AE-HI Total	AE-HI Mean	AE-HI SD	CON Total	CON Mean	CON SD	MD	95%−CI	Weight (common)	Weight (random)
Lee (2010)	16	−0.28	1.1077	18	−0.61	3.1949	0.33	[−1.24; 1.90]	1.0%	8.9%
Regaieg(2012)	14	−0.60	3.1512	14	0.50	3.8510	−1.10	[−3.71; 1.51]	0.4%	5.1%
Youssef (2015)	14	−0.03	0.2000	9	0.40	0.2000	−0.43	[−0.60; −0.26]	86.7%	15.5%
Monteiro (2015)	18	−1.44	1.3289	16	1.09	1.5440	−2.53	[−3.50; −1.56]	2.6%	12.1%
Vasconcellos (2015)	10	−0.70	2.3169	10	1.30	2.5483	−2.00	[−4.13; 0.13]	0.5%	6.5%
Khammassi (2018)	10	−1.30	1.1446	10	0.20	0.9839	−1.50	[−2.44; −0.56]	2.8%	12.3%
Dias (2017)	33	−0.70	1.6661	34	0.10	4.5133	−0.80	[−2.42; 0.82]	0.9%	8.7%
Cvetkovic (2018)	10	−0.78	1.8566	14	0.87	2.0885	−1.65	[−3.24; −0.06]	1.0%	8.8%
Martín−García (2017)	19	−0.50	1.6100	28	−0.20	1.4121	−0.30	[−1.19; 0.59]	3.0%	12.5%
Cao (2022)	12	−2.20	1.7692	13	1.20	1.9000	−3.40	[−4.84; −1.96]	1.2%	9.6%
**Common effect model**	**156**			**166**			**−0.56**	**[−0.72; −0.41]**	**100.0%**	**−−**
**Random effects model**							**−1.29**	**[−2.01; −0.58]**	**−−**	**100.0%**

Heterogeneity: $I^2 = 79\%$, $\tau^2 = 0.8552$, $p < 0.01$

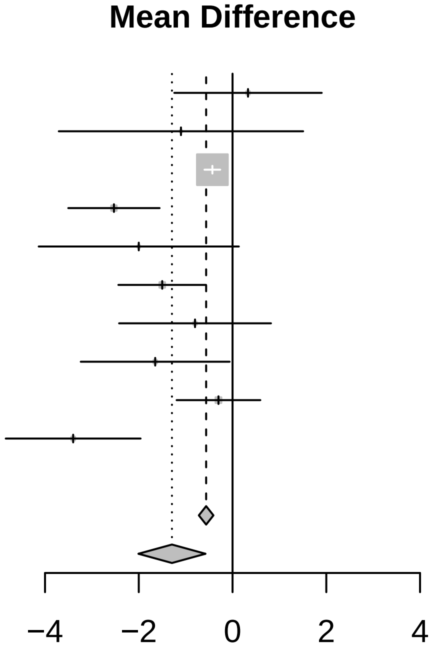

Study	RT-M Total	RT-M Mean	RT-M SD	CON Total	CON Mean	CON SD	MD	95%−CI	Weight (common)	Weight (random)
Lee (2012)	16	−0.60	0.3000	13	0.30	0.3000	−0.90	[−1.12; −0.68]	65.1%	30.3%
Lee (2013)	16	−0.28	0.5400	12	−0.03	0.4400	−0.25	[−0.61; 0.11]	23.8%	28.2%
Alberga (2013)	12	0.28	0.7200	7	−0.31	0.8500	0.59	[−0.16; 1.34]	5.6%	20.7%
Said (2021)	16	−2.00	1.2534	15	−1.60	0.8414	−0.40	[−1.15; 0.35]	5.6%	20.8%
**Common effect model**	**60**			**47**			**−0.63**	**[−0.81; −0.46]**	**100.0%**	**--**
**Random effects model**							**−0.30**	**[−0.89; 0.28]**	**--**	**100.0%**

Heterogeneity: $I^2 = 85\%$, $\tau^2 = 0.2785$, $p < 0.01$

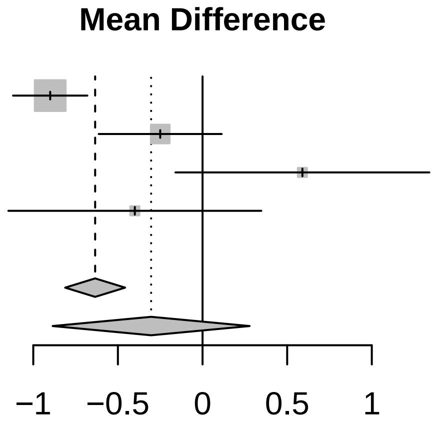

Study	RT-HI Total	RT-HI Mean	RT-HI SD	CON Total	CON Mean	CON SD	Mean Difference	MD	95%−CI
SHAIBI (2006)	11	0.30	0.4000	11	0.40	0.3000	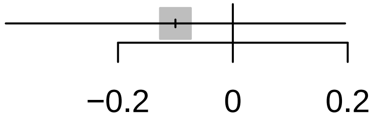	−0.10	[−0.40; 0.20]

Study	COM-M Total	COM-M Mean	COM-M SD	CON Total	CON Mean	CON SD	MD	95%−CI	Weight (common)	Weight (random)
Wong (2008)	12	−1.20	1.2907	12	−0.10	1.9677	−1.10	[−2.43; 0.23]	5.7%	14.6%
Lee (2010)	20	0.53	2.8019	18	−0.61	3.1949	1.14	[−0.78; 3.06]	2.7%	11.0%
Jeon (2013)	8	−2.27	1.1839	7	0.13	0.7515	−2.40	[−3.39; −1.41]	10.2%	16.9%
Wong (2017)	15	−2.80	0.9664	15	0.00	0.6132	−2.80	[−3.38; −2.22]	30.0%	19.4%
Duft (2020)	18	−0.41	1.0854	19	0.29	1.2940	−0.70	[−1.47; 0.07]	17.1%	18.4%
Said (2021)	15	−2.76	0.6622	15	−1.60	0.8414	−1.16	[−1.70; −0.62]	34.3%	19.6%
**Common effect model**	**88**			**86**			**−1.63**	**[−1.95; −1.32]**	**100.0%**	**--**
**Random effects model**							**−1.34**	**[−2.27; −0.42]**	**--**	**100.0%**

Heterogeneity: $I^2 = 86\%$, $\tau^2 = 1.0538$, $p < 0.01$

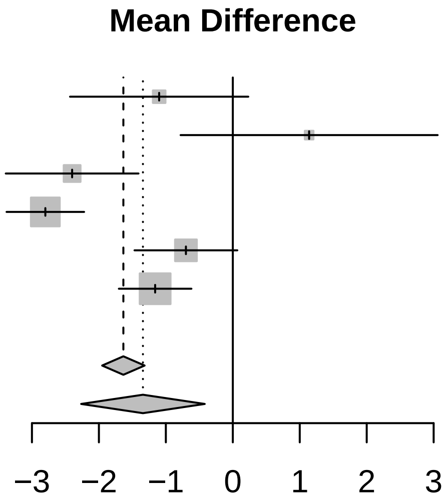

Study	COM-HI Total	COM-HI Mean	COM-HI SD	CON Total	CON Mean	CON SD	Mean Difference	MD	95%−CI
Monteiro (2015)	14	−0.81	2.0616	16	1.09	1.5440	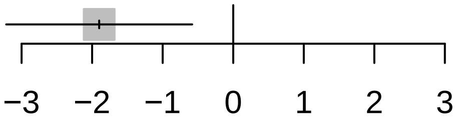	−1.90	[−3.22; −0.58]

Study	AE-M Total	AE-M Mean	AE-M SD	AE-HI Total	AE-HI Mean	AE-HI SD	MD	95%−CI	Weight (common)	Weight (random)
Dias (2017)	32	−1.30	2.2000	33	−0.70	1.6661	−0.60	[−1.55; 0.35]	59.0%	59.0%
Cao (2022)	11	−3.10	0.9220	12	−2.20	1.7692	−0.90	[−2.04; 0.24]	41.0%	41.0%
**Common effect model**	**43**			**45**			**−0.72**	**[−1.45; 0.01]**	**100.0%**	**−−**
**Random effects model**							**−0.72**	**[−1.45; 0.01]**	**−−**	**100.0%**

Heterogeneity: $I^2 = 0\%$, $\tau^2 = 0$, $p = 0.69$

Mean Difference

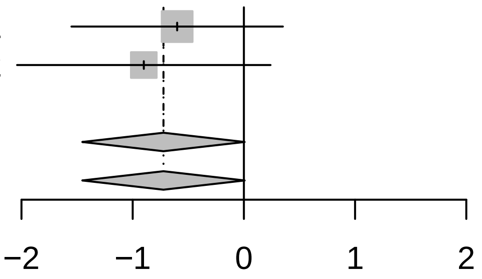

Study	AE-M Total	AE-M Mean	AE-M SD	RT-M Total	RT-M Mean	RT-M SD	MD	95%−CI	Weight (common)	Weight (random)
Lee (2012)	16	−0.30	0.3000	16	−0.60	0.3000	0.30	[0.09; 0.51]	32.4%	23.5%
Lee (2013)	16	−0.46	0.5800	16	−0.28	0.5400	−0.18	[−0.57; 0.21]	9.3%	18.4%
Fiorilli (2017)	12	−0.48	1.7110	15	−0.85	2.5689	0.37	[−1.25; 1.99]	0.5%	3.1%
Deldin (2019) Boy	14	−0.70	0.3000	14	−0.30	0.3000	−0.40	[−0.62; −0.18]	28.4%	23.1%
Deldin (2019) Girl	13	−0.50	0.3000	14	−0.30	0.3000	−0.20	[−0.43; 0.03]	27.3%	23.0%
Said (2021)	15	−2.40	1.1023	16	−2.00	1.2534	−0.40	[−1.23; 0.43]	2.0%	8.8%
**Common effect model**	**86**			**91**			**−0.09**	**[−0.21; 0.02]**	**100.0%**	**−−**
**Random effects model**							**−0.13**	**[−0.43; 0.18]**	**−−**	**100.0%**

Heterogeneity: $I^2 = 78\%$, $\tau^2 = 0.0896$, $p < 0.01$

Mean Difference

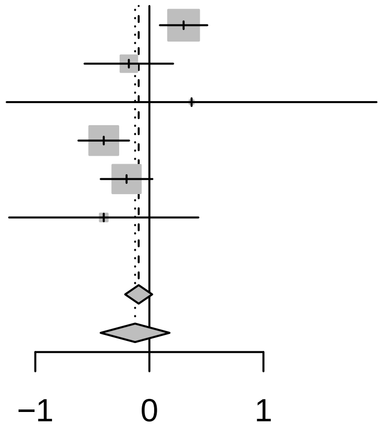

Study	AE-M Total	AE-M Mean	AE-M SD	RT-HI Total	RT-HI Mean	RT-HI SD	Mean Difference	MD	95%−CI
Fiorilli (2017)	12	−0.48	1.7110	14	0.36	2.3483	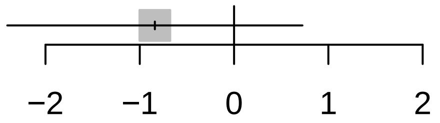	−0.84	[−2.41; 0.73]

Study	AE-M Total	AE-M Mean	AE-M SD	COM-M Total	COM-M Mean	COM-M SD	Mean Difference	MD	95%−CI
Said (2021)	15	−2.40	1.1023	15	−2.76	0.6622	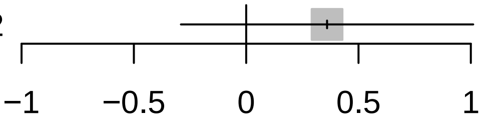	0.36	[−0.29; 1.01]

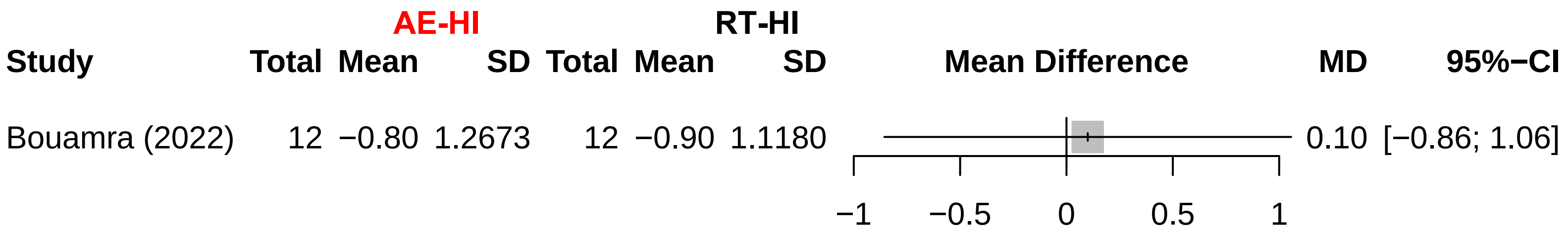
AE-HI
RT-HI
Study
Total
Mean
SD
Total
Mean
SD
Mean Difference
MD
95%−CI
Bouamra (2022)
12
−0.80
1.2673
12
−0.90
1.1180
0.10
[−0.86; 1.06]
−1
−0.5
0
0.5
1

Study	AE-HI Total	AE-HI Mean	AE-HI SD	COM-M Total	COM-M Mean	COM-M SD	Mean Difference	MD	95%−CI
Lee (2010)	16	−0.28	1.1077	20	0.53	2.8019		−0.81	[−2.15; 0.53]

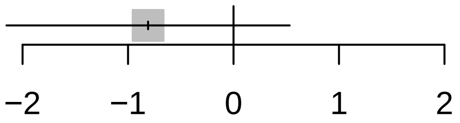

Study	AE-HI Total	AE-HI Mean	AE-HI SD	COM-HI Total	COM-HI Mean	COM-HI SD	MD	95%−CI	Weight (common)	Weight (random)
Monteiro (2015)	18	−1.44	1.3289	14	−0.81	2.0616	−0.63	[−1.87; 0.61]	44.5%	48.9%
Bouamra (2022)	12	−0.80	1.2673	13	−2.10	1.5652	1.30	[0.19; 2.41]	55.5%	51.1%
**Common effect model**	**30**			**27**			**0.44**	**[−0.39; 1.27]**	**100.0%**	**−−**
**Random effects model**							**0.36**	**[−1.54; 2.25]**	**−−**	**100.0%**

Heterogeneity: $I^2 = 81\%$, $\tau^2 = 1.5005$, $p = 0.02$

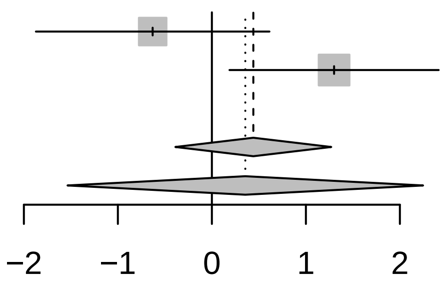

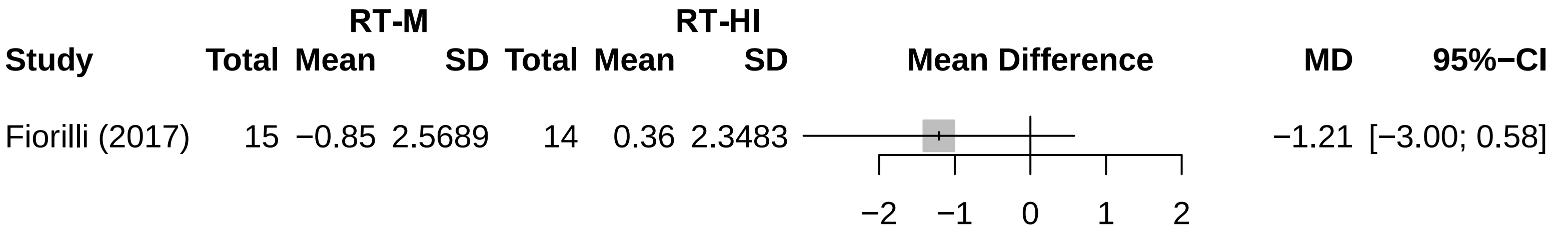
RT-M
RT-HI
Study
Total
Mean
SD
Total
Mean
SD
Mean Difference
MD
95%−CI
Fiorilli (2017)
15
−0.85
2.5689
14
0.36
2.3483
−1.21
[−3.00; 0.58]
−2
−1
0
1
2

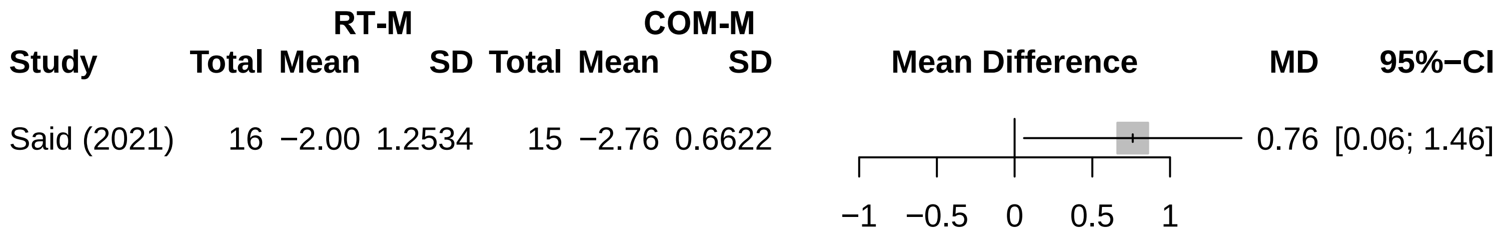
RT-M
COM-M
Study
Total
Mean
SD
Total
Mean
SD
Mean Difference
MD
95%−CI
Said (2021)
16
−2.00
1.2534
15
−2.76
0.6622
0.76
[0.06; 1.46]
−1
−0.5
0
0.5
1

Study	RT-HI Total	RT-HI Mean	RT-HI SD	COM-HI Total	COM-HI Mean	COM-HI SD	Mean Difference	MD	95%−CI
Bouamra (2022)	12	−0.90	1.1180	13	−2.10	1.5652	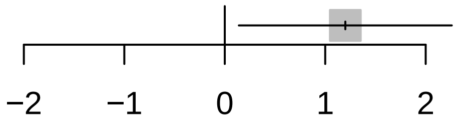	1.20	[0.14; 2.26]

Figure 3. Pairwise Meta-Analyses for Fat Mass

AE-M, moderate-intensity aerobic exercise; AE-HI, high-intensity aerobic exercise; RT-HI, high-intensity resistance exercise; COM-M, moderate-intensity combined exercise; COM-HI, high-intensity combined exercise; CON, blank controls

	AE-M			CON					Weight	Weight
**Study**	**Total**	**Mean**	**SD**	**Total**	**Mean**	**SD**	**MD**	**95%−CI**	**(common)**	**(random)**
Song 2011	12	−1.40	1.5100	10	0.10	1.2530	−1.50	[−2.65; −0.35]	1.0%	22.3%
Tan 2015	11	−1.20	4.8785	13	0.40	3.1847	−1.60	[−4.96; 1.76]	0.1%	14.1%
Tan 2016	21	−0.50	0.1800	21	0.81	0.2100	−1.31	[−1.43; −1.19]	97.9%	24.1%
Dias 2017	32	−0.70	4.7445	34	−0.10	4.7764	−0.60	[−2.90; 1.70]	0.3%	18.1%
Cao 2022	11	−3.70	2.1373	13	2.80	1.1832	−6.50	[−7.92; −5.08]	0.7%	21.4%
**Common effect model**	**87**			**91**			**−1.35**	**[−1.46; −1.23]**	**100.0%**	**--**
**Random effects model**							**−2.38**	**[−4.33; −0.42]**	**--**	**100.0%**

Heterogeneity: $I^2 = 92\%$, $\tau^2 = 4.1233$, $p < 0.01$

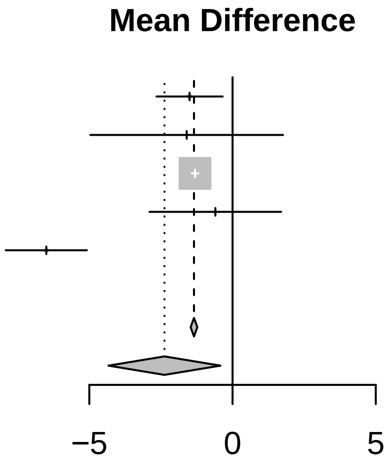

Study	AE-HI Total	AE-HI Mean	AE-HI SD	CON Total	CON Mean	CON SD	MD	95%−CI	Weight (common)	Weight (random)
Sigal 2014	75	−1.20	0.6116	76	0.40	1.3528	−1.60	[−1.93; −1.27]	60.9%	19.6%
Monteiro 2015	18	−3.12	3.0730	16	1.22	3.2447	−4.34	[−6.47; −2.21]	1.5%	12.3%
Dias 2017	33	−0.90	4.9029	34	−0.10	4.7764	−0.80	[−3.12; 1.52]	1.3%	11.5%
Cvetkovic 2018	10	−1.94	3.7073	14	0.87	4.7423	−2.81	[−6.19; 0.57]	0.6%	7.8%
Martín−García 2017	19	−0.90	3.7616	28	0.20	4.4317	−1.10	[−3.46; 1.26]	1.2%	11.4%
Cao 2022	12	−2.40	0.9990	13	2.80	1.1832	−5.20	[−6.06; −4.34]	9.3%	18.1%
Salus 2022	14	−1.20	0.7000	14	0.50	0.7000	−1.70	[−2.22; −1.18]	25.3%	19.2%
**Common effect model**	**181**			**195**			**−1.99**	**[−2.25; −1.73]**	**100.0%**	**--**
**Random effects model**							**−2.55**	**[−3.77; −1.34]**	**--**	**100.0%**

Heterogeneity: $I^2 = 91\%$, $\tau^2 = 1.9360$, $p < 0.01$

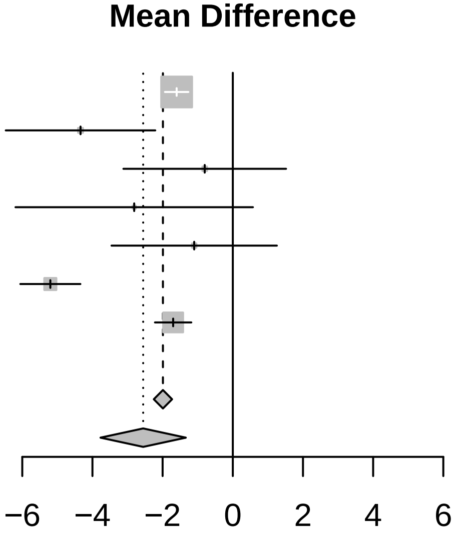

Study	RT-HI Total	RT-HI Mean	RT-HI SD	CON Total	CON Mean	CON SD	Mean Difference	MD	95%−CI	Weight (common)	Weight (random)
SHAIBI 2006	11	−1.30	0.9000	11	−0.20	0.7000		−1.10	[−1.77; −0.43]	19.6%	37.6%
Sigal 2014	78	−1.30	0.6116	76	0.40	1.3528		−1.70	[−2.03; −1.37]	80.4%	62.4%
**Common effect model**	**89**			**87**				**−1.58**	**[−1.88; −1.28]**	**100.0%**	**−−**
**Random effects model**								**−1.47**	**[−2.04; −0.90]**	**−−**	**100.0%**

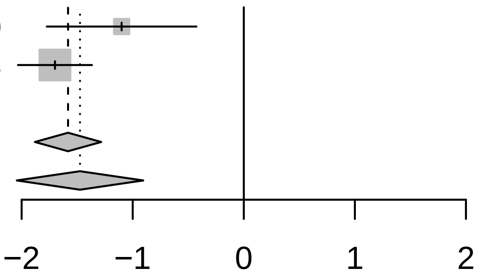

Heterogeneity: $I^2 = 59\%$, $\tau^2 = 0.1065$, $p = 0.12$

Study	COM-M Total	COM-M Mean	COM-M SD	CON Total	CON Mean	CON SD	MD	95%−CI	Weight (common)	Weight (random)
Wong 2008	12	−0.10	2.5346	12	−0.80	3.3988	0.70	[−1.70; 3.10]	19.0%	31.1%
Jeon 2013	8	−3.91	1.5780	7	0.12	1.1748	−4.03	[−5.43; −2.63]	56.1%	36.2%
Duft 2020	18	−2.85	3.3810	19	1.85	3.1189	−4.70	[−6.80; −2.60]	24.9%	32.7%
**Common effect model**	**38**			**38**			**−3.30**	**[−4.34; −2.25]**	**100.0%**	**−−**
**Random effects model**							**−2.78**	**[−5.68; 0.12]**	**−−**	**100.0%**

Heterogeneity: $I^2 = 85\%$, $\tau^2 = 5.5550$, $p < 0.01$

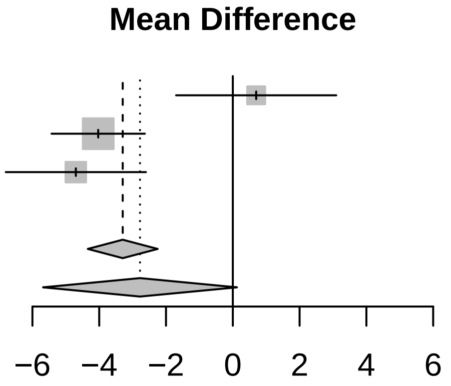

Study	COM-HI Total	COM-HI Mean	COM-HI SD	CON Total	CON Mean	CON SD	MD	95%−CI	Weight (common)	Weight (random)
Sigal 2014	75	−1.60	1.3528	76	0.40	1.3528	−2.00	[−2.43; −1.57]	97.6%	78.4%
Monteiro 2015	14	−2.61	4.2545	16	1.22	3.2447	−3.83	[−6.57; −1.09]	2.4%	21.6%
**Common effect model**	**89**			**92**			**−2.04**	**[−2.47; −1.62]**	**100.0%**	**−−**
**Random effects model**							**−2.40**	**[−3.87; −0.92]**	**−−**	**100.0%**

Heterogeneity: $I^2 = 40\%$, $\tau^2 = 0.6748$, $p = 0.20$

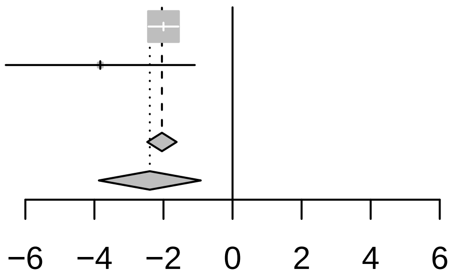

Study	AE-M Total	AE-M Mean	AE-M SD	AE-HI Total	AE-HI Mean	AE-HI SD	MD	95%−CI	Weight (common)	Weight (random)
Dias 2017	32	−0.70	4.7445	33	−0.90	4.9029	0.20	[−2.15; 2.55]	25.8%	29.3%
Cao 2022	11	−3.70	2.1373	12	−2.40	0.9990	−1.30	[−2.68; 0.08]	74.2%	70.7%
**Common effect model**	**43**			**45**			**−0.91**	**[−2.10; 0.28]**	**100.0%**	**−−**
**Random effects model**							**−0.86**	**[−2.20; 0.48]**	**−−**	**100.0%**

Heterogeneity: $I^2 = 14\%$, $\tau^2 = 0.1598$, $p = 0.28$

Mean Difference

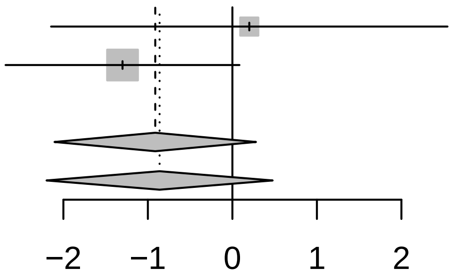

	AE-HI			RT-HI						
**Study**	**Total**	**Mean**	**SD**	**Total**	**Mean**	**SD**	**MD**	**95%−CI**	**Weight (common)**	**Weight (random)**
Sigal 2014	75	−1.20	0.6116	78	−1.30	0.6116	0.10	[−0.09; 0.29]	99.1%	99.1%
Bouamra 2022	12	−2.40	2.1817	12	−1.70	2.7557	−0.70	[−2.69; 1.29]	0.9%	0.9%
**Common effect model**	**87**			**90**			**0.09**	**[−0.10; 0.29]**	**100.0%**	**−−**
**Random effects model**							**0.09**	**[−0.10; 0.29]**	**−−**	**100.0%**

Heterogeneity: $I^2 = 0\%$, $\tau^2 = 0$, $p = 0.43$

Mean Difference

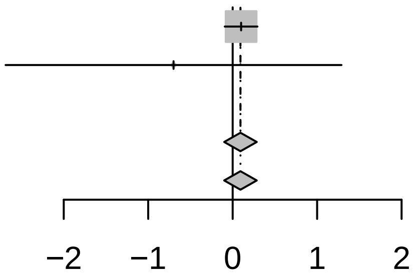

Study	AE-HI Total	AE-HI Mean	AE-HI SD	COM-HI Total	COM-HI Mean	COM-HI SD	Mean Difference	MD	95%−CI	Weight (common)	Weight (random)
Sigal 2014	75	−1.20	0.6116	75	−1.60	1.3528		0.40	[0.06; 0.74]	95.9%	80.2%
Monteiro 2015	18	−3.12	3.0730	14	−2.61	4.2545		−0.51	[−3.15; 2.13]	1.6%	7.8%
Bouamra 2022	12	−2.40	2.1817	13	−4.30	3.0529		1.90	[−0.17; 3.97]	2.5%	12.1%
**Common effect model**	**105**			**102**				**0.42**	**[0.09; 0.75]**	**100.0%**	**−−**
**Random effects model**								**0.51**	**[−0.26; 1.28]**	**−−**	**100.0%**

Heterogeneity: $I^2 = 19\%$, $\tau^2 = 0.1625$, $p = 0.29$

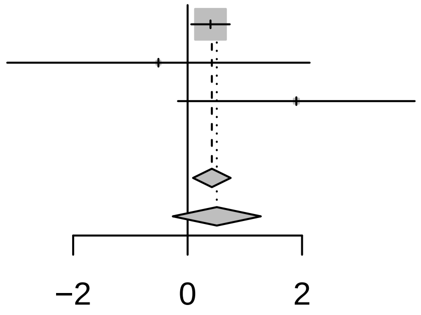

Study	RT-HI Total	RT-HI Mean	RT-HI SD	COM-HI Total	COM-HI Mean	COM-HI SD	MD	95%−CI	Weight (common)	Weight (random)
Sigal 2014	78	−1.30	0.6116	75	−1.60	1.3528	0.30	[−0.03; 0.63]	97.9%	62.5%
Bouamra 2022	12	−1.70	2.7557	13	−4.30	3.0529	2.60	[0.32; 4.88]	2.1%	37.5%
**Common effect model**	**90**			**88**			**0.35**	**[0.02; 0.68]**	**100.0%**	**−−**
**Random effects model**							**1.16**	**[−1.02; 3.35]**	**−−**	**100.0%**

Heterogeneity: $I^2 = 74\%$, $\tau^2 = 1.9555$, $p = 0.05$

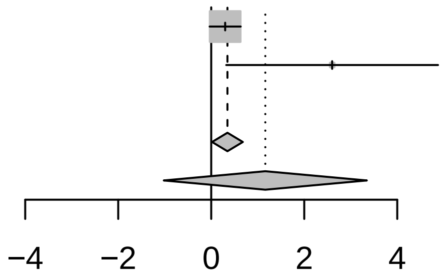

Figure 4. Pairwise Meta-Analyses for Fat-Free Mass

AE-M, moderate-intensity aerobic exercise; AE-HI, high-intensity aerobic exercise; RT-M, moderate-intensity resistance exercise; RT-HI, high-intensity resistance exercise; COM-M, moderate-intensity combined exercise; COM-HI, high-intensity combined exercise; CON, blank controls

	AE-M			CON					Weight	Weight
**Study**	**Total**	**Mean**	**SD**	**Total**	**Mean**	**SD**	**MD**	**95%−CI**	**(common)**	**(random)**
Farpour−Lambert (2009)	22	1.20	1.3000	22	−0.10	2.8000	1.30	[0.01; 2.59]	2.0%	4.4%
Song (2011)	12	0.70	1.7321	10	0.80	2.3000	−0.10	[−1.83; 1.63]	1.1%	2.5%
Lee (2012)	16	1.23	0.3400	13	1.38	0.3900	−0.15	[−0.42; 0.12]	46.1%	44.2%
Tan (2015)	11	0.20	2.3702	13	0.00	2.2445	0.20	[−1.66; 2.06]	1.0%	2.2%
Tan (2016)	21	0.66	0.3500	21	0.74	0.5000	−0.08	[−0.34; 0.18]	49.2%	45.4%
Dias (2017)	32	0.90	5.1317	34	−0.70	4.6433	1.60	[−0.77; 3.97]	0.6%	1.4%
**Common effect model**	**114**			**113**			**−0.07**	**[−0.26; 0.11]**	**100.0%**	**−−**
**Random effects model**							**−0.02**	**[−0.30; 0.26]**	**−−**	**100.0%**

Heterogeneity: $I^2 = 25\%$, $\tau^2 = 0.0268$, $p = 0.25$

Mean Difference

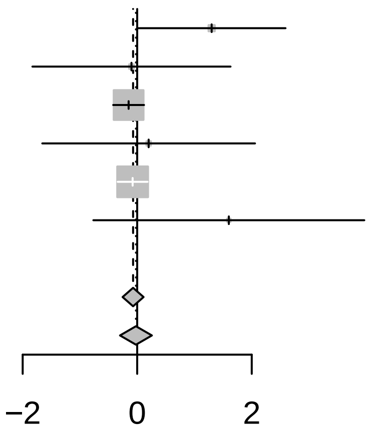

Study	AE-HI Total	AE-HI Mean	AE-HI SD	CON Total	CON Mean	CON SD	MD	95%−CI	Weight (common)	Weight (random)
Sigal (2014)	75	0.70	0.7000	76	1.10	0.7000	−0.40	[−0.62; −0.18]	61.2%	26.2%
Youssef (2015)	14	1.50	0.4000	9	−0.25	0.6000	1.75	[1.31; 2.19]	15.4%	25.3%
Dias (2017)	33	1.60	3.9666	34	−0.70	4.6433	2.30	[0.23; 4.37]	0.7%	12.9%
Martín−García (2017)	19	0.70	4.7718	28	0.70	3.9590	0.00	[−2.60; 2.60]	0.5%	9.9%
Salus (2022)	14	2.30	0.5000	14	2.30	0.5000	0.00	[−0.37; 0.37]	22.2%	25.7%
**Common effect model**	**155**			**161**			**0.04**	**[−0.13; 0.22]**	**100.0%**	**−−**
**Random effects model**							**0.63**	**[−0.40; 1.66]**	**−−**	**100.0%**

Heterogeneity: $I^2 = 95\%$, $\tau^2 = 1.0405$, $p < 0.01$

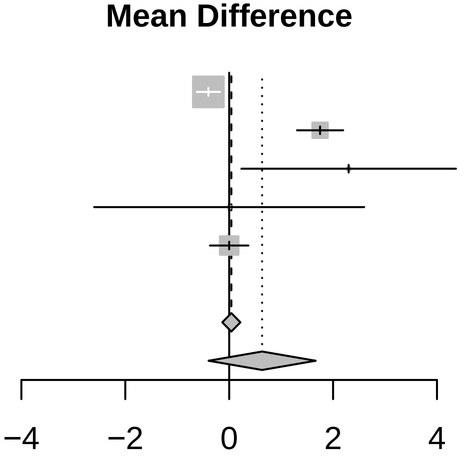

Study	RT-M Total	RT-M Mean	RT-M SD	CON Total	CON Mean	CON SD	Mean Difference	MD	95%−CI	Weight (common)	Weight (random)
Lee (2012)	16	2.11	0.3300	13	1.38	0.3900		0.73	[0.46; 1.00]	81.1%	81.1%
Alberga (2013)	12	1.40	0.6300	7	0.81	0.5700		0.59	[0.04; 1.14]	18.9%	18.9%
**Common effect model**	**28**			**20**				**0.70**	**[0.46; 0.94]**	**100.0%**	**−−**
**Random effects model**								**0.70**	**[0.46; 0.94]**	**−−**	**100.0%**

Heterogeneity: $I^2 = 0\%$, $\tau^2 = 0$, $p = 0.65$

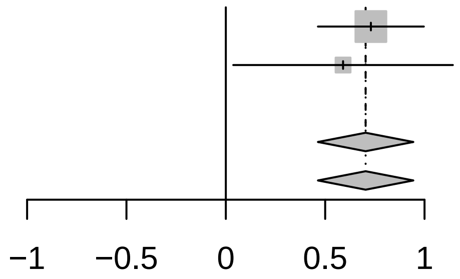

Study	RT-HI Total	RT-HI Mean	RT-HI SD	CON Total	CON Mean	CON SD	MD	95%−CI	Weight (common)	Weight (random)
SHAIBI (2006)	11	3.70	0.9000	11	2.00	0.6000	1.70	[1.06; 2.34]	10.7%	47.6%
Sigal (2014)	78	1.40	0.7000	76	1.10	0.7000	0.30	[0.08; 0.52]	89.3%	52.4%
**Common effect model**	**89**			**87**			**0.45**	**[0.24; 0.66]**	**100.0%**	**−−**
**Random effects model**							**0.97**	**[−0.40; 2.34]**	**−−**	**100.0%**

Heterogeneity: $I^2 = 94\%$, $\tau^2 = 0.9205$, $p < 0.01$

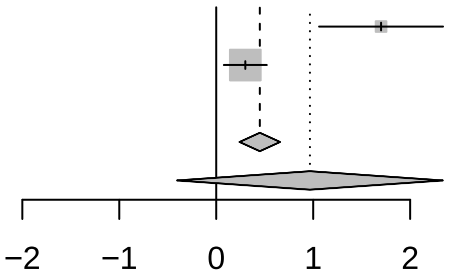

Study	COM-M Total	Mean	SD	CON Total	Mean	SD	Mean Difference	MD	95%−CI
Wong (2008)	12	1.60	3.2345	12	1.60	5.1736		0.00	[−3.45; 3.45]

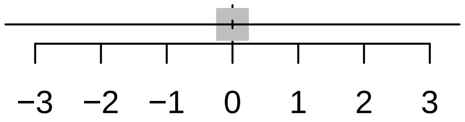

	COM-HI			CON					
Study	Total	Mean	SD	Total	Mean	SD	Mean Difference	MD	95%−CI
Sigal (2014)	75	0.80	0.7000	76	1.10	0.7000		−0.30	[−0.52; −0.08]

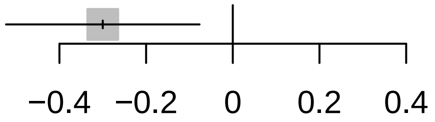

Study	AE-M Total	AE-M Mean	AE-M SD	AE-HI Total	AE-HI Mean	AE-HI SD	MD	95%−CI
Dias (2017)	32	0.90	5.1317	33	1.60	3.9666	−0.70	[−2.93; 1.53]

Mean Difference

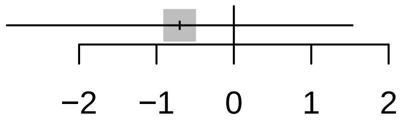

Study	AE-M Total	AE-M Mean	AE-M SD	RT-M Total	RT-M Mean	RT-M SD	Mean Difference	MD	95%−CI	Weight (common)	Weight (random)
Lee (2012)	16	1.23	0.3400	16	2.11	0.3300		−0.88	[−1.11; −0.65]	99.8%	99.8%
Fiorilli (2017)	12	2.88	7.2124	15	3.12	4.4692		−0.24	[−4.91; 4.43]	0.2%	0.2%
**Common effect model**	**28**			**31**				**−0.88**	**[−1.11; −0.65]**	**100.0%**	**−−**
**Random effects model**								**−0.88**	**[−1.11; −0.65]**	**−−**	**100.0%**

Heterogeneity: $I^2 = 0\%$, $\tau^2 = 0$, $p = 0.79$

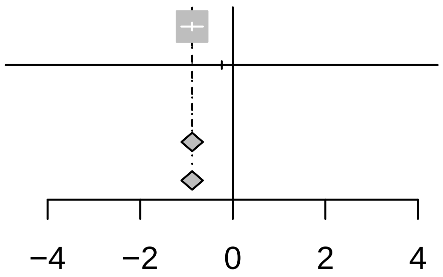

Study	AE-M Total	AE-M Mean	AE-M SD	RT-H Total	RT-H Mean	RT-H SD	Mean Difference	MD	95%−CI
Fiorilli (2017)	12	2.88	7.2124	14	4.52	4.1497		−1.64	[−6.26; 2.98]

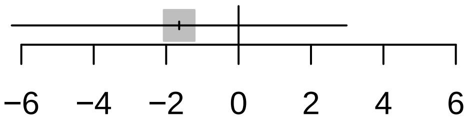

Study	AE-HI Total	AE-HI Mean	AE-HI SD	COM-HI Total	COM-HI Mean	COM-HI SD	Mean Difference	MD	95%−CI	Weight (common)	Weight (random)
Sigal (2014)	75	0.70	0.7000	75	0.80	0.7000		−0.10	[−0.32; 0.12]	99.3%	99.3%
Bouamra (2022)	12	1.30	3.5674	13	1.50	3.2109		−0.20	[−2.87; 2.47]	0.7%	0.7%
**Common effect model**	**87**			**88**				**−0.10**	**[−0.32; 0.12]**	**100.0%**	**--**
**Random effects model**								**−0.10**	**[−0.32; 0.12]**	**--**	**100.0%**

Heterogeneity: $I^2 = 0\%$, $\tau^2 = 0$, $p = 0.94$

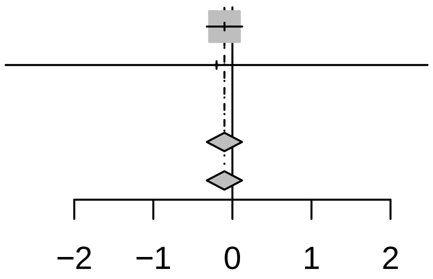

Study	AE-HI Total	AE-HI Mean	AE-HI SD	RT-HI Total	RT-HI Mean	RT-HI SD	MD	95%−CI	Weight (common)	Weight (random)
Sigal (2014)	75	0.70	0.7000	78	1.40	0.7000	−0.70	[−0.92; −0.48]	99.3%	99.3%
Bouamra (2022)	12	1.30	3.5674	12	0.70	2.9580	0.60	[−2.02; 3.22]	0.7%	0.7%
**Common effect model**	**87**			**90**			**−0.69**	**[−0.91; −0.47]**	**100.0%**	**−−**
**Random effects model**							**−0.69**	**[−0.91; −0.47]**	**−−**	**100.0%**

Heterogeneity: $I^2 = 0\%$, $\tau^2 = 0$, $p = 0.33$

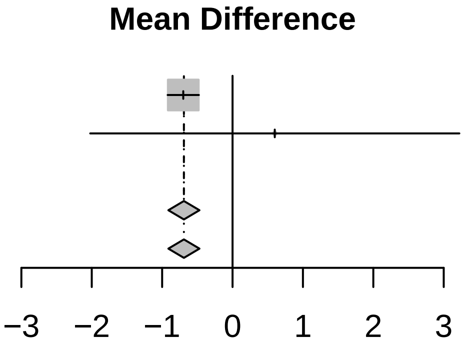

Study	RH-HI Total	RH-HI Mean	RH-HI SD	COM-HI Total	COM-HI Mean	COM-HI SD	MD	95%−CI	Weight (common)	Weight (random)
Sigal (2014)	78	1.40	0.7000	75	0.80	0.7000	0.60	[0.38; 0.82]	99.2%	88.5%
Bouamra (2022)	12	0.70	2.9580	13	1.50	3.2109	−0.80	[−3.22; 1.62]	0.8%	11.5%
**Common effect model**	**90**			**88**			**0.59**	**[0.37; 0.81]**	**100.0%**	**−−**
**Random effects model**							**0.44**	**[−0.44; 1.31]**	**−−**	**100.0%**

Heterogeneity: $I^2 = 22\%$, $\tau^2 = 0.2125$, $p = 0.26$

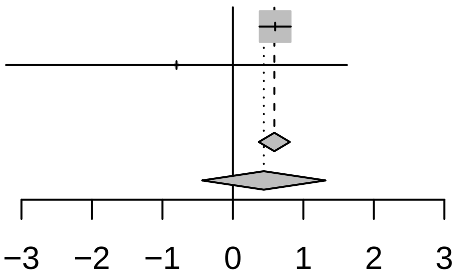

	RT-M			RT-HI					
Study	Total	Mean	SD	Total	Mean	SD	Mean Difference	MD	95%−CI
Fiorilli (2017)	15	3.12	4.4692	14	4.52	4.1497		−1.40	[−4.54; 1.74]

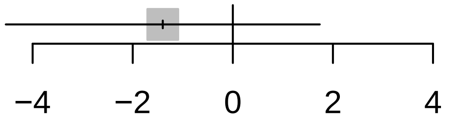

Figure 5. Pairwise Meta-Analyses for Weight

AE-M, moderate-intensity aerobic exercise; AE-HI, high-intensity aerobic exercise; RT-M, moderate-intensity resistance exercise; RT-HI, high-intensity resistance exercise; COM-M, moderate-intensity combined exercise; COM-HI, high-intensity combined exercise; CON, blank controls

Study	AE-M Total	AE-M Mean	AE-M SD	CON Total	CON Mean	CON SD	MD	95%−CI	Weight (common)	Weight (random)
KARACABEY (2009)	20	−5.20	3.5358	20	0.50	4.2192	−5.70	[−8.11; −3.29]	1.0%	4.2%
Farpour−Lambert (2009)	22	1.00	2.4000	22	1.60	1.5000	−0.60	[−1.78; 0.58]	4.3%	10.7%
Saygin (2011)	20	−2.60	8.1480	19	1.06	4.2861	−3.66	[−7.72; 0.40]	0.4%	1.7%
Song (2011)	12	0.30	2.9513	10	1.20	3.6592	−0.90	[−3.72; 1.92]	0.8%	3.3%
Lee (2012)	16	−0.04	0.8000	13	2.60	1.0000	−2.64	[−3.31; −1.97]	13.5%	15.9%
Lee (2013)	16	−1.31	1.4300	12	0.13	1.1000	−1.44	[−2.38; −0.50]	6.9%	13.0%
Tan (2015)	11	−1.00	7.0687	13	0.40	4.6998	−1.40	[−6.30; 3.50]	0.3%	1.2%
Racil (2016)	16	−1.70	2.8373	14	0.30	2.3169	−2.00	[−3.85; −0.15]	1.8%	6.3%
Tan (2016)	21	0.15	0.3900	21	1.64	0.6600	−1.49	[−1.82; −1.16]	56.4%	19.2%
Dias (2017)	32	−3.80	9.4864	34	−0.50	8.9594	−3.30	[−7.76; 1.16]	0.3%	1.5%
Kim (2019)	24	−3.30	4.5754	24	−0.60	4.3614	−2.70	[−5.23; −0.17]	0.9%	3.9%
Said (2021)	15	−7.50	4.9968	15	−4.76	2.0800	−2.74	[−5.48; −0.00]	0.8%	3.4%
Cao (2022)	11	−1.20	0.8185	13	1.00	0.9165	−2.20	[−2.89; −1.51]	12.6%	15.6%
**Common effect model**	**236**			**230**			**−1.78**	**[−2.02; −1.53]**	**100.0%**	**--**
**Random effects model**							**−2.03**	**[−2.58; −1.47]**	**--**	**100.0%**

Heterogeneity: $I^2 = 57\%$, $\tau^2 = 0.3922$, $p < 0.01$

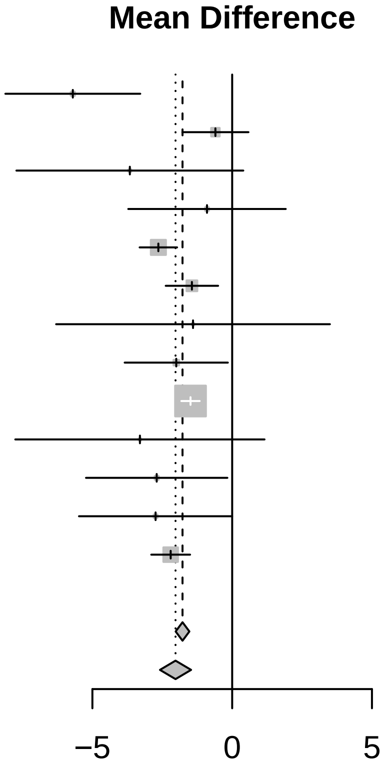

Study	AE-HI Total	AE-HI Mean	AE-HI SD	CON Total	CON Mean	CON SD	MD	95%−CI	Weight (common)	Weight (random)
Regaieg(2012)	14	0.70	4.0132	14	2.60	5.3218	−1.90	[−5.39; 1.59]	0.8%	4.2%
Youssef (2015)	14	0.80	0.5000	9	1.90	0.7000	−1.10	[−1.63; −0.57]	36.9%	23.6%
Monteiro (2015)	18	−1.06	5.3042	16	2.60	7.6169	−3.66	[−8.12; 0.80]	0.5%	2.7%
Vas1cellos (2015)	10	−4.40	7.4355	10	5.20	8.5012	−9.60	[−16.60; −2.60]	0.2%	1.2%
Racil (2016)	17	−3.20	2.0664	14	0.30	2.3169	−3.50	[−5.06; −1.94]	4.2%	12.8%
Khammassi (2018)	10	−3.90	3.2000	10	−1.10	4.8322	−2.80	[−6.39; 0.79]	0.8%	4.0%
Dias (2017)	33	−1.60	7.6521	34	−0.50	8.9594	−1.10	[−5.09; 2.89]	0.6%	3.3%
Cvetkovic (2018)	10	−0.89	5.7567	14	3.83	7.0522	−4.72	[−9.86; 0.42]	0.4%	2.1%
Martín−García (2017)	19	−0.30	10.5380	28	1.20	8.8123	−1.50	[−7.25; 4.25]	0.3%	1.7%
Cao (2022)	12	−1.80	1.0536	13	1.00	0.9165	−2.80	[−3.58; −2.02]	17.0%	20.9%
Salus (2022)	14	1.20	0.7000	14	2.80	0.7000	−1.60	[−2.12; −1.08]	38.2%	23.6%
**Common effect model**	**171**			**176**			**−1.75**	**[−2.07; −1.43]**	**100.0%**	**−−**
**Random effects model**							**−2.23**	**[−3.01; −1.46]**	**−−**	**100.0%**

Heterogeneity: $I^2 = 60\%$, $\tau^2 = 0.5935$, $p < 0.01$

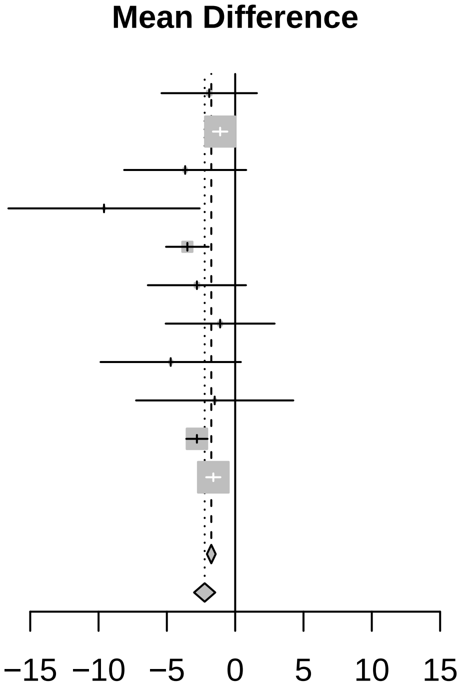

Study	RT-M Total	RT-M Mean	RT-M SD	CON Total	CON Mean	CON SD	MD	95%−CI	Weight (common)	Weight (random)
Lee (2012)	16	−0.60	0.8000	13	2.60	1.0000	−3.20	[−3.87; −2.53]	54.8%	28.6%
Lee (2013)	16	−1.31	1.3800	12	0.13	1.1000	−1.44	[−2.36; −0.52]	29.2%	27.8%
Alberga (2013)	12	2.00	1.6000	7	0.92	1.4000	1.08	[−0.30; 2.46]	13.0%	25.7%
Said (2021)	16	−7.00	5.4051	15	−4.76	2.0800	−2.24	[−5.09; 0.61]	3.0%	17.9%
**Common effect model**	**60**			**47**			**−2.10**	**[−2.60; −1.61]**	**100.0%**	**−−**
**Random effects model**							**−1.44**	**[−3.35; 0.47]**	**−−**	**100.0%**

Heterogeneity: $I^2 = 91\%$, $\tau^2 = 3.2054$, $p < 0.01$

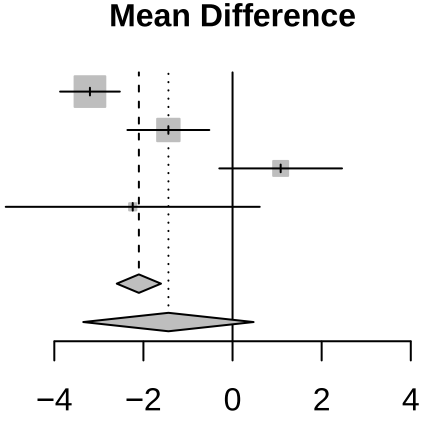

Study	RT-HI Total	RT-HI Mean	RT-HI SD	CON Total	CON Mean	CON SD	Mean Difference	MD	95%−CI
SHAIBI (2006)	11	1.90	1.1000	11	2.10	0.9000	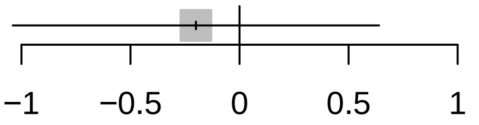	−0.20	[−1.04; 0.64]

Study	COM-M Total	COM-M Mean	COM-M SD	CON Total	CON Mean	CON SD	MD	95%−CI	Weight (common)	Weight (random)
Wong (2008)	12	−2.40	3.6000	12	1.30	4.1000	−3.70	[−6.79; −0.61]	7.1%	16.1%
Jeon (2013)	8	−3.69	3.5750	7	1.47	2.7505	−5.16	[−8.37; −1.95]	6.6%	15.5%
Wong (2017)	15	−6.20	1.2783	15	0.20	1.5906	−6.40	[−7.43; −5.37]	63.2%	28.9%
Duft (2020)	18	−1.06	3.9349	19	1.01	4.4793	−2.07	[−4.78; 0.64]	9.2%	18.2%
Said (2021)	15	−8.82	3.8024	15	−4.76	2.0800	−4.06	[−6.25; −1.87]	14.0%	21.4%
**Common effect model**	**68**			**68**			**−5.40**	**[−6.22; −4.58]**	**100.0%**	**−−**
**Random effects model**							**−4.49**	**[−6.24; −2.73]**	**−−**	**100.0%**

Heterogeneity: $I^2 = 67\%$, $\tau^2 = 2.4954$, $p = 0.02$

Mean Difference

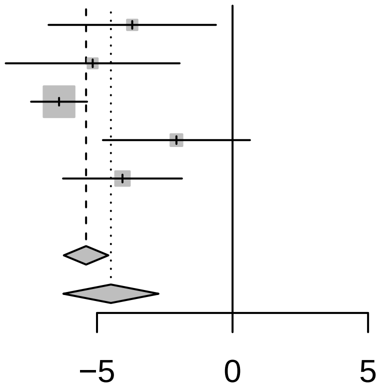

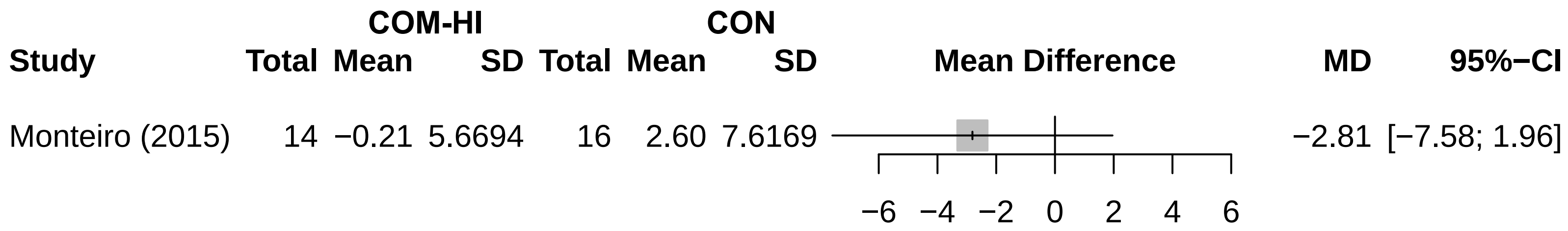
COM-HI
CON
Study
Total
Mean
SD
Total
Mean
SD
Mean Difference
MD
95%−CI
Monteiro (2015)
14
−0.21
5.6694
16
2.60
7.6169
−2.81
[−7.58; 1.96]
−6
−4
−2
0
2
4
6

Study	AE-M Total	AE-M Mean	AE-M SD	AE-HI Total	AE-HI Mean	AE-HI SD	Mean Difference	MD	95%−CI	Weight (common)	Weight (random)
Racil (2016)	16	−1.70	2.8373	17	−3.20	2.0664		1.50	[−0.20; 3.20]	16.4%	28.0%
Dias (2017)	32	−3.80	9.4864	33	−1.60	7.6521		−2.20	[−6.40; 2.00]	2.7%	6.0%
Cao (2022)	11	−1.20	0.8185	12	−1.80	1.0536		0.60	[−0.17; 1.37]	80.9%	66.0%
**Common effect model**	**59**			**62**				**0.67**	**[−0.02; 1.36]**	**100.0%**	**−−**
**Random effects model**								**0.68**	**[−0.37; 1.74]**	**−−**	**100.0%**

Heterogeneity: $I^2 = 27\%$, $\tau^2 = 0.2886$, $p = 0.25$

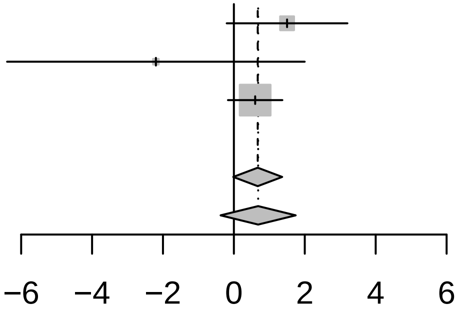

Study	AE-M Total	AE-M Mean	AE-M SD	RT-M Total	RT-M Mean	RT-M SD	MD	95%−CI	Weight (common)	Weight (random)
Lee (2012)	16	−0.04	0.8000	16	−0.60	0.8000	0.56	[0.01; 1.11]	34.8%	24.9%
Lee (2013)	16	−1.31	1.4300	16	−1.31	1.3800	0.00	[−0.97; 0.97]	11.3%	20.3%
Fiorilli (2017)	12	1.68	6.2456	15	2.07	8.2737	−0.39	[−5.87; 5.09]	0.4%	2.2%
Deldin (2019) Boy	14	−1.30	0.9000	14	0.00	0.8000	−1.30	[−1.93; −0.67]	26.9%	24.1%
Deldin (2019) Girl	13	−1.10	0.9000	14	−0.20	0.8000	−0.90	[−1.54; −0.26]	25.8%	24.0%
Said (2021)	15	−7.50	4.9968	16	−7.00	5.4051	−0.50	[−4.16; 3.16]	0.8%	4.5%
**Common effect model**	**86**			**91**			**−0.39**	**[−0.72; −0.07]**	**100.0%**	**−−**
**Random effects model**							**−0.42**	**[−1.26; 0.42]**	**−−**	**100.0%**

Heterogeneity: $I^2 = 78\%$, $\tau^2 = 0.6636$, $p < 0.01$

Mean Difference

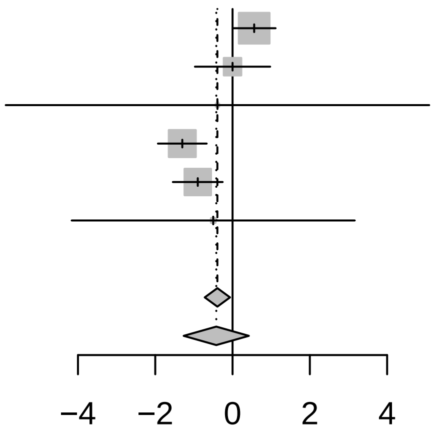

	AE-M			RT-HI					
**Study**	**Total**	**Mean**	**SD**	**Total**	**Mean**	**SD**	**Mean Difference**	**MD**	**95%−CI**
Fiorilli (2017)	12	1.68	6.2456	14	3.73	5.7492		−2.05	[−6.69; 2.59]

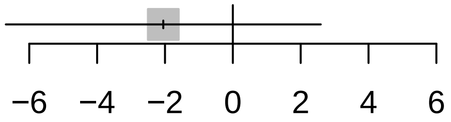

	AE-M			COM-M					
**Study**	**Total**	**Mean**	**SD**	**Total**	**Mean**	**SD**	**Mean Difference**	**MD**	**95%−CI**
Said (2021)	15	−7.50	4.9968	15	−8.82	3.8024	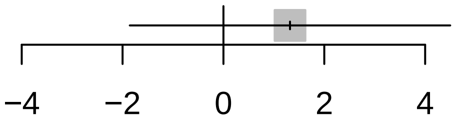	1.32	[−1.86; 4.50]

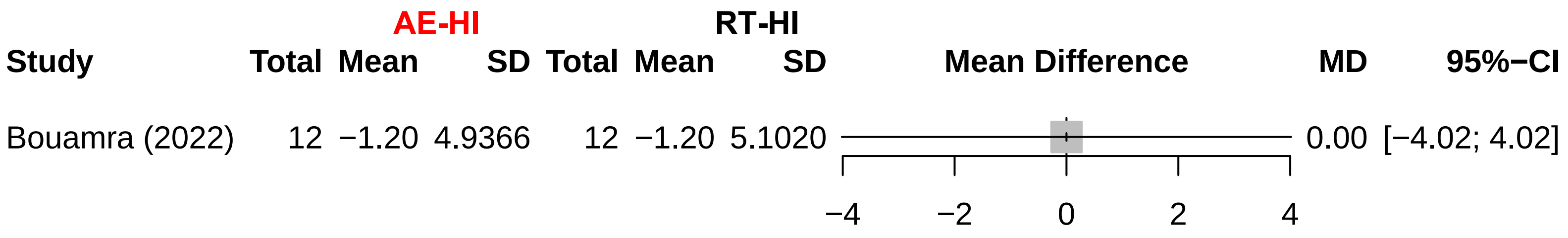
AE-HI
RT-HI
Study
Total
Mean
SD
Total
Mean
SD
Mean Difference
MD
95%−CI
Bouamra (2022)
12
−1.20
4.9366
12
−1.20
5.1020
0.00
[−4.02; 4.02]
−4
−2
0
2
4

Study	AE-HI Total	AE-HI Mean	AE-HI SD	COM-HI Total	COM-HI Mean	COM-HI SD	Mean Difference	MD	95%−CI	Weight (common)	Weight (random)
Monteiro (2015)	18	−1.06	5.3042	14	−0.21	5.6694		−0.85	[−4.70; 3.00]	55.5%	53.0%
Bouamra (2022)	12	−1.20	4.9366	13	−4.30	6.0092		3.10	[−1.20; 7.40]	44.5%	47.0%
**Common effect model**	**30**			**27**				**0.91**	**[−1.96; 3.78]**	**100.0%**	**−−**
**Random effects model**								**1.00**	**[−2.86; 4.87]**	**−−**	**100.0%**

Heterogeneity: $I^2 = 44\%$, $\tau^2 = 3.4675$, $p = 0.18$

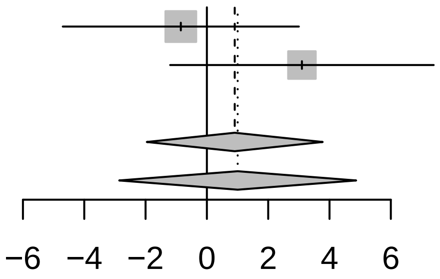

	RT-M			RT-HI					
**Study**	**Total**	**Mean**	**SD**	**Total**	**Mean**	**SD**	**Mean Difference**	**MD**	**95%−CI**
Fiorilli (2017)	15	2.07	8.2737	14	3.73	5.7492		−1.66	[−6.82; 3.50]

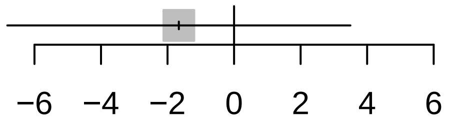

	RT-M			COM-M					
**Study**	**Total**	**Mean**	**SD**	**Total**	**Mean**	**SD**	**Mean Difference**	**MD**	**95%−CI**
Said (2021)	16	−7.00	5.4051	15	−8.82	3.8024	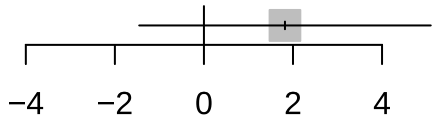	1.82	[−1.45; 5.09]

Study	RT-HI Total	RT-HI Mean	RT-HI SD	COM-HI Total	COM-HI Mean	COM-HI SD	Mean Difference	MD	95%−CI
Bouamra (2022)	12	−1.20	5.1020	13	−4.30	6.0092	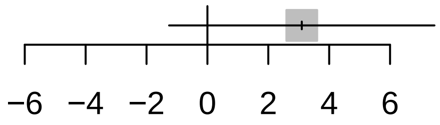	3.10	[−1.26; 7.46]

Figure S5 Network meta-analysis results—Interval plot

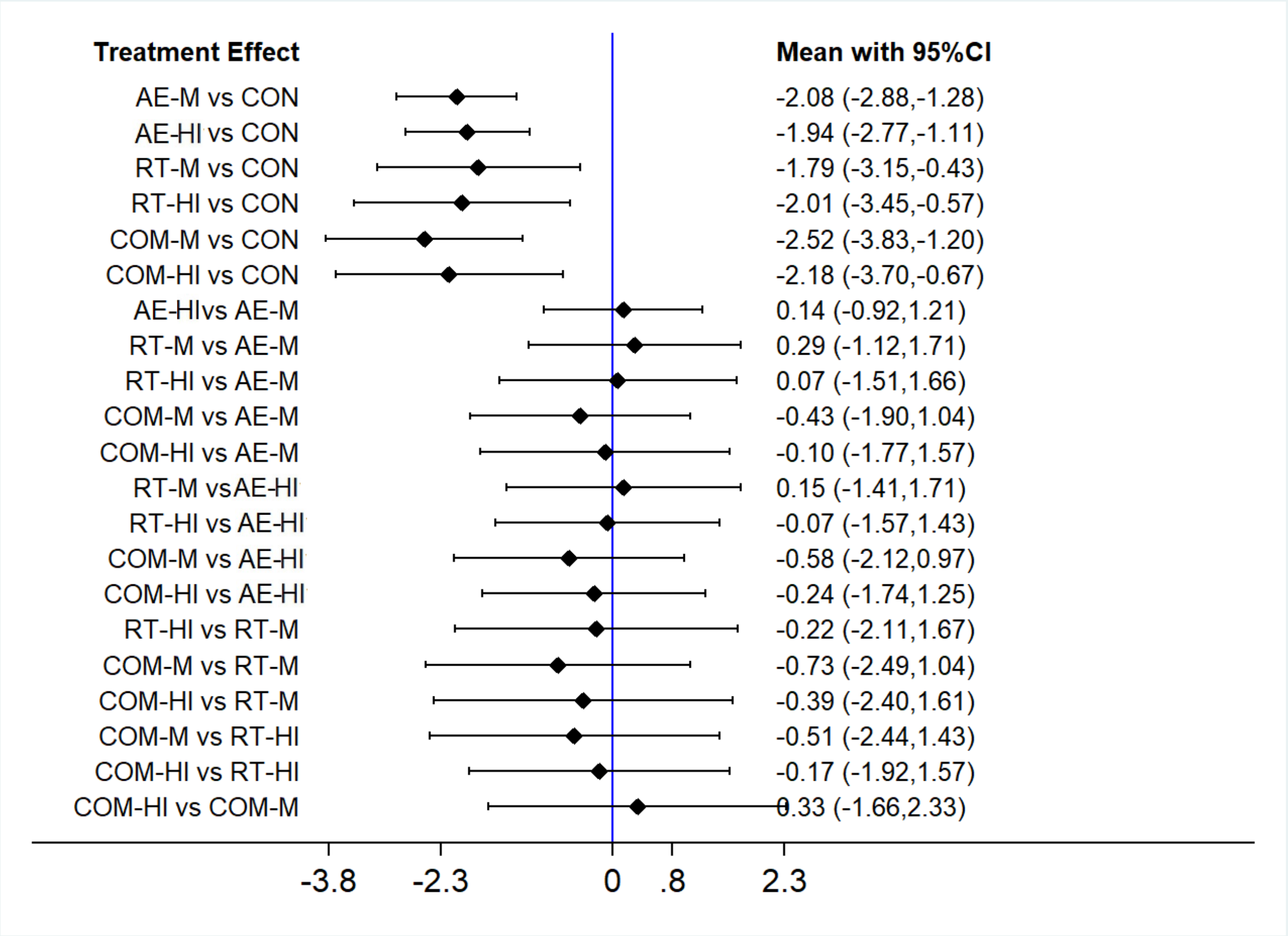

Figure1 Interval Plot for Body Fat Percentage

AE-M, moderate-intensity aerobic exercise
AE-HI, high-intensity aerobic exercise
RT-M, moderate-intensity resistance exercise
RT-HI, high-intensity resistance exercise
COM-M, moderate-intensity combined exercise
COM-HI, high-intensity combined exercise
CON, blank controls

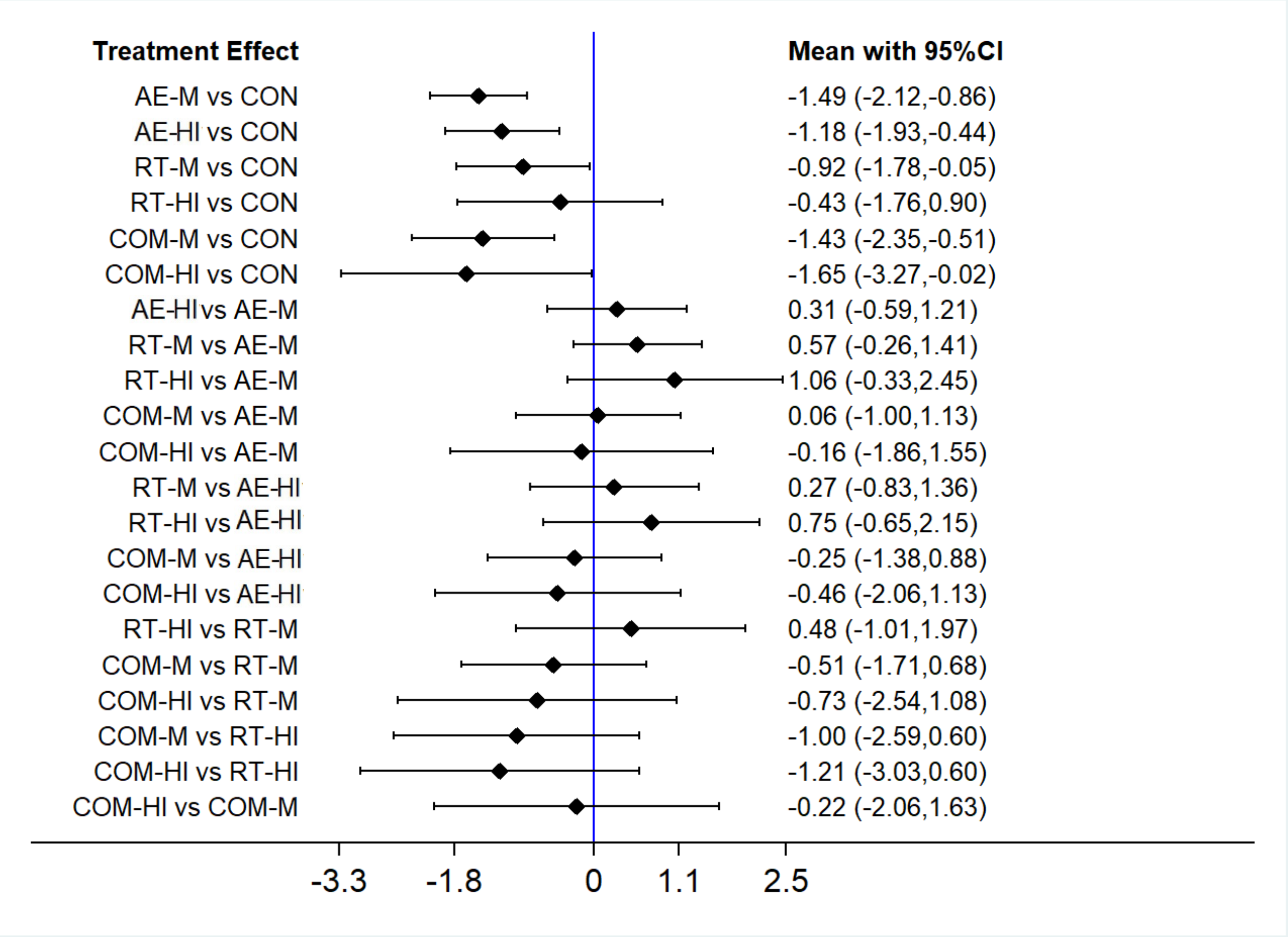

Figure2 Interval Plot for Body Mass Index

AE-M, moderate-intensity aerobic exercise
AE-HI, high-intensity aerobic exercise
RT-M, moderate-intensity resistance exercise
RT-HI, high-intensity resistance exercise
COM-M, moderate-intensity combined exercise
COM-HI, high-intensity combined exercise
CON, blank controls

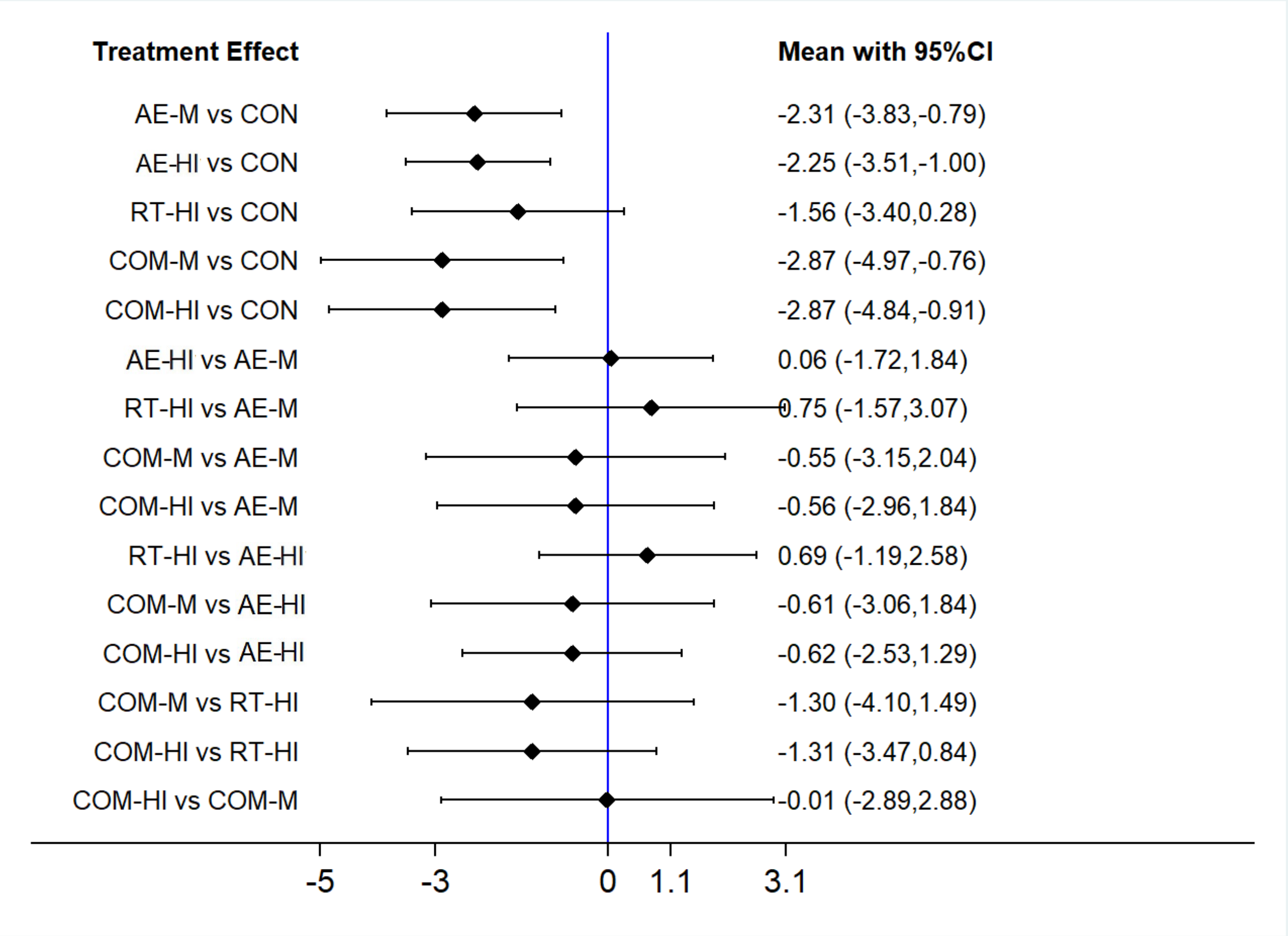

Figure3 Interval Plot for Fat Mass

AE-M, moderate-intensity aerobic exercise
AE-HI, high-intensity aerobic exercise
RT-HI, high-intensity resistance exercise
COM-M, moderate-intensity combined exercise
COM-HI, high-intensity combined exercise
CON, blank controls

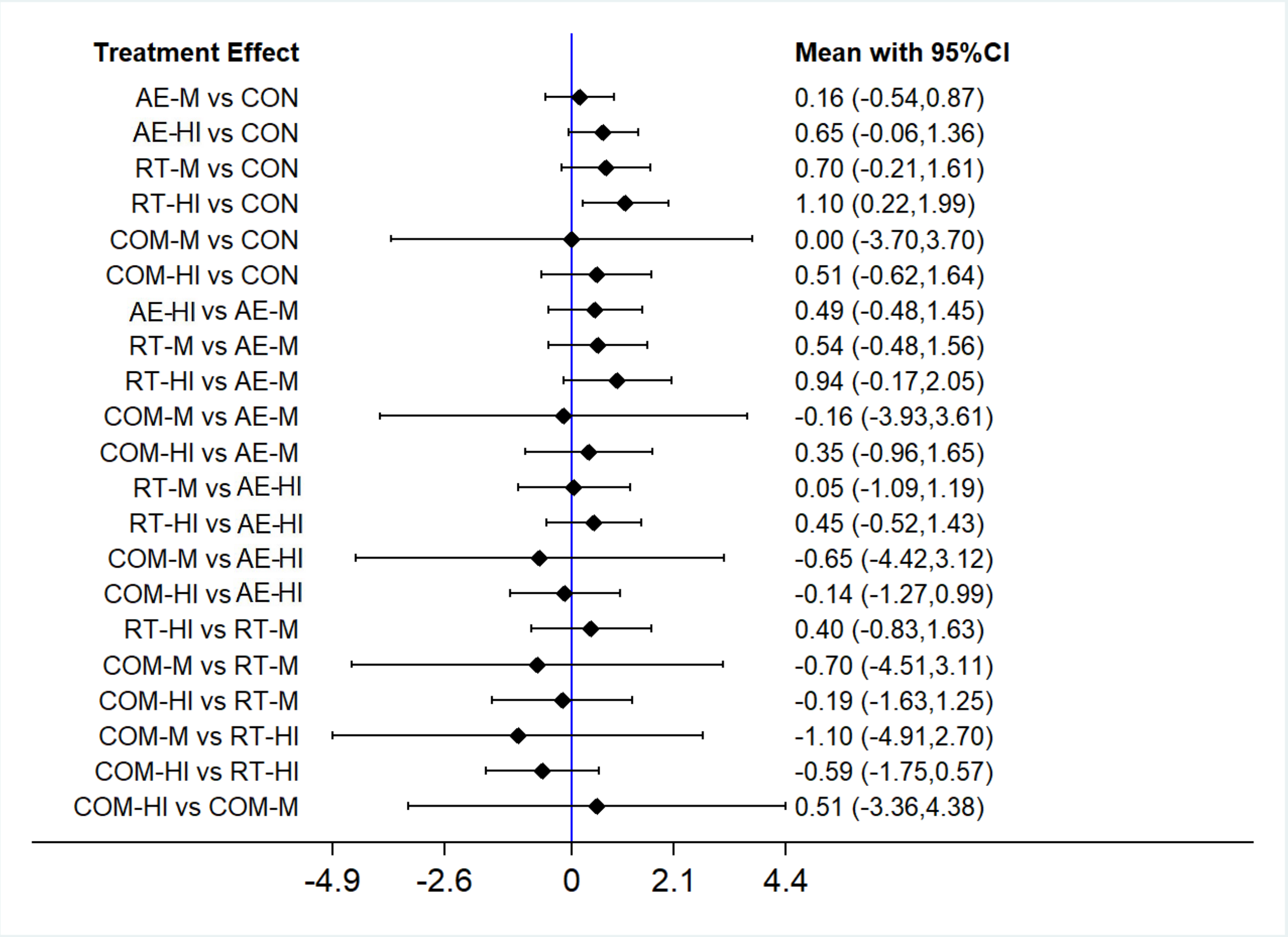

Figure4 Interval Plot for Fat Free Mass

AE-M, moderate-intensity aerobic exercise
AE-HI, high-intensity aerobic exercise
RT-M, moderate-intensity resistance exercise
RT-HI, high-intensity resistance exercise
COM-M, moderate-intensity combined exercise
COM-HI, high-intensity combined exercise
CON, blank controls

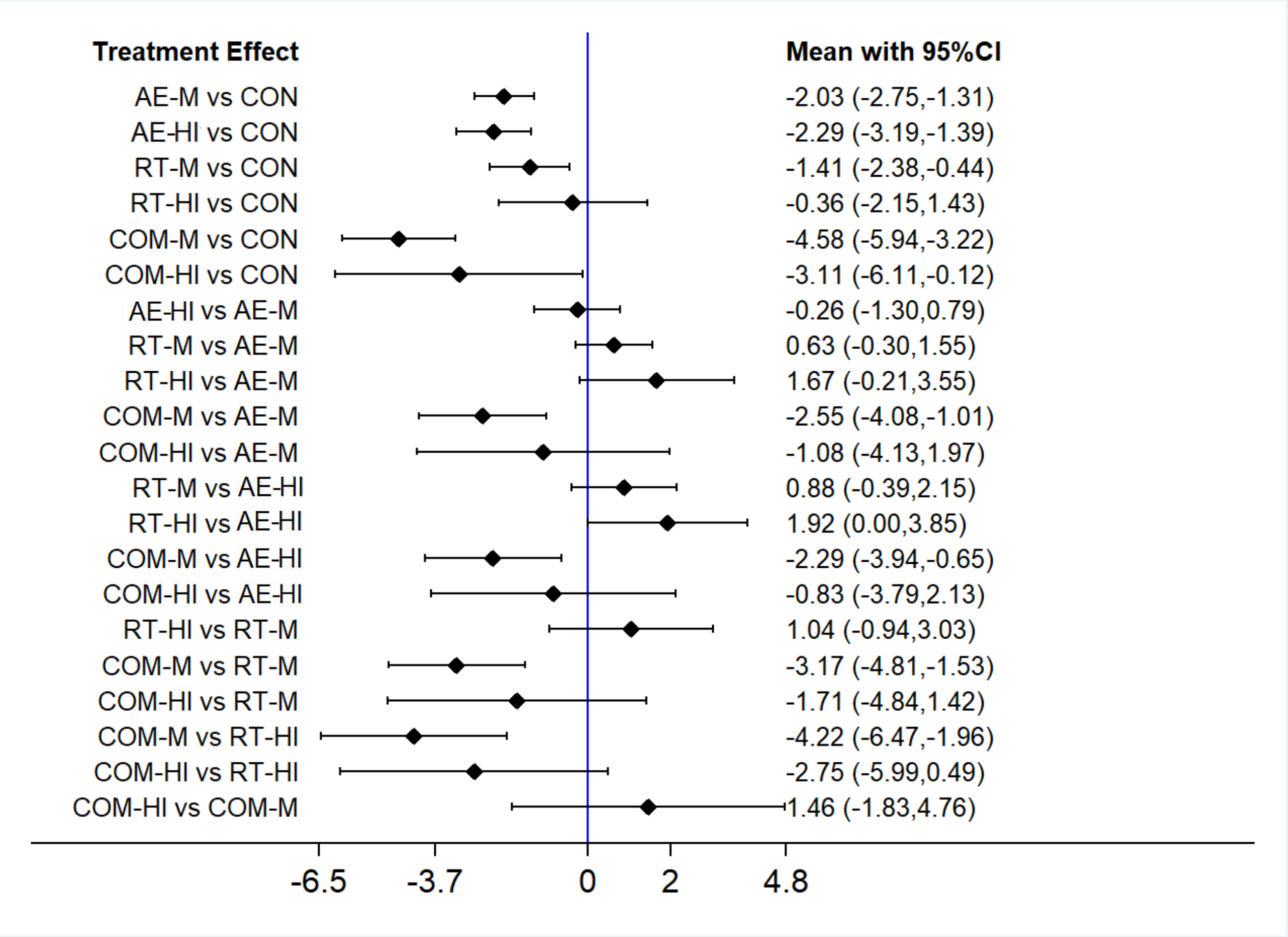

Figure5 Interval Plot for Weight

AE-M, moderate-intensity aerobic exercise
AE-HI, high-intensity aerobic exercise
RT-M, moderate-intensity resistance exercise
RT-HI, high-intensity resistance exercise
COM-M, moderate-intensity combined exercise
COM-HI, high-intensity combined exercise
CON, blank controls

Figure S6 Loop Inconsistency Plots

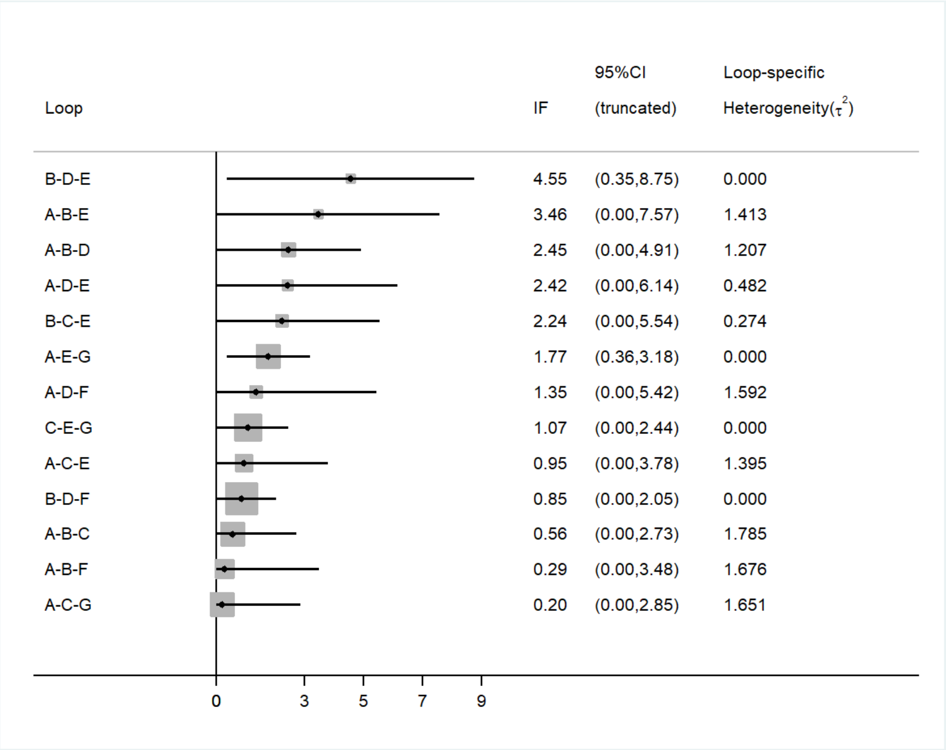

Figure 1. Loop inconsistency plot for body fat percentage

A, blank controls
B, moderate-intensity aerobic exercise
C, high-intensity aerobic exercise
D, moderate-intensity resistance exercise
E, high-intensity resistance exercise
F, moderate-intensity combined exercise
G, high-intensity combined exercise

Loop	IF	95%CI (truncated)	Loop-specific Heterogeneity(τ^2)
C-D-E-F	2.88	(0.35,5.41)	0.000
C-E-G	1.93	(0.04,3.82)	0.000
B-C-F	1.89	(0.23,3.55)	0.000
A-B-D	1.41	(0.00,3.16)	0.390
A-C-E	1.29	(0.00,4.14)	0.855
A-D-E	1.01	(0.00,3.46)	0.278
A-B-C	0.73	(0.00,3.04)	0.561
A-C-F	0.70	(0.00,3.34)	0.974
A-B-F	0.61	(0.00,4.12)	0.768
A-E-G	0.60	(0.00,2.32)	0.000
A-B-E	0.59	(0.00,5.85)	0.675
B-D-E	0.51	(0.00,3.06)	0.094
A-D-F	0.44	(0.00,2.92)	0.550
A-C-G	0.43	(0.00,3.18)	0.645
B-D-F	0.30	(0.00,1.62)	0.095
B-C-E	0.22	(0.00,2.19)	0.000

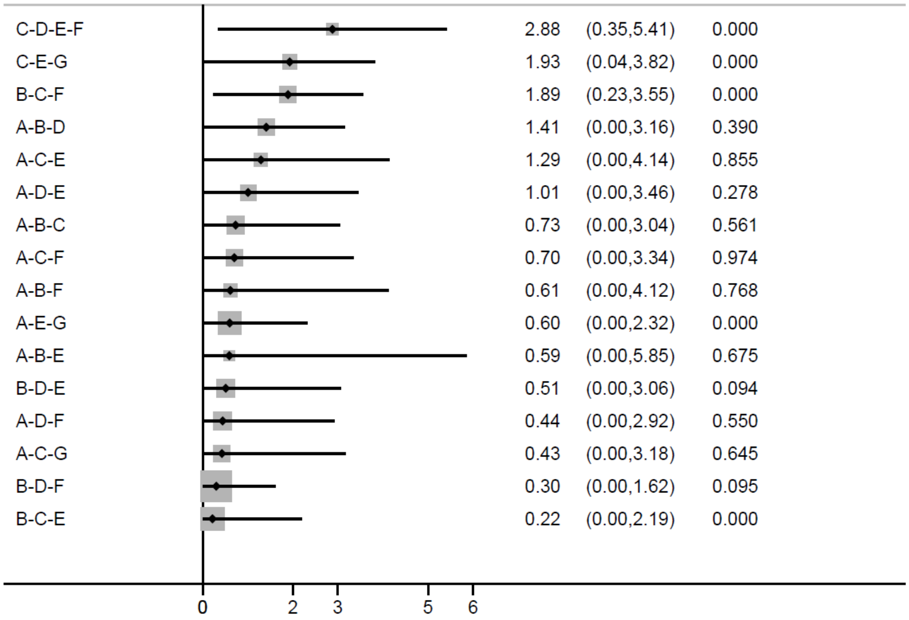

Figure 2. Loop inconsistency plot for body mass index

A, blank controls
B, moderate-intensity aerobic exercise
C, high-intensity aerobic exercise
D, moderate-intensity resistance exercise
E, high-intensity resistance exercise
F, moderate-intensity combined exercise
G, high-intensity combined exercise

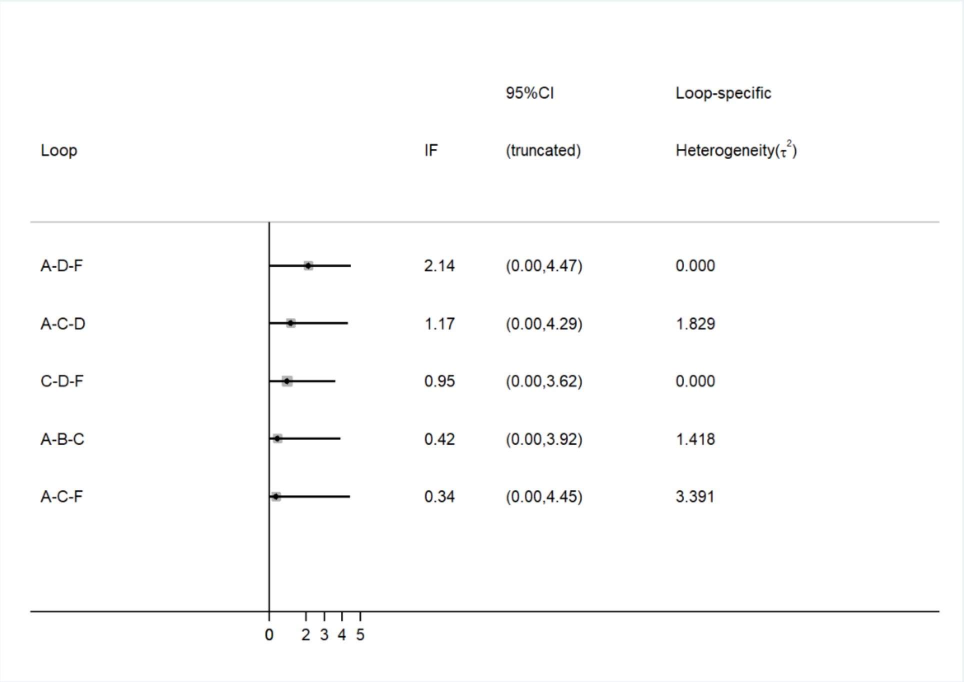

Figure 3. Loop inconsistency plot for fat mass

A, blank controls
B, moderate-intensity aerobic exercise
C, high-intensity aerobic exercise
D, high-intensity resistance exercise
F, high-intensity combined exercise

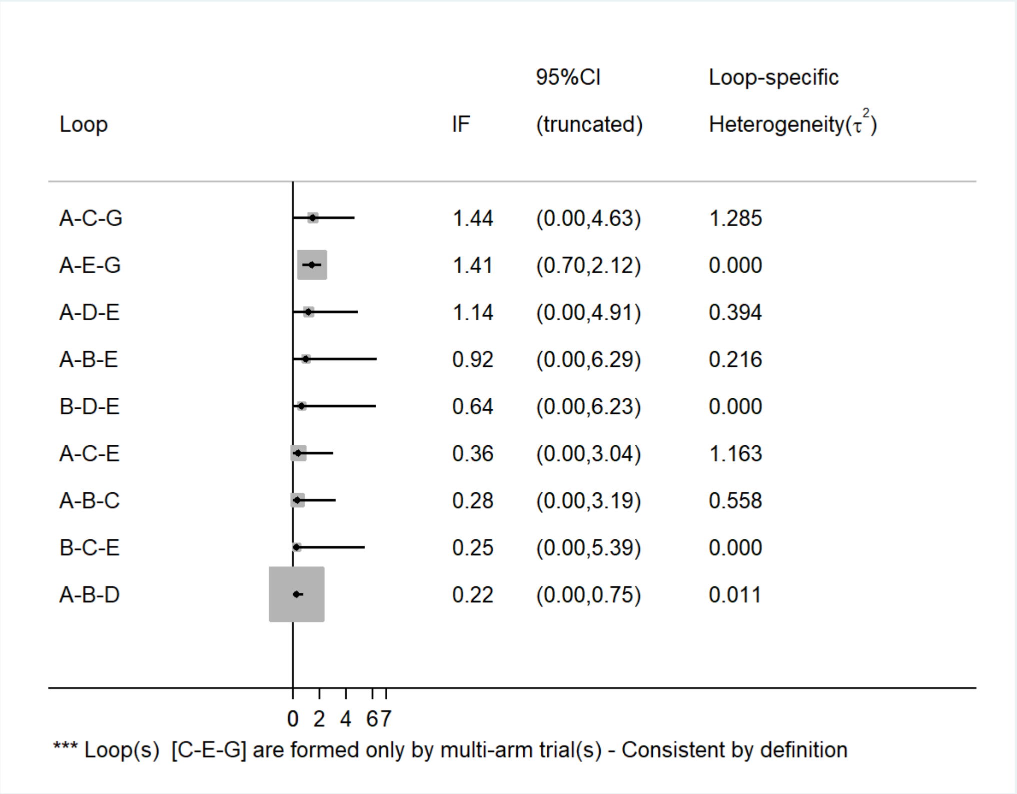

Figure 4. Loop inconsistency plot for fat free mass

A, blank controls
B, moderate-intensity aerobic exercise
C, high-intensity aerobic exercise
D, moderate-intensity resistance exercise
E, high-intensity resistance exercise
G, high-intensity combined exercise

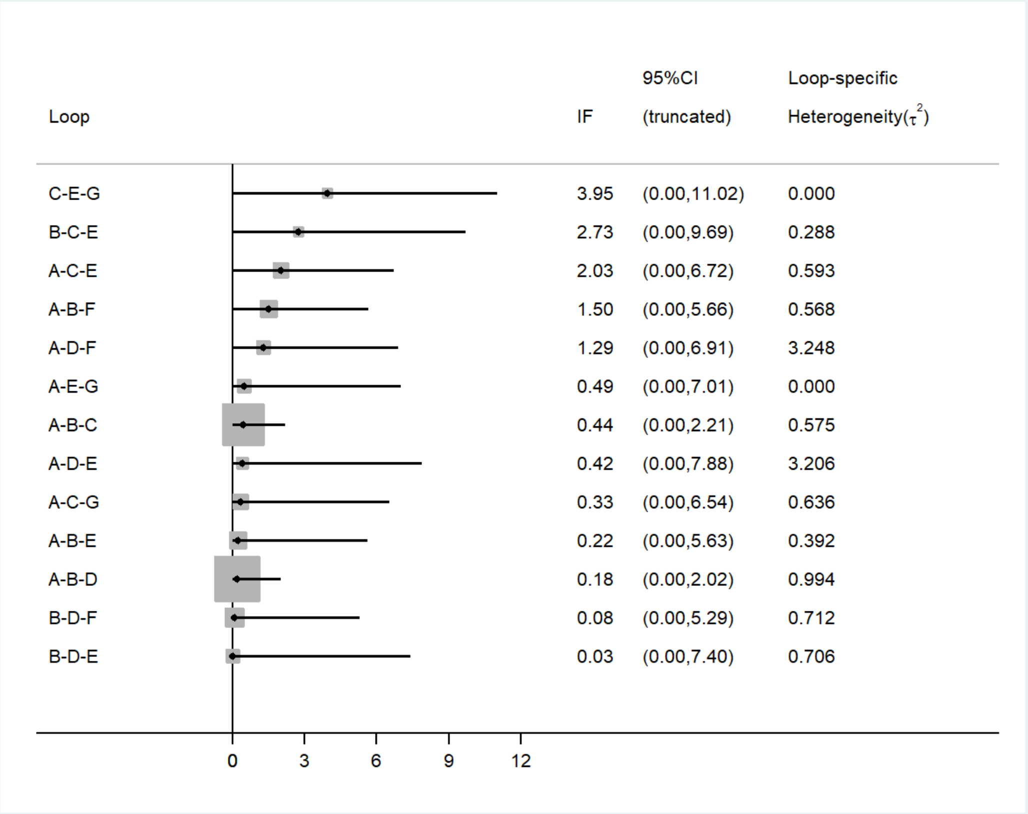

Figure 5. Loop inconsistency plot for weight

A, blank controls
B, moderate-intensity aerobic exercise
C, high-intensity aerobic exercise
D, moderate-intensity resistance exercise
E, high-intensity resistance exercise
F, moderate-intensity combined exercise
G, high-intensity combined exercise

Figure S7 Contribution Plots of Direct and Indirect Evidence

Direct comparisons in the network (columns); Network meta-analysis estimates (rows)

	AvsB	AvsC	AvsD	AvsE	AvsF	AvsG	BvsC	BvsD	BvsE	BvsF	CvsE	CvsG	DvsE	DvsF	EvsG
Mixed estimates															
AvsB	28.5	5.0	7.3	10.4	4.6	1.0	13.6	3.1	2.0	9.7	7.2	1.3	0.9	5.1	0.3
AvsC	6.0	14.7	1.8	28.0	1.0	2.8	7.6	0.5	0.7	1.7	27.8	4.4	0.7	0.7	1.6
AvsD	14.0	2.9	17.8	6.5	4.8	0.6	6.3	7.6	0.9	13.7	2.8	0.6	2.8	18.5	
AvsE	5.9	13.3	1.9	40.3	1.0	3.1	6.6	0.4	1.2	1.5	19.6	0.3	1.1	0.4	3.4
AvsF	17.6	3.3	9.5	6.9	7.2	0.7	8.2	1.2	1.2	25.8	4.2	0.8	1.4	12.0	0.1
AvsG	4.3	10.1	1.3	23.6	0.7	5.3	5.1	0.3	0.6	1.1	6.3	21.5	0.6	0.4	18.5
BvsC	17.1	7.9	4.2	14.6	2.8	1.5	16.6	2.0	2.0	6.1	16.8	2.6	1.2	3.4	1.0
BvsD	10.3	1.3	12.9	2.2	1.1	0.2	5.3	11.1	0.8	23.7	3.5	0.5	2.3	24.7	0.2
BvsE	17.4	4.8	4.2	18.3	2.8	1.3	14.3	2.1	2.2	[illegible]	18.0	1.1	1.4	3.5	2.4
BvsF	7.7	1.1	5.6	2.0	5.4	0.2	3.8	5.7	0.6	51.5	[illegible]	0.4	1.0	12.4	0.1
CvsE	1.7	5.4	0.3	8.0	0.3	0.3	3.1	0.2	0.5	0.7	65.2	6.9	0.3	0.4	6.5
CvsG	1.1	3.1	0.3	0.4	0.2	4.3	1.7	0.1	0.1	0.4	25.0	37.4	0.1	0.2	25.6
DvsE	7.1	4.7	11.9	16.0	2.9	1.2	8.0	5.7	1.3	10.6	12.1	0.6	2.6	13.6	1.8
DvsF	5.8	0.6	11.1	0.9	3.7	0.1	3.1	8.7	0.5	18.1	2.2	0.3	1.9	42.9	0.2
EvsG	0.3	1.3		5.9		4.3	0.8	0.1	0.3	0.2	27.6	29.7	0.2	0.1	29.1
Indirect estimates															
BvsG	14.7	4.9	3.6	12.3	2.4	3.6	12.8	1.7	1.8	5.3	0.4	18.1	1.1	2.9	14.6
CvsD	6.2	6.7	11.3	12.2	2.7	1.3	8.9	5.6	1.0	10.6	14.7	2.2	2.4	13.3	0.9
CvsF	9.3	6.6	5.9	12.1	4.7	1.3	10.7	1.2	1.3	20.1	13.9	2.1	1.4	8.5	0.9
DvsG	5.9	4.6	10.0	10.8	2.5	2.9	7.1	4.8	0.9	9.1	2.3	13.9	2.1	11.5	11.6
EvsF	10.1	4.4	6.1	15.5	4.8	1.1	9.5	1.1	1.5	20.0	13.1	0.7	1.6	8.8	1.8
FvsG	8.6	4.4	5.2	10.6	4.1	2.9	8.6	1.0	1.2	17.3	1.3	14.3	1.3	7.5	11.7
**Entire network**	9.8	5.3	6.8	12.5	3.0	1.9	8.3	3.1	1.1	12.3	11.7	7.4	1.5	9.2	6.1
**Included studies**	11	12	3	2	5	2	3	3	1	1	2	3	1	1	2

Figure 1. Contribution plots of direct and indirect evidence for body fat percentage

A, blank controls
B, moderate-intensity aerobic exercise
C, high-intensity aerobic exercise
D, moderate-intensity resistance exercise
E, high-intensity resistance exercise
F, moderate-intensity combined exercise
G, high-intensity combined exercise

Direct comparisons in the network

Network meta-analysis estimates	AvsB	AvsC	AvsD	AvsE	AvsF	AvsG	BvsC	BvsD	BvsE	BvsF	CvsE	CvsF	CvsG	DvsE	DvsF	EvsG
Mixed estimates																
AvsB	24.7	5.6	15.7	6.8	5.6	0.4	7.7	18.4	2.6	5.4	2.5	1.0	0.6	1.5	1.2	0.2
AvsC	9.8	22.1	6.6	13.2	3.3	1.4	16.0	5.8	0.7	0.2	11.1	4.7	2.5	0.3	1.2	1.1
AvsD	17.5	4.3	21.6	[illegible]	5.3	0.4	5.2	25.6	1.8	1.0	1.8	1.3	0.4	2.1	5.7	0.1
AvsE	3.0	3.4	2.3	73.1	0.9	2.0	1.6	0.7	2.3	0.2	5.1	0.5	0.5	1.7	0.1	2.5
AvsF	14.7	5.1	12.7	5.2	13.7	0.4	2.9	1.3	1.5	17.9	2.3	5.0	0.5	1.2	15.2	0.2
AvsG	2.6	4.7	1.9	26.9	0.8	19.6	3.1	1.2	0.7	0.1	0.1	0.9	8.8	0.5	0.2	28.0
BvsC	12.4	14.9	7.5	6.0	1.8	0.9	20.9	[illegible]	1.7	4.7	7.8	5.1	1.7	1.0	2.1	0.9
BvsD	7.9	1.4	9.9	0.6	0.2	0.1	2.8	61.3	0.9	5.8	0.7	0.5	0.2	1.1	6.5	0.1
BvsE	16.1	2.3	10.1	31.4	3.5	0.7	6.3	12.8	3.0	3.7	4.3	1.0	0.7	1.9	0.8	1.4
BvsF	8.5	0.1	1.4	0.9	10.6	·	4.6	21.0	0.9	27.1	0.2	5.0	·	0.1	19.5	·
CvsE	6.4	16.4	4.2	29.4	2.3	·	12.3	4.4	1.8	0.3	12.0	3.6	2.3	1.2	1.1	2.3
CvsF	4.6	12.3	5.3	5.6	8.6	0.7	14.3	5.3	0.7	14.3	6.3	7.6	1.4	0.7	11.4	0.7
CvsG	5.2	12.6	3.5	9.9	1.8	13.2	9.4	3.3	1.0	0.2	8.0	2.7	8.2	0.6	0.8	19.5
DvsE	11.2	1.5	14.4	29.8	3.4	0.7	4.5	18.6	2.4	0.6	3.7	1.2	0.5	2.3	4.0	1.2
DvsF	2.0	1.2	8.6	0.3	9.7	·	2.2	25.5	0.2	21.0	0.7	4.3	0.1	0.9	23.0	0.1
EvsG	0.7	2.7	0.3	24.1	0.3	20.2	2.2	0.8	0.9	0.1	3.8	0.6	9.4	0.7	0.2	32.8
Indirect estimates																
BvsG	13.3	0.4	8.3	12.8	2.8	12.0	6.6	10.4	2.0	3.2	1.5	1.2	5.9	1.2	0.8	17.5
CvsD	6.1	13.1	11.5	5.3	1.6	0.8	15.9	23.8	0.9	0.9	6.8	4.5	1.5	1.4	5.2	0.8
DvsG	9.1	0.2	11.9	12.3	2.7	11.3	5.0	16.1	1.5	0.6	1.1	1.3	5.5	1.6	3.5	16.4
EvsF	9.3	2.2	8.1	28.6	9.6	0.6	2.9	1.3	2.1	13.0	3.9	3.9	0.6	1.6	11.0	1.2
FvsG	7.6	0.5	6.8	12.0	8.0	10.9	3.6	1.5	1.3	11.1	1.4	3.7	5.4	1.0	9.3	15.8
**Entire network**	9.3	5.9	8.4	15.7	4.7	4.9	7.3	11.8	1.5	6.1	4.1	2.9	2.9	1.2	5.8	7.3
**Included studies**	12	10	4	1	6	1	2	6	1	1	1	1	2	1	1	1

Figure 2. Contribution plots of direct and indirect evidence for body mass index

A, blank controls
B, moderate-intensity aerobic exercise
C, high-intensity aerobic exercise
D, moderate-intensity resistance exercise
E, high-intensity resistance exercise
F, moderate-intensity combined exercise
G, high-intensity combined exercise

Direct comparisons in the network

Network meta-analysis estimates	AvsB	AvsC	AvsD	AvsE	AvsF	BvsC	CvsD	CvsF	DvsF
**Mixed estimates**									
AvsB	16.0	5.3	21.6		2.8	29.7	21.7	2.7	0.2
AvsC	2.4	9.2	37.8		5.0	2.4	38.1	4.7	0.3
AvsD	2.9	11.0	52.2		6.0	2.9	19.1	5.2	0.8
AvsE			0.1	99.9					
AvsF	1.6	6.0	25.0		11.9	1.6	21.3	28.9	3.8
BvsC	19.6	3.5	14.2		1.9	44.6		1.8	0.1
CvsD	0.4	1.7	2.9		0.8	0.4	91.9	1.3	0.5
CvsF	0.6	2.5	9.4		12.5	0.6	15.8	52.1	6.4
DvsF	0.2	0.9	8.4		9.5	0.2	38.4	37.3	4.9
**Indirect estimates**									
BvsD	15.0	1.8	12.2		1.0	33.3	35.8	0.7	0.4
BvsE	11.0	3.6	14.8	31.4	2.0	20.4	14.9	1.8	0.1
BvsF	14.9	0.9	4.2		9.7	32.5	0.1	33.5	4.2
CvsE	1.6	6.0	24.5	35.3	3.2	1.6	24.7	3.0	0.2
DvsE	1.7	6.4	30.3	41.9	3.5	1.7	11.1	3.0	0.5
EvsF	1.1	4.2	17.3	30.8	8.3	1.1	14.7	20.0	2.6
**Entire network**	6.2	4.3	19.0	16.4	5.4	11.9	22.1	13.0	1.6
**Included studies**	5	7	2	3	2	2	2	3	2

Figure 3. Contribution plots of direct and indirect evidence for fat mass

A, blank controls
B, moderate-intensity aerobic exercise
C, high-intensity aerobic exercise
D, high-intensity resistance exercise
E, moderate-intensity combined exercise
F, high-intensity combined exercise

Direct comparisons in the network

Network meta-analysis estimates	AvsB	AvsC	AvsD	AvsE	AvsF	AvsG	BvsC	BvsD	BvsE	CvsE	CvsG	DvsE	EvsG
Mixed estimates													
AvsB	40.6	0.1	28.2			0.8	0.6	28.4	0.1	0.2	0.7	0.2	0.1
AvsC	0.7	3.9	0.7	2.1		43.0	0.8	0.3	0.2	5.0	40.6	0.4	2.3
AvsD	22.6	0.1	52.0			0.6	0.3	23.1	0.1	0.3	0.6	0.3	0.1
AvsE	0.6	2.7	0.6	2.3		30.9	0.6	0.2	0.2	30.5	27.2	0.4	3.7
AvsF					99.8								
AvsG	0.7	3.6	0.6	2.0		82.7	0.8	0.3	0.2	2.0	6.3	0.4	0.5
BvsC	17.2	2.3	11.8	1.2		25.5	0.8	[illegible]	0.2	3.1	24.1	0.3	1.4
BvsD	21.8		22.0			0.2	0.3	55.1	0.1	0.1	0.2	0.1	
BvsE	14.2	1.8	9.7	1.6		20.5	0.6	10.1	0.2		18.0	0.4	2.5
CvsE	0.2	0.4	0.3	1.9		2.0	0.1	0.1	0.2	82.9		0.4	4.9
CvsG	0.6	3.3	0.6	1.7		6.1	0.7	0.2	0.1	6.6	75.4	0.3	4.5
DvsE	7.3	1.9	17.2	1.6		21.1	0.5	8.0	0.2	20.9	18.5	0.4	2.5
EvsG	0.4	1.7	0.5	2.1		4.7	0.4	0.1	0.2	43.1	41.0	0.4	5.4
Indirect estimates													
BvsF	23.9		16.6		41.1	0.4	0.4	16.7	0.1	0.1	0.4	0.1	
BvsG	23.1	1.6	16.0	0.9		36.6	0.7	16.2	0.2	1.0	3.3	0.3	0.3
CvsD	8.9	2.4	21.0	1.3		26.2	0.6	9.7	0.1	3.2	24.8	0.4	1.4
CvsF	0.5	2.6	0.4	1.4	33.5	28.6	0.5	0.2	0.1	3.3	27.0	0.3	1.5
DvsF	12.9		29.7		43.0	0.3	0.2	13.2		0.2	0.3	0.2	
DvsG	12.2	1.6	28.5	0.9		38.2	0.5	12.9	0.1	1.1	3.3	0.3	0.3
EvsF	0.4	2.0	0.4	1.7	27.1	22.5	0.4	0.1	0.1	22.2	19.8	0.3	2.7
FvsG	0.3	1.9	0.3	1.0	47.2	43.6	0.4	0.1	0.1	1.1	3.4	0.2	0.3
**Entire network**	9.8	1.8	12.1	1.2	12.5	22.1	0.5	9.0	0.1	11.7	17.1	0.3	1.7
**Included studies**	6	5	2	2	1	1	1	2	1	2	2	1	2

Figure 4. Contribution plots of direct and indirect evidence for fat free mass

A, blank controls
B, moderate-intensity aerobic exercise
C, high-intensity aerobic exercise
D, moderate-intensity resistance exercise
E, high-intensity resistance exercise
F, moderate-intensity combined exercise
G, high-intensity combined exercise

Direct comparisons in the network

Network meta-analysis estimates	AvsB	AvsC	AvsD	AvsE	AvsF	AvsG	BvsC	BvsD	BvsE	BvsF	CvsE	CvsG	DvsE	DvsF	EvsG
Mixed estimates															
AvsB	60.8	10.6	4.1	1.7	2.1	0.1	11.1	5.4	0.8	1.2	0.3	0.2	0.5	0.8	0.1
AvsC	18.9	49.0	1.3	2.6	0.6	0.6	21.1	1.6	0.2	0.4	1.7	1.1	0.1	0.3	0.6
AvsD	31.5	5.5	10.0		2.8	0.1	5.8	37.8	0.4	0.1	0.1	0.1	1.3	2.6	
AvsE	4.4	3.6	0.6	77.5	0.2	0.8	0.7	1.2	2.5	0.1	3.3	1.1	2.0	0.1	1.9
AvsF	16.7	2.9	2.9	0.7	41.9		3.1	7.7	0.2	12.3	0.1	0.1	0.4	11.0	
AvsG	6.5	15.4	0.5	16.1	0.2	14.7	6.5	0.2	0.4	0.1	0.1	21.7	0.4	0.1	17.0
BvsC	30.5	32.4	2.1	0.8	1.0	0.4	25.9	[illegible]	0.5	0.6	1.2	0.8	0.3	0.4	0.4
BvsD	12.3	2.1	11.2	1.1	2.2		2.3	62.7	0.2	1.0	0.1	0.1	1.5	3.2	0.1
BvsE	33.2	4.3	2.1	40.3	1.1	0.4	6.9	3.8	1.8	0.7	1.9	0.7	1.4	0.4	1.1
BvsF	26.7	4.6	0.4	0.6	32.3	0.1	4.9	9.8	0.4	10.7	0.1	0.1		9.4	
CvsE	9.5	28.5	0.5	38.7	0.3	0.1	12.7	1.7	1.4	0.2	2.8	1.3	1.1	0.1	1.3
CvsG	6.7	18.9	0.4	13.1	0.2	13.1	8.3	0.9	0.5	0.1	1.3	20.8	0.4	0.1	15.3
DvsE	20.2	2.4	6.7	30.8	1.8	0.3	4.3	25.8	1.3	0.1	1.4	0.5	1.7	1.8	0.8
DvsF	15.4	2.7	6.5	1.1	25.7		2.8	26.7	0.2	8.3	0.1		0.9	9.6	
EvsG	3.4	11.7	0.1	27.8	0.1	12.5	5.3	0.9	1.0	0.1	1.7	18.6	0.8		16.0
Indirect estimates															
BvsG	25.5	6.1	1.7	11.0	0.8	10.8	10.4	2.5	0.7	0.5	0.3	16.2	0.5	0.3	12.5
CvsD	14.5	23.4	7.3		1.8	0.3	16.6	31.1	0.2	0.1	0.9	0.6	1.0	2.0	0.3
CvsF	0.1	28.3	1.2	1.1	28.6	0.3	15.2	6.4		8.7	1.0	0.7	0.2	7.8	0.3
DvsG	15.0	5.6	5.7	8.2	1.5	8.4	7.2	22.7	0.5		0.1	12.7	1.0	1.5	9.8
EvsF	8.5	0.1	1.6	36.2	26.4	0.4	2.3	4.3	1.3	7.8	1.6	0.6	1.2	7.0	0.9
FvsG	4.4	8.1	1.2	9.6	21.1	9.1	5.6	4.1	0.4	6.3	0.1	13.5	0.4	5.7	10.5
**Entire network**	16.4	12.0	3.3	15.0	9.5	3.9	8.3	12.4	0.7	2.9	0.9	6.0	0.8	3.1	4.8
**Included studies**	13	11	4	1	5	1	3	6	1	1	1	2	1	1	1

Figure 5. Contribution plots of direct and indirect evidence for weight

A, blank controls
B, moderate-intensity aerobic exercise
C, high-intensity aerobic exercise
D, moderate-intensity resistance exercise
E, high-intensity resistance exercise
F, moderate-intensity combined exercise
G, high-intensity combined exercise

Figure S8 SUCRA values for each intervention

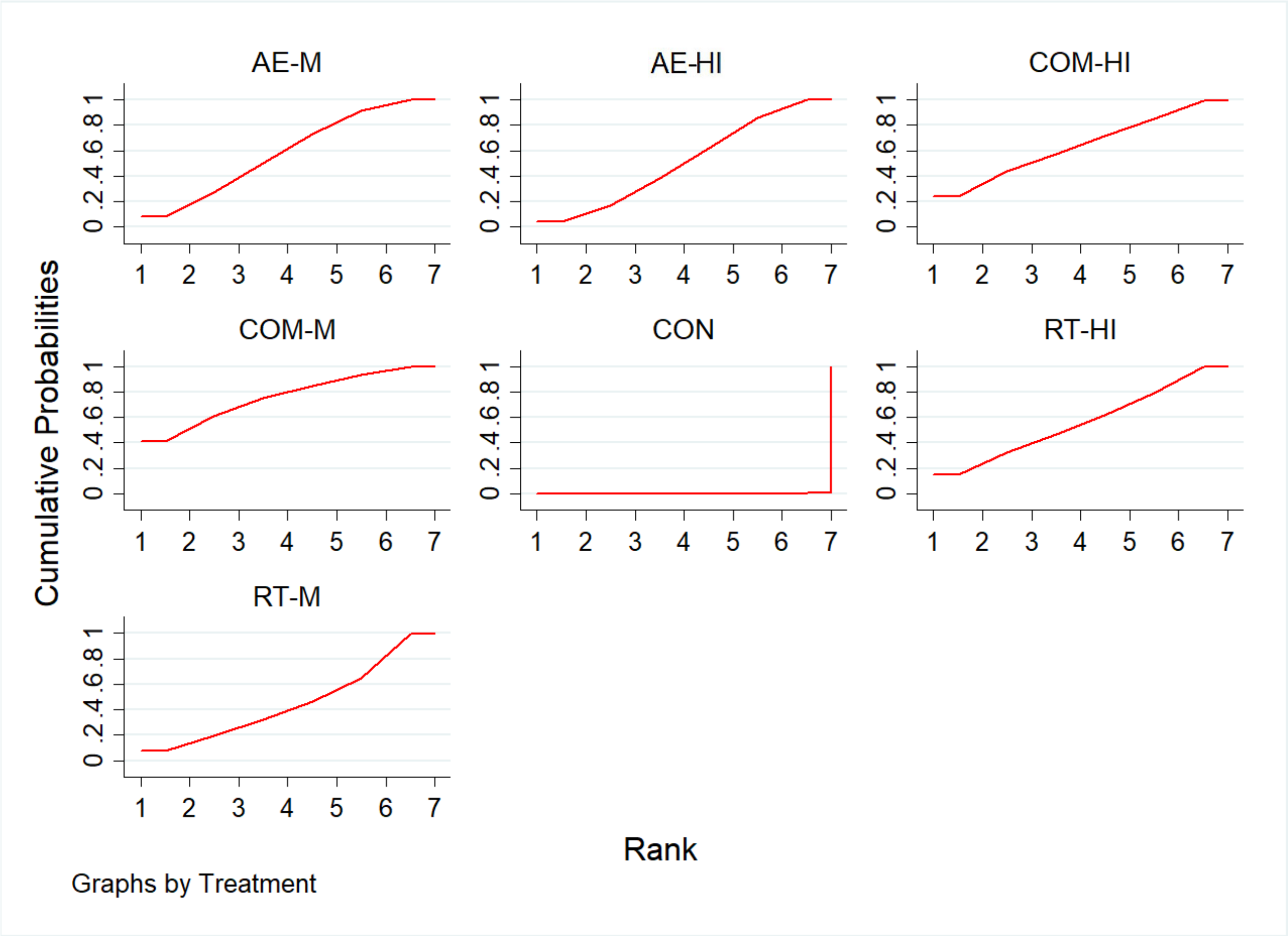

Figure 1. SUCRA values for body fat percentage

AE-M, moderate-intensity aerobic exercise
AE-HI, high-intensity aerobic exercise
RT-M, moderate-intensity resistance exercise
RT-HI, high-intensity resistance exercise
COM-M, moderate-intensity combined exercise
COM-HI, high-intensity combined exercise
CON, blank controls

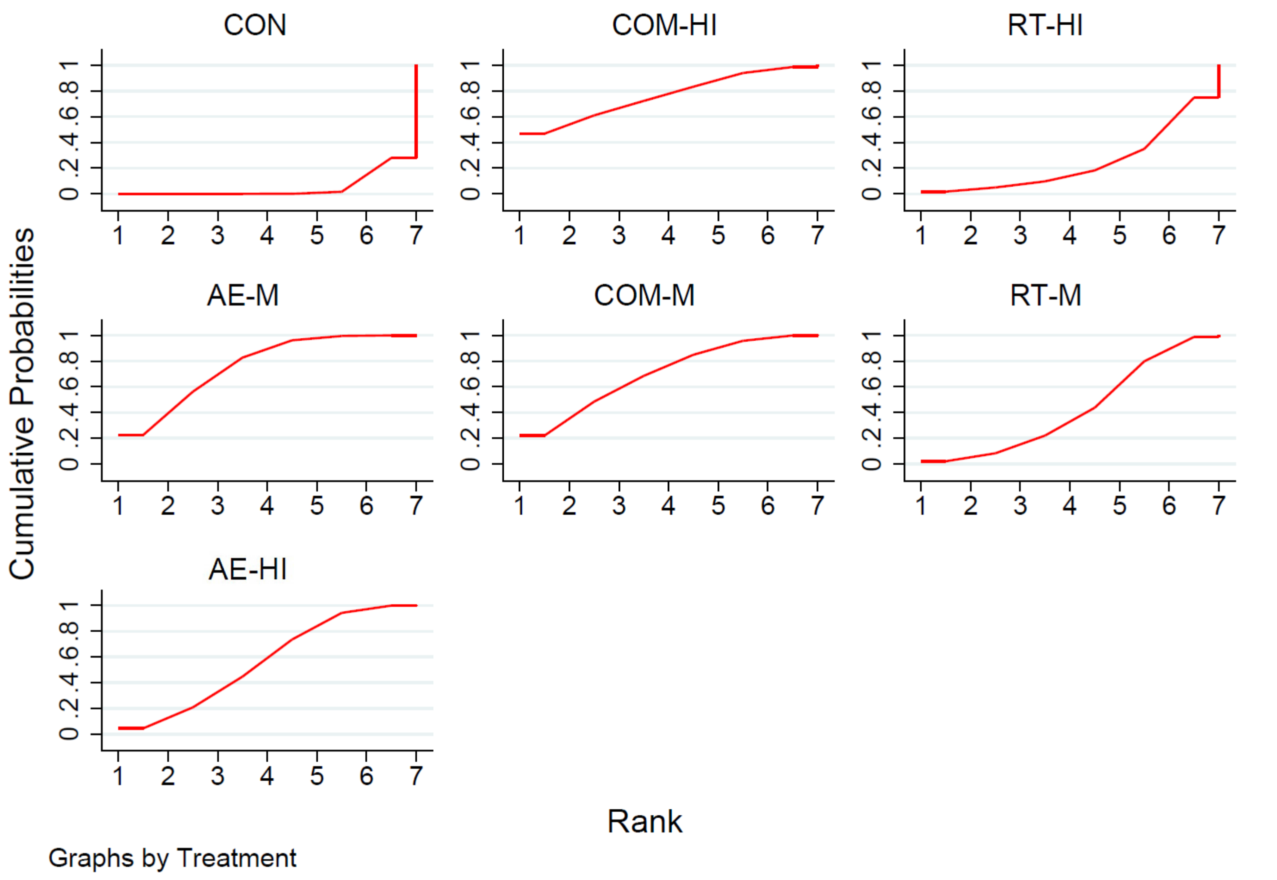

Figure 2. SUCRA values for body mass index

AE-M, moderate-intensity aerobic exercise
AE-HI, high-intensity aerobic exercise
RT-M, moderate-intensity resistance exercise
RT-HI, high-intensity resistance exercise
COM-M, moderate-intensity combined exercise
COM-HI, high-intensity combined exercise
CON, blank controls

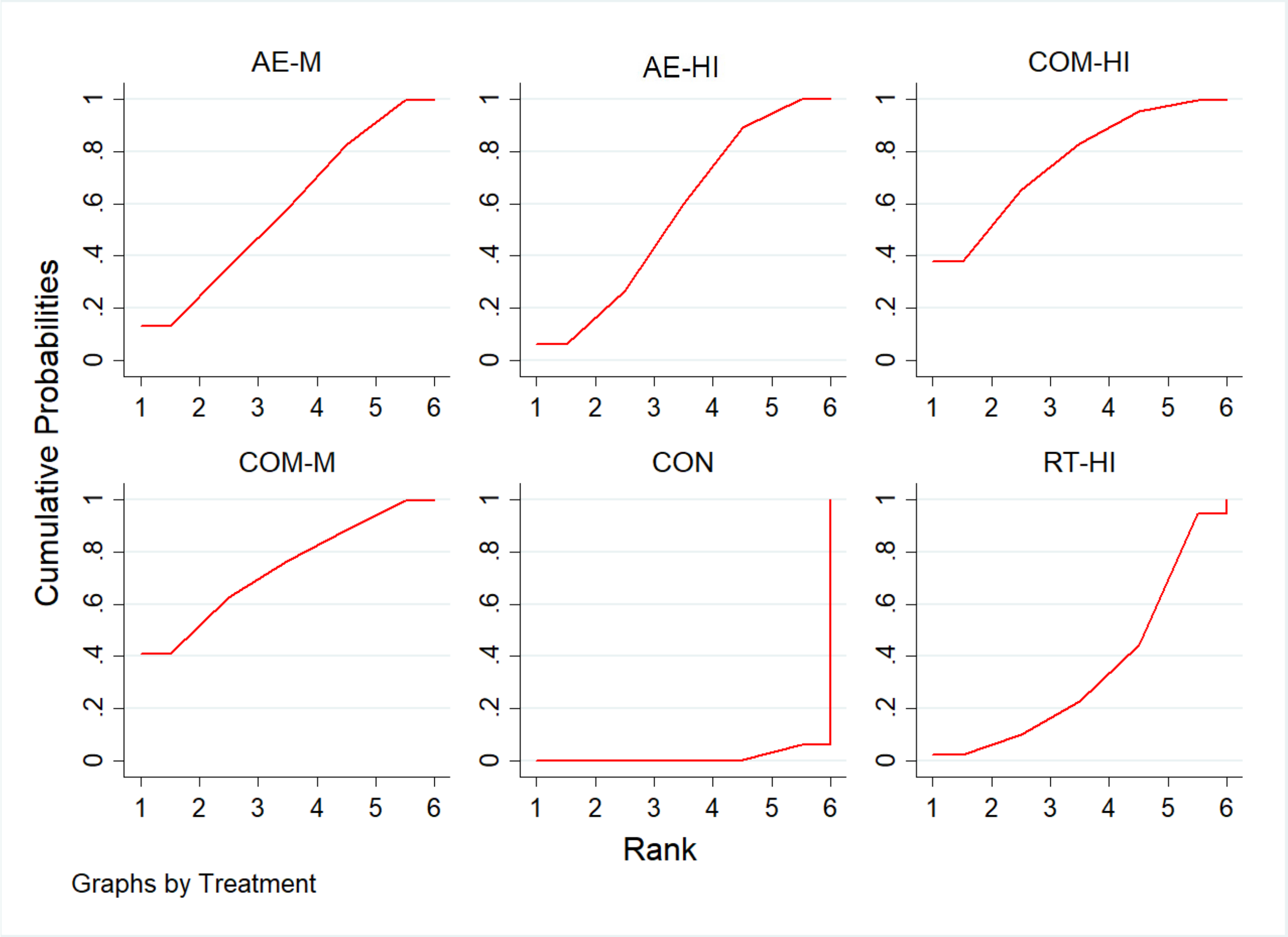

Figure 3. SUCRA values for fat mass

AE-M, moderate-intensity aerobic exercise
AE-HI, high-intensity aerobic exercise
RT-HI, high-intensity resistance exercise
COM-M, moderate-intensity combined exercise
COM-HI, high-intensity combined exercise
CON, blank controls

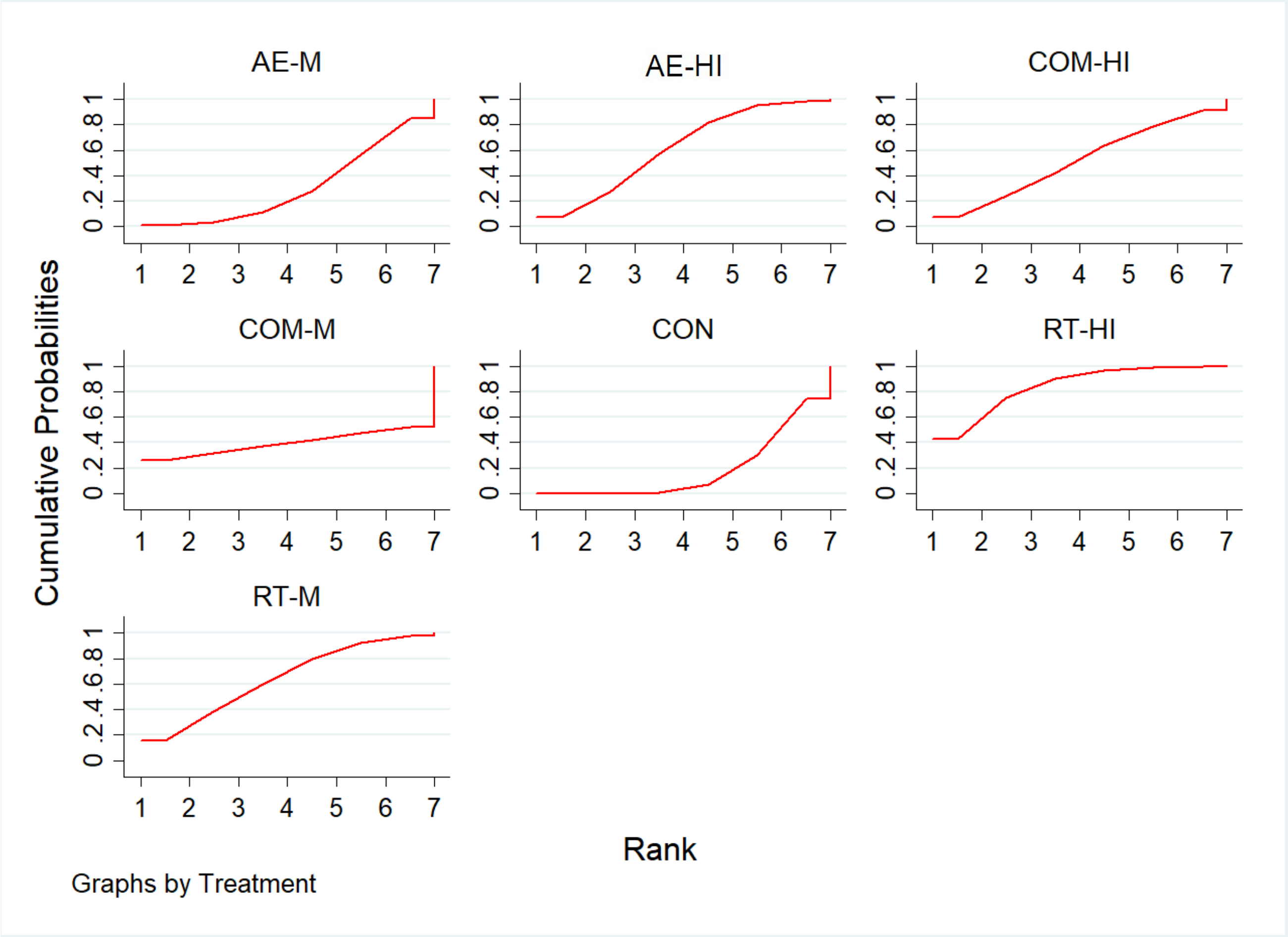

Figure 4. SUCRA values for fat free mass

AE-M, moderate-intensity aerobic exercise
AE-HI, high-intensity aerobic exercise
RT-M, moderate-intensity resistance exercise
RT-HI, high-intensity resistance exercise
COM-M, moderate-intensity combined exercise
COM-HI, high-intensity combined exercise
CON, blank controls

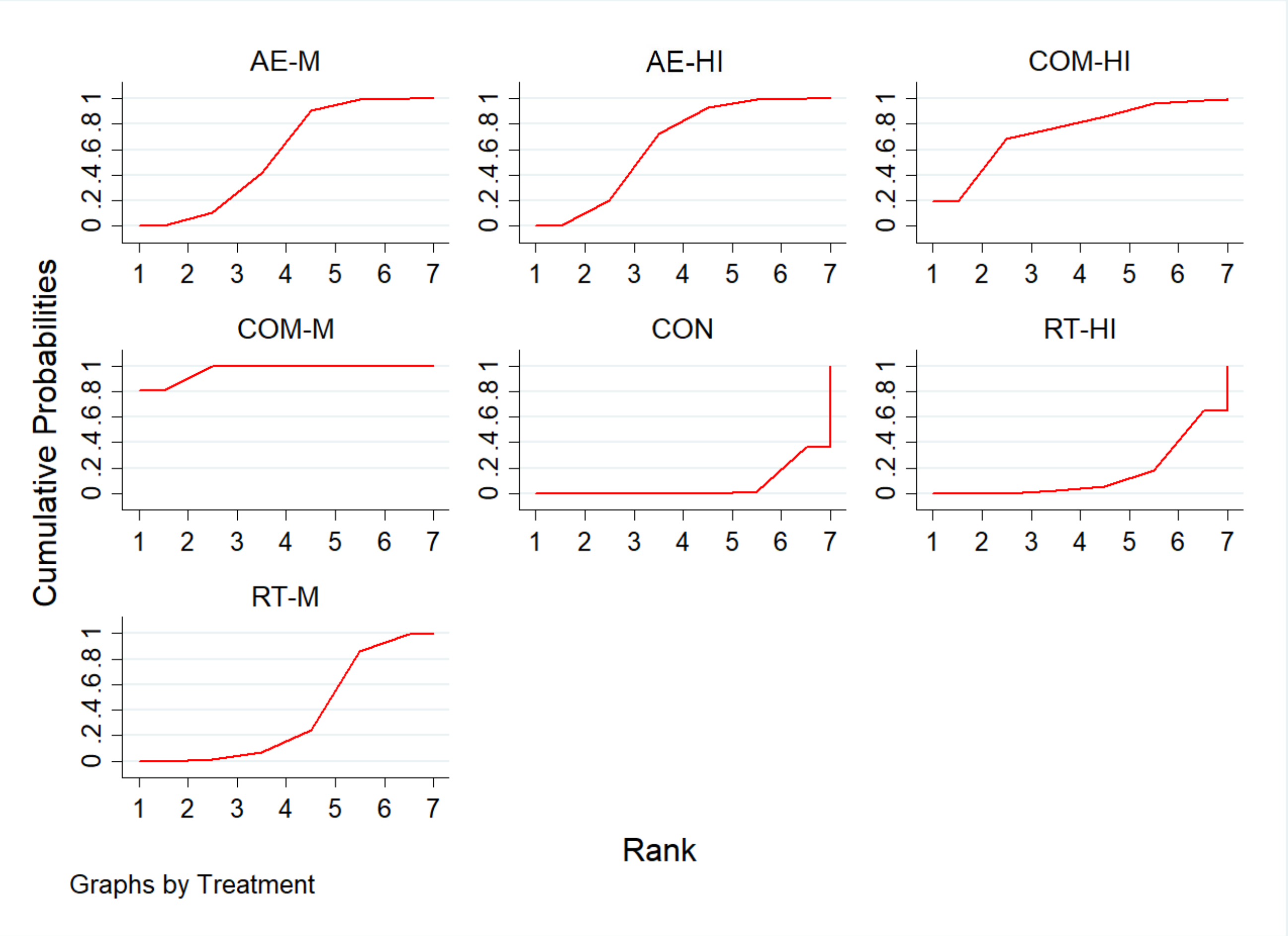

Figure 5. SUCRA values for weight

AE-M, moderate-intensity aerobic exercise
AE-HI, high-intensity aerobic exercise
RT-M, moderate-intensity resistance exercise
RT-HI, high-intensity resistance exercise
COM-M, moderate-intensity combined exercise
COM-HI, high-intensity combined exercise
CON, blank controls

Figure S9 Funnel Plot Graphics

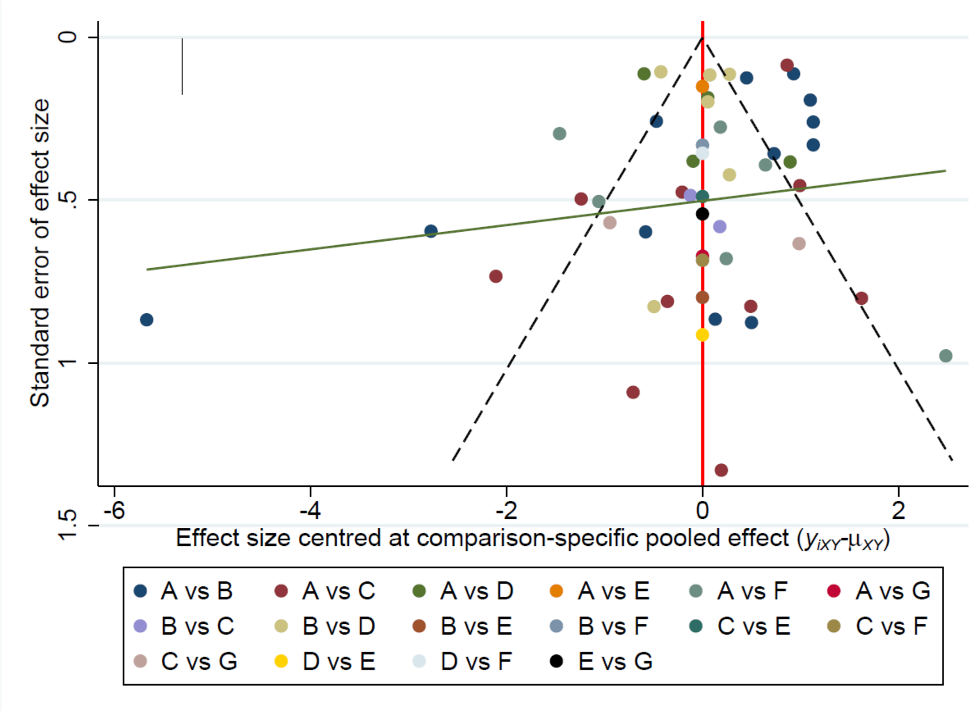

Figure 1. Funnel plot for body fat percentage

A, blank controls
B, moderate-intensity aerobic exercise
C, high-intensity aerobic exercise
D, moderate-intensity resistance exercise
E, high-intensity resistance exercise
F, moderate-intensity combined exercise
G, high-intensity combined exercise

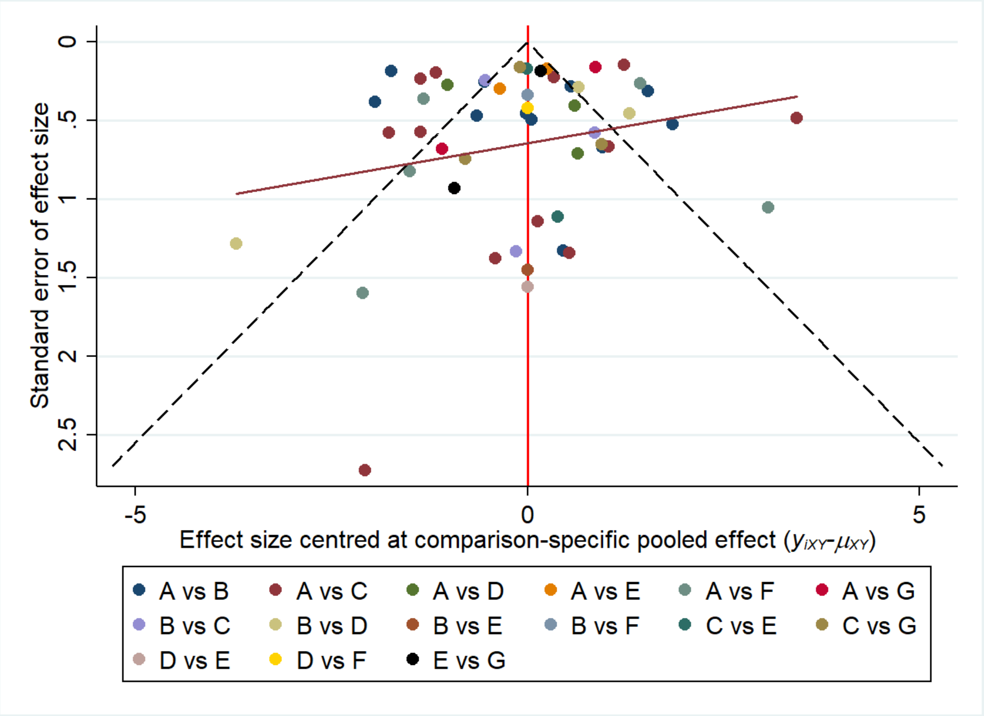

Figure 2. Funnel plot for body mass index

A, blank controls
B, moderate-intensity aerobic exercise
C, high-intensity aerobic exercise
D, moderate-intensity resistance exercise
E, high-intensity resistance exercise
F, moderate-intensity combined exercise
G, high-intensity combined exercise

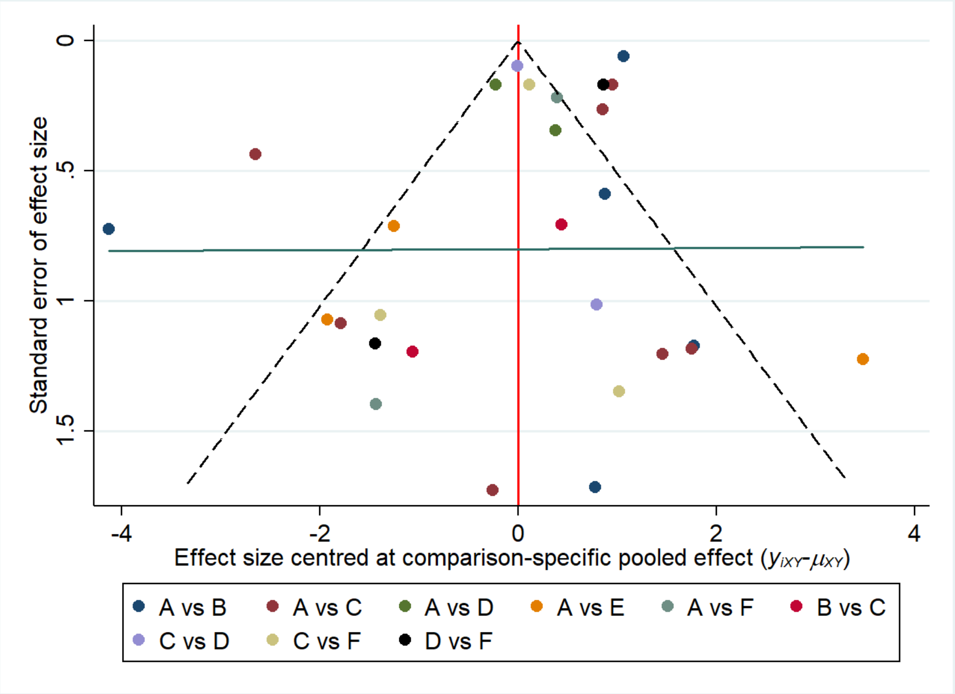

Figure 3. Funnel plot for fat mass

A, blank controls
B, moderate-intensity aerobic exercise
C, high-intensity aerobic exercise
D, high-intensity resistance exercise
E, moderate-intensity combined exercise
F, high-intensity combined exercise

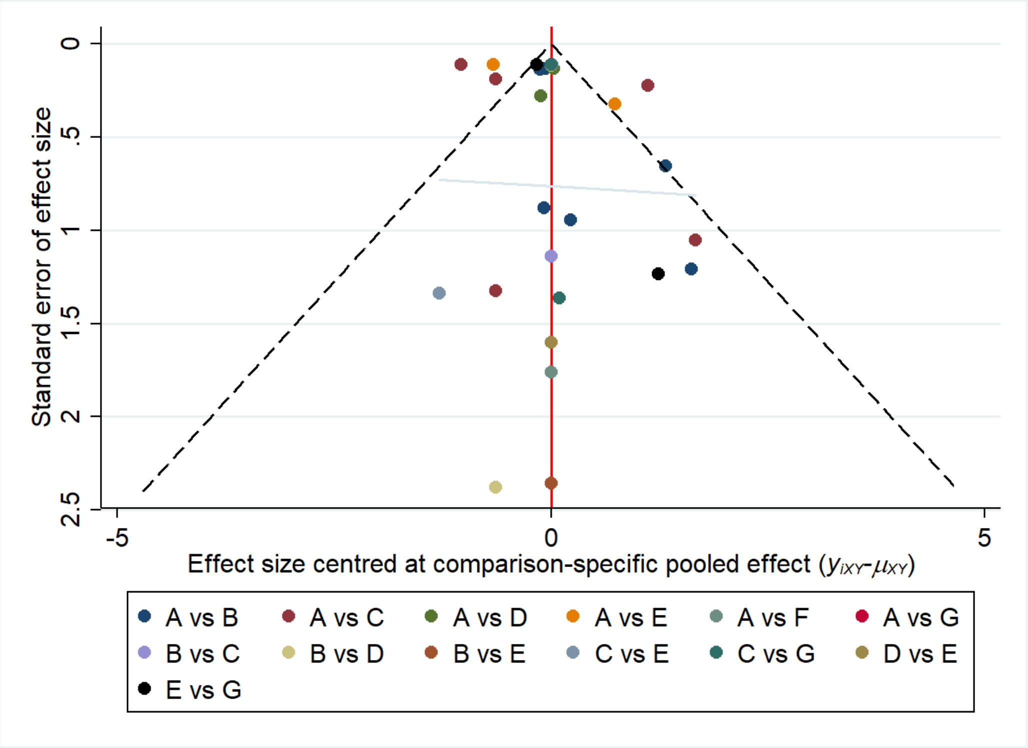

Figure 4. Funnel plot for fat free mass

A, blank controls
B, moderate-intensity aerobic exercise
C, high-intensity aerobic exercise
D, moderate-intensity resistance exercise
E, high-intensity resistance exercise
F, moderate-intensity combined exercise
G, high-intensity combined exercise

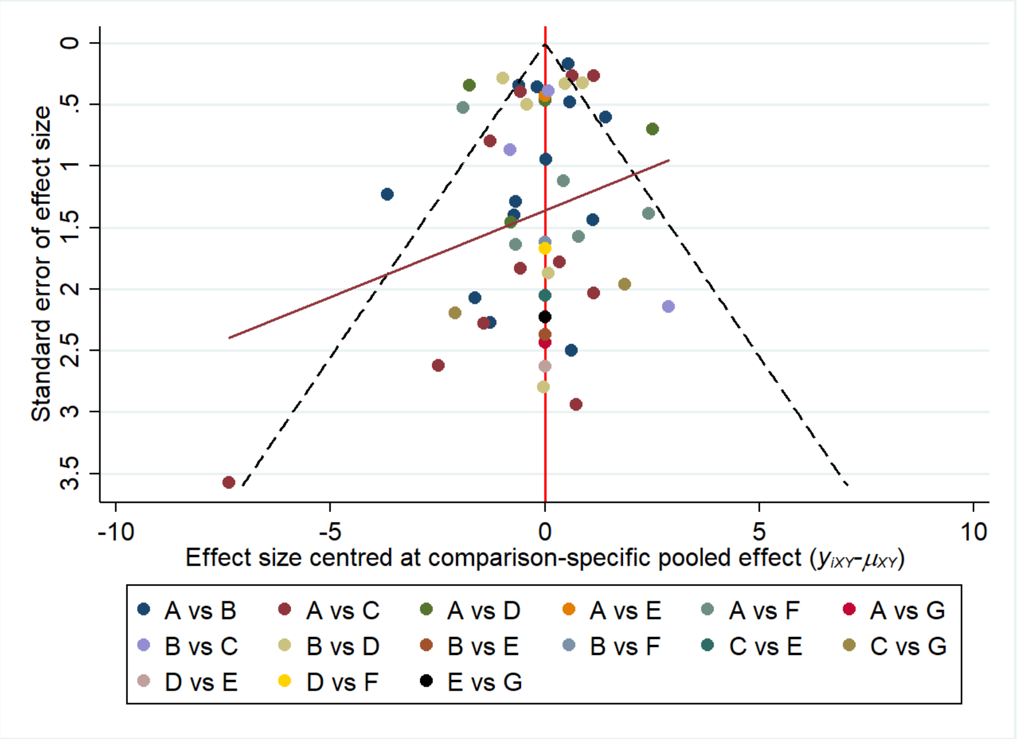

Figure 1. Funnel plot for weight

A, blank controls
B, moderate-intensity aerobic exercise
C, high-intensity aerobic exercise
D, moderate-intensity resistance exercise
E, high-intensity resistance exercise
F, moderate-intensity combined exercise
G, high-intensity combined exercise
